# Maytansinol Derivatives: Side Reactions as a Chance for New Tubulin Binders

**DOI:** 10.1002/chem.202103520

**Published:** 2021-11-29

**Authors:** Paola Marzullo, Zlata Boiarska, Helena Pérez‐Peña, Anne‐Catherine Abel, Beatriz Álvarez‐Bernad, Daniel Lucena‐Agell, Francesca Vasile, Maurizio Sironi, Karl‐Heinz Altmann, Andrea E. Prota, J. Fernando Díaz, Stefano Pieraccini, Daniele Passarella

**Affiliations:** ^1^ Department of Chemistry Università degli Studi di Milano Via Golgi 19 20133 Milan Italy; ^2^ Laboratory of Biomolecular Research Paul Scherrer Institute Forschungsstrasse 111 5232 Villigen PSI Switzerland; ^3^ Centro de Investigaciones Biológicas Margarita Salas Consejo Superior de Investigaciones Científicas Ramiro de Maeztu 9 28040 Madrid Spain; ^4^ Department of Chemistry and Applied Biosciences Institute of Pharmaceutical Sciences, ETH, Zürich Vladimir-Prelog Weg 4, HCI H405 8093 Zürich Switzerland

**Keywords:** maytansinol, maytansine binding site, tubulin, microtubules, tubulin binders

## Abstract

Maytansinol is a valuable precursor for the preparation of maytansine derivatives (known as maytansinoids). Inspired by the intriguing structure of the macrocycle and the success in targeted cancer therapy of the derivatives, we explored the maytansinol acylation reaction. As a result, we were able to obtain a series of derivatives with novel modifications of the maytansine scaffold. We characterized these molecules by docking studies, by a comprehensive biochemical evaluation, and by determination of their crystal structures in complex with tubulin. The results shed further light on the intriguing chemical behavior of maytansinoids and confirm the relevance of this peculiar scaffold in the scenario of tubulin binders.

## Graphical abstract

**Exploring the maytansinol** acylation reaction, we have discovered previously unreported structural modifications of the maytansine scaffold. This permitted the synthesis and full characterization of a series of novel maytansinoids. A comprehensive biochemical evaluation and X‐ray crystallography structure determinations of tubulin‐maytansinoids identified potent tubulin binders. Moreover, our investigation allowed us to evaluate the impact of the structural modifications described.

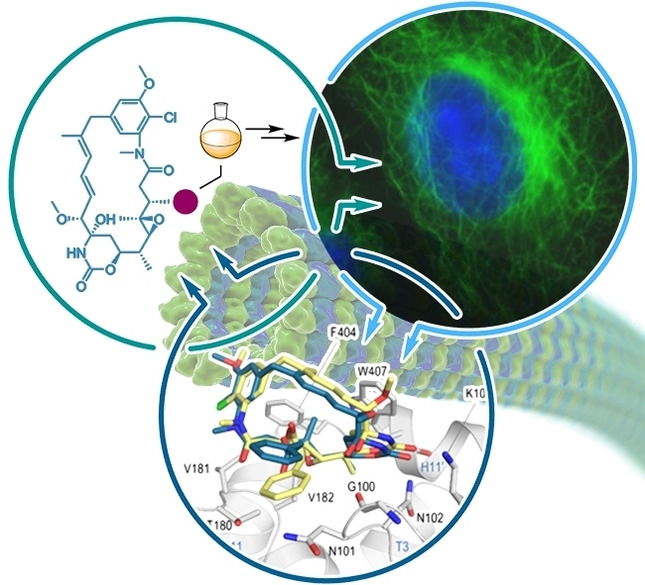

## Introduction

Maytansine (**1 a**) is an ansamacrolide isolated from *Maytenus ovatus*; it is a highly potent antimitotic agent that exerts an antiproliferative effect by inhibiting microtubule assembly upon binding to tubulin.[^1^, ^2^, ^3^] Despite a promising in vitro profile, clinical trials with maytansine in cancer patients failed because of poor efficacy and unacceptable systemic toxicity.[^4^, ^5^] Although the narrow therapeutic window precluded further clinical development of the parent compound, maytansine, its derivatives were successfully applied clinically as antibody‐drug conjugates (ADCs) and thus continue to excite interest.[^6^, ^7^, ^8^, ^9^, ^10^] This is mainly due their high cytotoxicity, much higher than that of vincristine and vinblastine.^[11]^

**Scheme 1:**
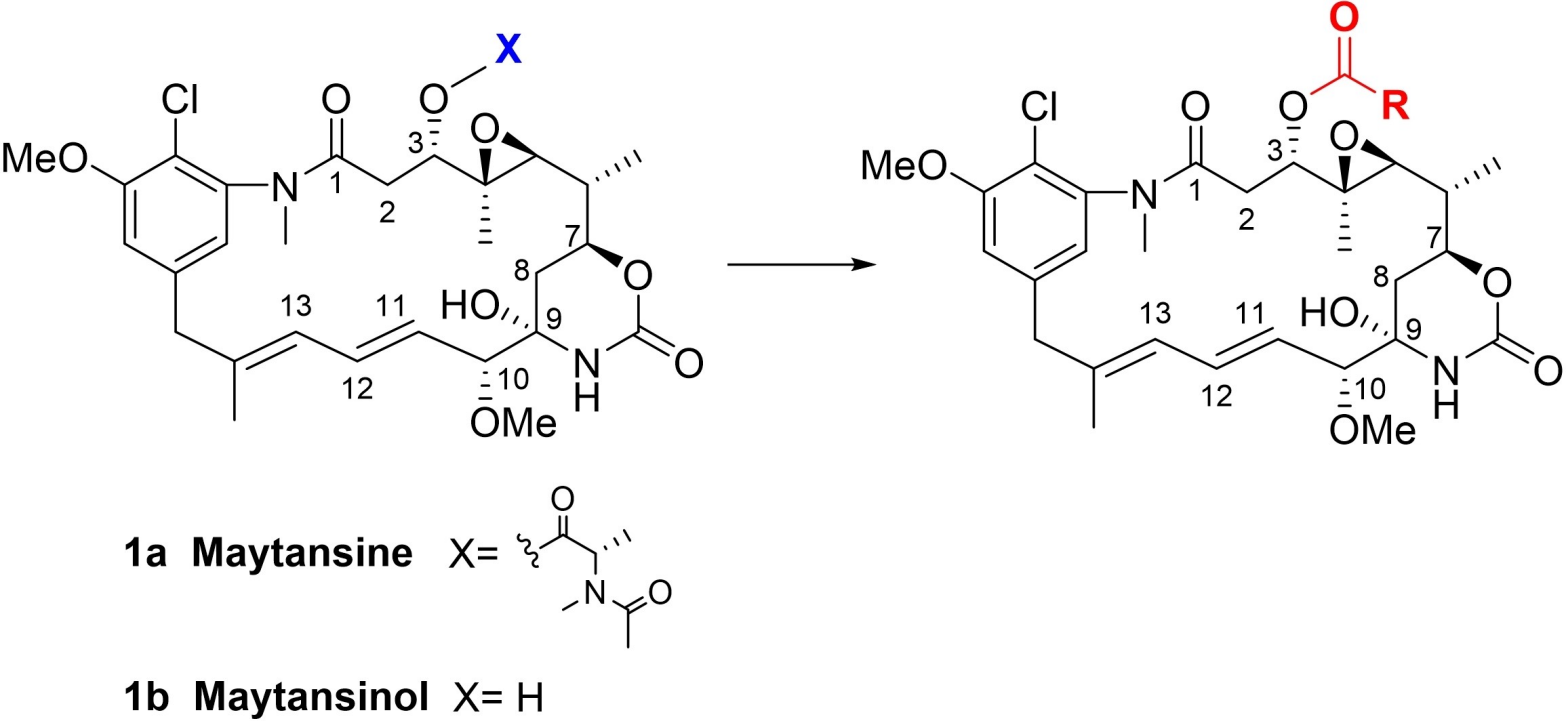
Structure of maytansine (**1 a**), maytansinol (**1 b**), and the generic acylation reaction of maytansinol.

Maytansinol (**1 b**) was first obtained by Kupchan et al. both by isolation from *Putterlickia verrucose* and chemical removal of the acyl group from the hydroxy group at the C3 position.^[11]^ It showed weaker inhibitory activity on tubulin polymerization than maytansine, thus implying that the ester moiety at the C3 position of ansamitocins, maytansine, and maytansinoids plays an important role for biological activity and cell permeability.[^12^, ^13^] In fact, it has just recently been found that the carbonyl oxygen atom of the ester moiety forms a strong intramolecular interaction with the hydroxy group at position 9, fixing the bioactive conformation.^[14]^ Maytansinol has been regarded as a valuable precursor because acylation allows the preparation of both natural and new semisynthetic maytansinoids, differing in the ester side‐chain substituents (Scheme 1).[^15^, ^16^] The acylation reaction of maytansinol is a crucial step in the preparation of maytansinoid ADCs or nanoparticles, constituting an uprising class of targeted cancer therapeutics.[^17^, ^18^, ^19^, ^20^, ^21^] A few attempts to conjugate maytansinoids to peptides by this reaction have also been made very recently.[^22^, ^23^] Furthermore, considering that the maytansine binding site is one of the most recently identified and least explored on tubulin, acylation of maytansinol may serve for the preparation of useful molecular probes to better understand the structure‐activity relationships of maytansinoids or to identify new maytansine‐site ligands.[^24^, ^25^]

The potent biological activity of maytansinoids, the variety of possible applications, and above all the intriguing macrocyclic structure decorated by several sensitive functional groups, motivated us to study the formation of maytansinol derivatives induced by different reaction conditions with particular attention to the acylation reaction.

Pursuing our interest in the chemistry and biological activity of tubulin binders,[^26^, ^27^, ^28^, ^29^] we report here a set of new maytansinoids, obtained as a result of maytansinol acylation reaction. Apart from the formation of esters at C3 position, maytansinol has been discovered to undergo a range of other structural transformations not previously reported. To evaluate the potential of our novel maytansinoids, we submitted the obtained compounds to a) computational studies, b) experiments for evaluation of the effect on tubulin polymerization dynamics, c) evaluation of the cytotoxicity and d) structure determination by X‐Ray crystallography. The obtained results are crucial for both the design and the synthesis of new effective maytansinoids.

## Results and Discussion

### Chemical synthesis

**Figure 1:**
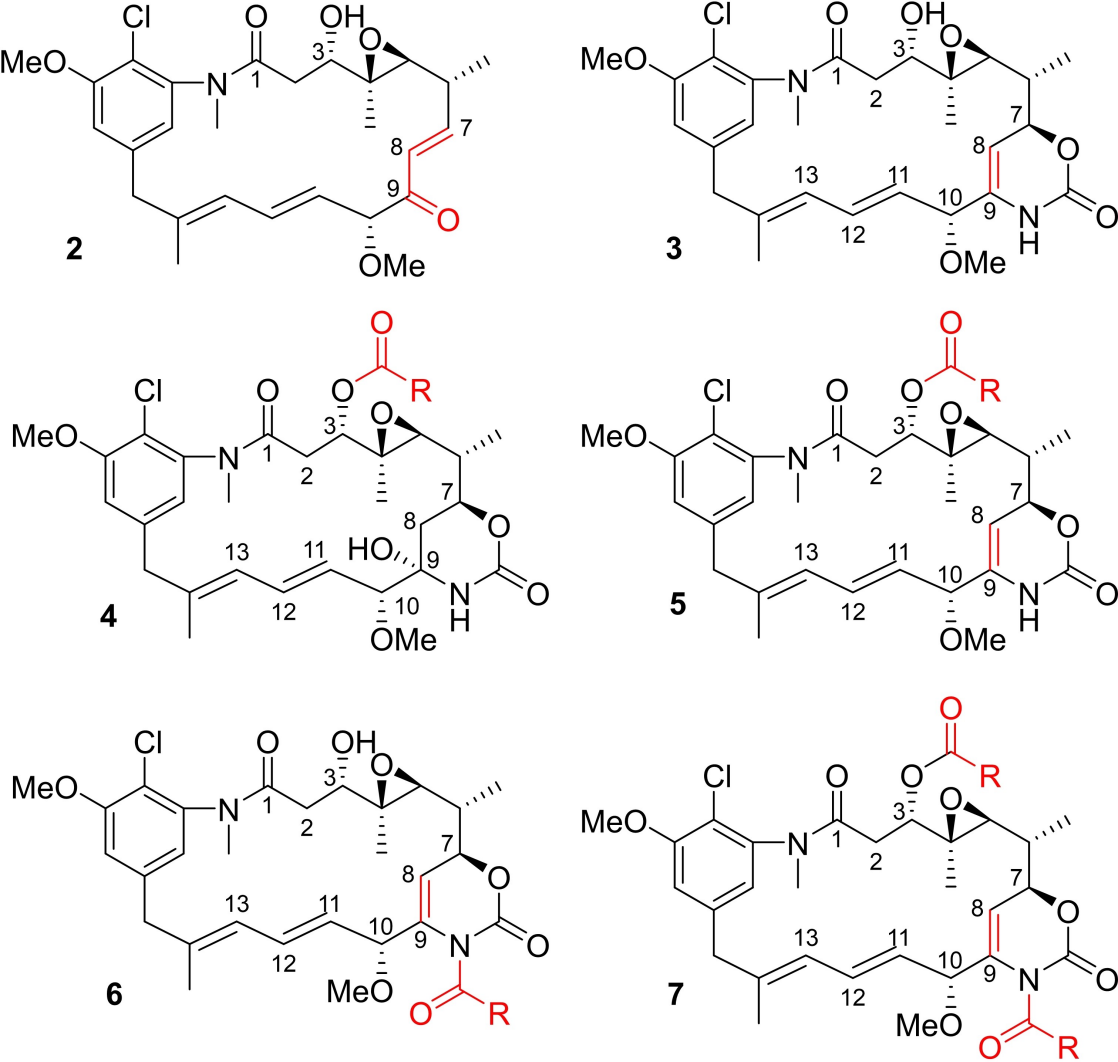
General structures of the new maytansinol derivatives obtained.

Initially, maytansinol was subjected to alkylation by employing propargyl bromide as the alkylating agent. The reaction was performed in ACN/DMF using Cs_2_CO_3_, KI and TEBA as a phase‐transfer catalyst. Interestingly, the 4‐hydroxy 2‐oxazinanone of maytansinol was cleaved providing the unknown unsaturated ketone **2**. Repeating the same reaction without the addition of alkylating agent did not lead to the formation of any product, thereby suggesting that the elimination can occur only after the oxazinanone nitrogen alkylation that induces the formal release of carbon dioxide, water and propargyl amine. We then moved our attention to the acylation at C3 position. Due to the steric hindrance and the consequent poor reactivity of the maytansinol secondary alcohol, the first synthetic strategy was the use of acyl chlorides. Common aliphatic and aromatic acyl chlorides were studied in order to evaluate the influence of the nature of the side chain. In addition to the formation of the expected O‐acylated product **4**, other undescribed maytansinoids were formed (Figure 1; **3**–**7**). The modifications included also the formation of the new double bond in C8‐C9 position, as a consequence of the dehydration of the hydroxy group in the oxazinanone (**3**, **5**–**7**), and the acylation of the oxazinanone nitrogen (**6**, **7**). Attracted by the interesting structural transformations, we planned to monitor their formation depending on the conditions and acylating agents used.

The use of benzoyl chloride as acylating agent in presence of triethylamine and 4‐pyrrolidinopyridine did not secure the obtainment of only the desired ester, but led to a mixture of compounds **3 a**, **4 a**, **6 a** and **7 a** depending on the molar ratio and reaction time. Lowering the temperature (−20 °C) led only to the formation of **3 a** in very low yield, while the use of pyridine as solvent led to the formation of **4 a** in 25 % yield.

Increasing the amount of benzoyl chloride shifted the composition of the reaction mixture exclusively in favor of the formation of **6 a** and **7 a**.

**Figure 2:**
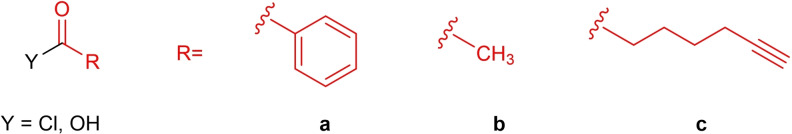
Structures of the acyl groups and acylating agents used.

In contrast, the use of aliphatic acyl chloride, such as **b** and **c** (Figure 2), predominantly produced the derivative **4**, showing a weak tendency to form the corresponding dehydrated compounds.

The common Steglich esterification procedure was applied using different coupling reagents. The use of a large excess of dicyclohexylcarbodiimide (DCC)/4‐dimethylaminopyridine; (DMAP) with a reaction time of 3–4 h gave positive results for obtaining the desired C3 acylated derivatives (**4 a**–**c**). The selectivity was improved by adding an excess of ZnCl_2_, and compound **4** was preferentially obtained, even if the reaction time increases. Stoichiometric amount of DCC with 4‐(dimethylamine)pyridinium 4‐toluenesulfonate (DPTS) as acyl‐transfer agent instead of DMAP did not make the reaction selective, moreover the kinetics resulted very slow. The use of excess DCC affected the purity of the obtained products due to the difficult removal of the DCU by‐product. The use of the EDC as coupling agent solved this problem and reduced the formation of the dehydrated products although it required a longer reaction time of 18–24 h.

In general, a longer reaction time increased the conversion of maytansinol, thereby favoring the formation of products **5** and **7**. In summary, the use of different acylating agents (Figure 2), coupling reagents, reaction conditions, and reaction times showed a significant influence on the formation of diverse products, giving the possibility to shift the product formation preference (Table 1).

**Table 1:** Reaction conditions used for the acylation reaction in dichloromethane (DCM) at room temperature.

R	Y	Acyl eqiv.	C.A.	Base	*t* [h]	Yield [%]					
						**1 b**	**3**	**4**	**5**	**6**	**7**
**a** ^[a]^	Cl	0.5	–	TEA, A	0.5	82	5	–	–	–	–
**a** ^[a]^	Cl	1	–	TEA, A	2	80	15	2	–	–	–
**a**	Cl	1	–	TEA, A	6	75	11	11	–	–	–
**a**	Cl	4	–	TEA, A	4	–	–	–	–	33	67
**a** ^[b]^	Cl	6	–	Py	5	51	–	25	–	–	–
**a**	OH	3	DCC	DMAP	8	4	5	35	26	–	18
**a**	OH	6	DCC	DMAP	48	–	–	–	15	–	31
**a** ^[c]^	OH	3	DCC	DMAP, ZnCl_2_	48	29	–	62	4	4	–
**a** ^[c]^	OH	1	DCC	DPTS	5d	55	4	18	14	5	4
**a**	OH	3	EDCI	DMAP, TEA	24	21	8	39	12	–	7
**a**	OH	3	EDCl	DMAP, TEA	48	–	–	15	47	–	20
**b**	Cl	2	–	TEA, A	6	51	–	46	–	–	‐
**b**	OH	3	DCC	DMAP	4	–	–	42	43	–	‐
**c**	Cl^[24]^	8	–	TEA, A	7d	65	2	16	7	–	‐
**c**	OH	3	DCC	DMAP	3	7	9	37	30	–	6
**c**	OH	3	EDCl	DMAP, TEA	18	17	4	36	15	–	6

[a] Reaction performed at −20 °C; [b] Reaction performed in Py; [c] Percentage determination by HPLC; TEA=triethylamine; A=4‐pyrrolidinopyridine; Py=pyridine; EDCl=1‐ethyl‐3‐(3‐dimethylaminopropyl)carbodiimide.

**Figure 3:**
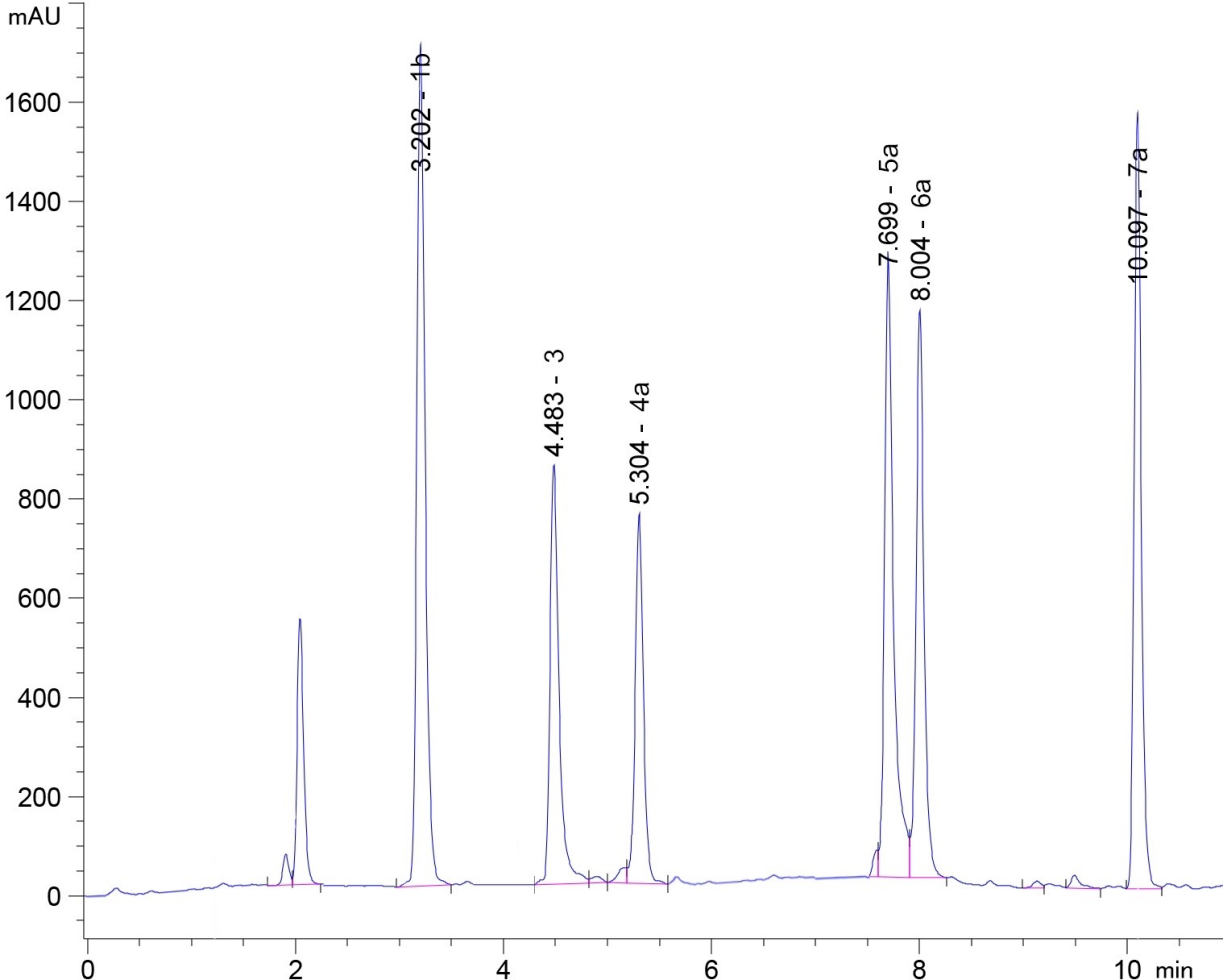
Representative HPLC chromatogram. ZORBAX SB C_8_ column (3.5 μm ×4.6×150 mm). Pressure: 85 bar; Flow rate: 1 mL/min. UV: 254 and 210 nm with DAD detection. Mobile phase: H_2_O/ACN 1 min isocratic at 50 % ACN, then gradient to 90 % ACN over 10 min. Retention times: **1 b**, 3.10 min; **3**, 4.08 min; **4 a**, 5.20 min; **5 a**, 6.90 min; **6 a**, 7.20 min; **7 a**, 9.40 min.

An HPLC method was refined in order to follow easily the conversion and to determine the composition percentage of the reaction mixture (Figure 3).

The spectroscopic characterization of the obtained derivatives required a detailed and sophisticated investigation. All compounds discussed were fully characterized using NMR data, and the complete ^1^H and ^13^C NMR assignments (Tables S1–S4 in the Supporting Information) have been determined based on 1D and 2D NMR spectra (^1^H and ^13^C NMR, COSY, HSQC, and HMBC). Diagnostic NMR peaks are listed in Table 2. The evaluation of the main differences between **1 b** and the O‐acylated derivative **4**, **5**, **7** shows an evident shift of H‐3 signal from 3.34 to about 5 ppm as a consequence of the successfully esterification at the OH‐3. It is possible to observe the shift of the H‐2 signals from 2.36 and 1.91 ppm to about 2.9 and 2.2 ppm. The corresponding signals of the compounds **3** and **6** did not undergo significant changes excluding an involvement of the hydroxy group. The presence of C8=C9 double bond in compounds **3** and **5**–**7** was confirmed by the merge of the H‐8 signals in the range of 4.5–5.3 ppm. Furthermore, the characteristic H‐7 signal was shifted at lower field and the multiplicity changes to a *dd* for **3** and **5**, whereas a multiplet was observed for **6** and **7**. Similarly, it is possible to note a new revealing of the H‐10 over 4.15 ppm, whereas the olefinic protons signals H‐11, H‐12, and H‐13 are all shifted slightly to higher fields. Finally, the disappearance of the NH signal indicates an acylation of oxazinanone as regard the compounds **6** and **7**.

**Table 2:** Diagnostic ^1^H NMR spectroscopic data of maytansinoid compounds.

Atom	**1 b** ^[a]^	**3** ^[b]^	**4** ^[a]^	**5** ^[a]^	**6** ^[b]^	**7** ^[a]^
2	2.36, 2.13	2.32, 2.14	2.88–2.79, 2.26	2.91, 2.22	2.37, 2.12	2.99–2.87, 2.27
3	3.55	3.59	5.04–4.85^c^	5.02	3.61	5.04
7	4.25	4.78	4.31–4.14	4.56	4.86	4.92–4.76^[d]^
8	1.91, 1.41	5.02–4.96	1.59, 1.54–1.39	4.43	5.43–5.33	4.92–4.76^[d]^
10	3.64	4.20–4.12	3.50	4.15	4.42	4.35
11	5.52	5.54	5.04–4.85^[c]^	4.88	5.54	4.92–4.76^[d]^
12	6.70	6.46	6.61	6.54	6.48	6.64
13	6.20	6.16	6.04	6.13	6.15	6.12
OH‐3	4.50		–	–		–
OH‐9	4.80	–	4.47	–	–	–
NH	6.42	7.08	6.33	8.03	–	–

Chemical shifts (ppm) were determined with reference to TMS; Spectra determined at 400 MHz; [a] Solvent is [D_6_]acetone; [b] Solvent is deuterated chloroform; [c], [d] Chemical shifts bearing the same symbol overlap.

The intriguing structural novelty of the obtained compounds moved us to evaluate their biological activity in combination with their ability to interact with tubulin, with the aim to improve the knowledge on the structure‐activity relationships of maytansinoids.

### Computational studies

We used docking to predict the spatial coordinates of the binding mode acquired by the synthesized maytansinoids within the maytansine site, which is located in a shallow pocket on β‐tubulin facing the inter‐dimer interface.^[25]^

To test the accuracy of the docking engine AutoDock Vina, the crystallographic structure of maytansine bound to β‐tubulin was redocked in its site as a positive control measure. As a result, the geometry assigned by AutoDock Vina for maytansine overlapped with its crystallographic orientation. Therefore, since AutoDock Vina successfully reproduced the crystallographic findings, we could confirm the reliability of this docking software.

Subsequently, we successfully performed the docking of the derivatives **3**, **4 a**–**c** and **5 a**–**c** to the maytansine binding site (Table 3). In all cases, the orientation of the maytansinol ring remained in the same spatial arrangement, acquiring a similar binding mode to the parent compound. The introduction of bulky substituents at position XO did not alter the predicted 3D arrangement of the core of the molecule. Thus, we assumed that binding of maytansinoids to β‐tubulin is very tolerant to modifications of the X‐hydroxy group and expected that the binding mode of the investigated molecules resembles the one of the parent compound.

**Table 3:** Protein–ligand free energies of binding of the best docking geometries for each maytansinoid returned by AutoDock Vina calculations when docking to the structure of β‐tubulin 4TV81.

Maytansinoid	**3**	**4 a**	**4 b**	**4 c**	**5 a**	**5 b**	**5 c**
Δ *G* ^0^ [kcal/mol]	−7.5	−7.4	−8.2	−6.7	−6.8	−7.5	−7.4

### Biological evaluation

To evaluate the activity of the representative compounds **2**, **3**, **4 a**–**c**, **5 a**–**c**, **6 a** and **7 a** on tubulin and microtubules, we first probed their effect on microtubule assembly dynamics, and subsequently determined their binding affinities to tubulin dimers and their cell toxicity.

### Inhibition of tubulin assembly

**Figure 4:**
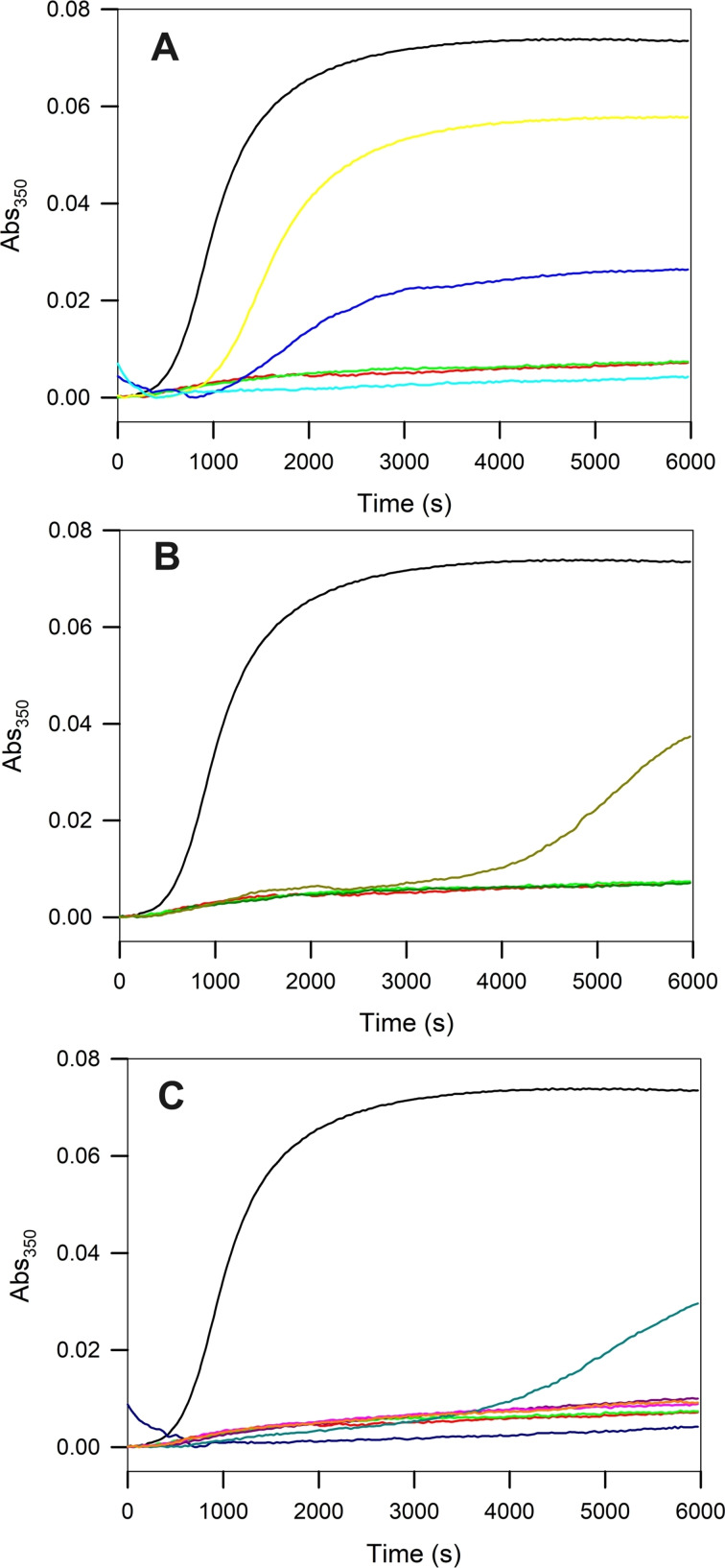
Inhibition of tubulin assembly activity by selected compounds. All experiments were performed in triplicate. Time courses of assembly of 25 μM tubulin in GAB in the presence of vehicle (DMSO; black lines), 27.5 μM maytansine **1 a** (red lines), or maytansinol **1 b** (green lines), or A) **2** (yellow), **6 a** (cyan), **7 a** (blue); or B) **3** (dark yellow), **4 a** (dark red); or C) **4 b** (pink), **4 c** (orange), **5 a** (dark blue), **5 b** (dark pink), **5 c** (dark cyan).

Maytansine site targeting agents inhibit tubulin assembly by capping the plus end of β‐tubulin subunits, thereby precluding further microtubule growth.^[25]^ We therefore tested the selected compounds for their ability to inhibit tubulin assembly (Figure 4). Maytansine (**1 a**), which completely abolishes tubulin polymerization, and maytansinol (**1 b**), which has a noticeable effect at stoichiometric concentrations, were used as controls.

All the compounds assayed inhibited tubulin assembly into microtubules at stoichiometric ratios with tubulin, however, they showed different potencies. While compounds **4 a**–**c**, **5 a**, **5 b**, and **6 a** were strong inhibitors that completely abolished microtubule assembly, compounds **3**, **5 c**, and **7 a** showed only a mild and compound **2** **a** weak inhibition.

### Binding affinities

**Figure 5:**
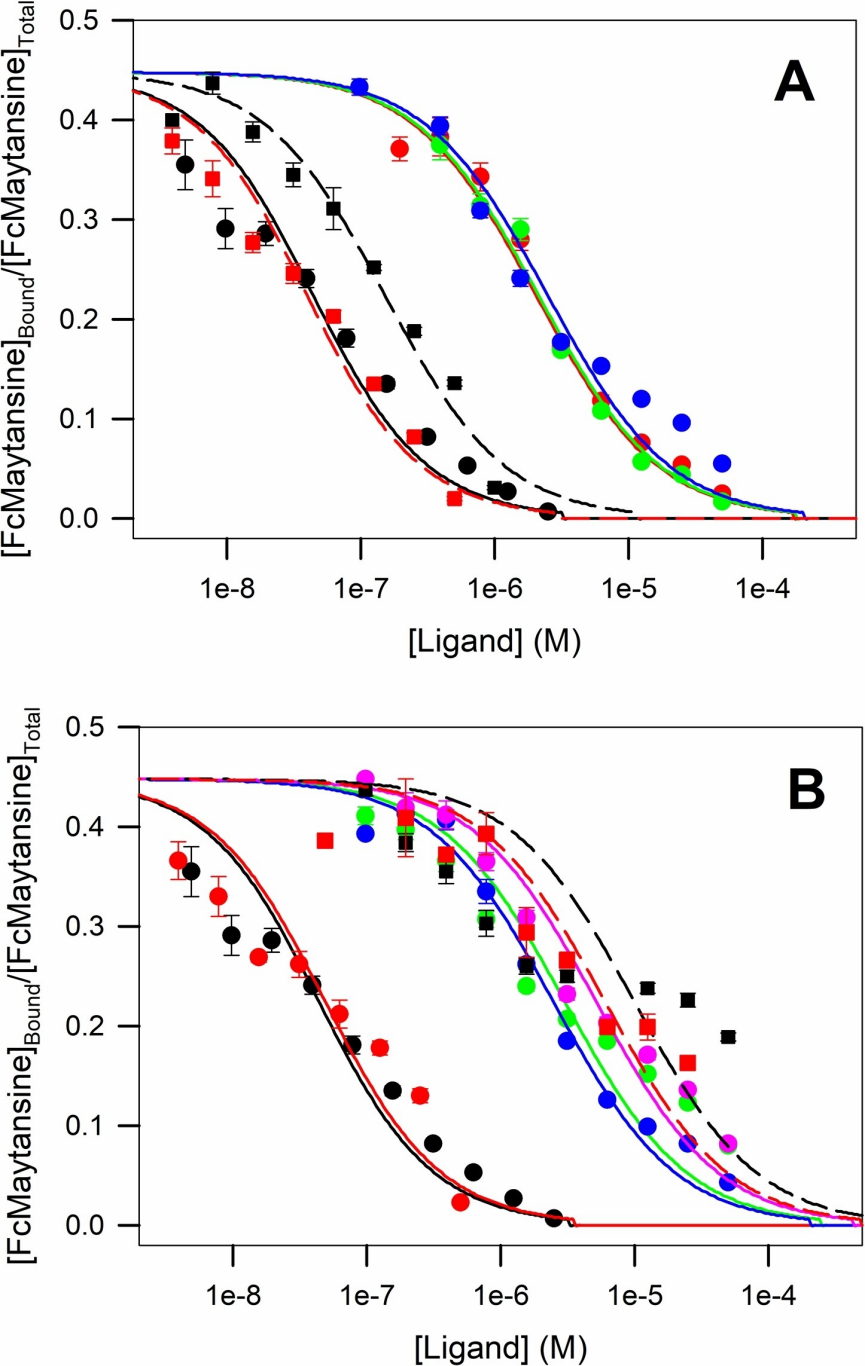
Determination of the binding constant of the ligands. Displacement of Fc‐maytansine assays for the ligands. A) Maytansine **1 a** (–•–), maytansinol **1 b** (–•–), **2** (–•–), **3** (–•–), **4 a** (‐ ‐▪‐ ‐), **4 b** (‐ ‐▪‐ ‐). (B) Maytansine **1 a** (–•–), **4 c** (–•–), **5 a** (–•–), **5 b** (–•–), **5 c** (–•–), **6 a** (‐ ‐▪‐ ‐), **7 A** (‐ ‐▪‐ ‐). The data are from three independent experiments and represent mean±SEM. The solid lines represent fits to the data (see the Methods in the Supporting information).

In order to correlate the tubulin assembly inhibition with the binding affinities of the compounds for the maytansine site were determined using competition against Fc maytansine^[24]^ (Table 4; Figure 5).

**Table 4:** Binding affinities of maytansinoid compounds.

Compound	Mean *K*_b_ [M^−1^]	*K*_d_ [nM]
**1 a**	9.0±1.3×10^7^	14±2
**1 b**	1.30±0.06×10^6^	780±40
**2**	1.42±0.08×10^6^	720±50
**3**	1.20±0.03×10^6^	830±20
**4 a**	2.0±0.×10^7^	51±3
**4 b**	9±1×10^7^	11±1
**4 c**	5.4±0.5×10^7^	20±2
**5 a**	9.2±0.3×10^5^	1090±40
**5 b**	1.17±0.04×10^6^	860±30
**5 c**	5.6±0.4×10^5^	1800±120
**6 a**	6±2×10^5^	3000±100
**7 a**	5.0±0.5×10^5^	2000±200

Surprisingly and unlike for other microtubule targeting agents, the potency did not correlate well with the determined binding affinities. The reason for this is that all the compounds assayed have at least micromolar affinity. Tubulin assembly inhibition is dependent on two factors. First, the binding affinity of the compound for the site has to be significant at the concentrations of the inhibition assay. As the concentrations of both tubulin and drugs employed in the assay were nearly one order of magnitude higher than the weakest dissociation constant measured for **6 a** (3 mM), we expected that all the ligands employed were bound to the protein. Therefore, the binding affinity should not influence the in vitro assembly inhibition activity in the way we observed.

According to the mechanism of action proposed by Prota et al.,^[25]^ ligand binding to the maytansine site should have a strong influence on MT‐assembly. Maytansine site ligands bind at a shallow pocket at the top of the tubulin β‐subunit where the interaction with a longitudinal aligned tubulin dimer in the protofilament takes place, thereby inhibiting the addition of tubulin subunits at the plus ends of growing microtubules.

From the ligands studied, **4 b** (11 nM) and **4 c** (20 nM) showed the highest affinities with dissociation constants close to the one of maytansine (14 nM), thus indicating that both small and bigger substituents can easily replace the *N*‐acetyl‐*N*‐methyl‐l‐alanine. On the other hand, compound **4 a** with a phenolic ester at position C3 was a slightly weaker binder (51 nM). The series of compounds lacking the hydroxy group at position C9 showed binding affinities in the 1 μM range (**2** 720 nM, **3** 830 nM, **5 a** 1090 nM, **5 b** 860 nM), thereby suggesting that the C9‐hydroxy group might serve as a critical anchoring point to allow establishing the interaction with the site. Finally, the remaining three compounds also lacking the hydroxy group at position C9 with modifications either at the C3, the oxazinanone nitrogen or both, displayed affinities in the sub‐mM range: **5 c** (1800 nM), **6 a** (3000 nM) and **7 a** (2000 nM).

In summary, two modifications resulted in a strong impact on the binding affinity, namely the lack of esterification of the C3‐hydroxy group (**1 b** maytansinol) and the elimination of the C9‐hydroxy group. Moreover, changes in the acid esterification at the C3 were non‐relevant, while the amidation at the oxazinanone‐nitrogen did not restore the affinity.

### Cytotoxicity

To correlate the potency of binding with the toxicity, and to investigate the potential of the compounds to overcome membrane pumps mediated multidrug resistance, we determined the cytotoxicity of the compounds both in A549 (small cell lung carcinoma) and in the isogenic pair A2780/A2780AD (pGp overexpressing) cell lines (Table 5).

**Table 5:** IC_50_ of maytansinoid compounds in A549 and A2780/A2780AD cell lines.

Cmpd		IC_50_ [nM]		R/S
	A549	A2780	A2780AD	
**1 a**	0.278±0.04	0.31±0.02	19.5±1.14	29
**1 b**	60±3	23.78±1.65	1459.94±148.27	
**2**	15±2	9.64±0.97	981.11±200.76	
**3**	28±2	74±4	>19706	
**4 a**	0.24±0.03	0.11±0.01	11±1	100
**4 b**	1.2±0.3	0.25±0.01	20±1	80
**4 c**	0.07±0.008	0.033±0.003	4.7±0.6	142
**5 a**	>570	338.40±4.65	2626.48±186.56	
**5 b**	190±40	130±20	>6156	
**5 c**	320±40	225±15	>7956	
**6 a**	>1030	2088.08±99	13477.38±622.59	
**7 a**	47±6	241,27±32.14	3495.97±319.71

Cytotoxicity requires effective binding of a ligand to tubulin at concentrations, which are about one order of magnitude lower than the dissociation constants of the corresponding ligand.^[30]^

The cytotoxicities determined in this study correlated well with the binding affinities: compounds with high affinity **4 a**–**c** behaved nearly as maytansine, showing nano‐ to sub‐nanomolar cytotoxicities, while compounds with sub‐micromolar and micromolar affinities were less cytotoxic. However, our data also highlight that the compounds were better substrates of pGp than maytansine, displaying higher resistance indexes than the parental compound.

### Effects on tubulin cytoskeleton

**Figure 6:**
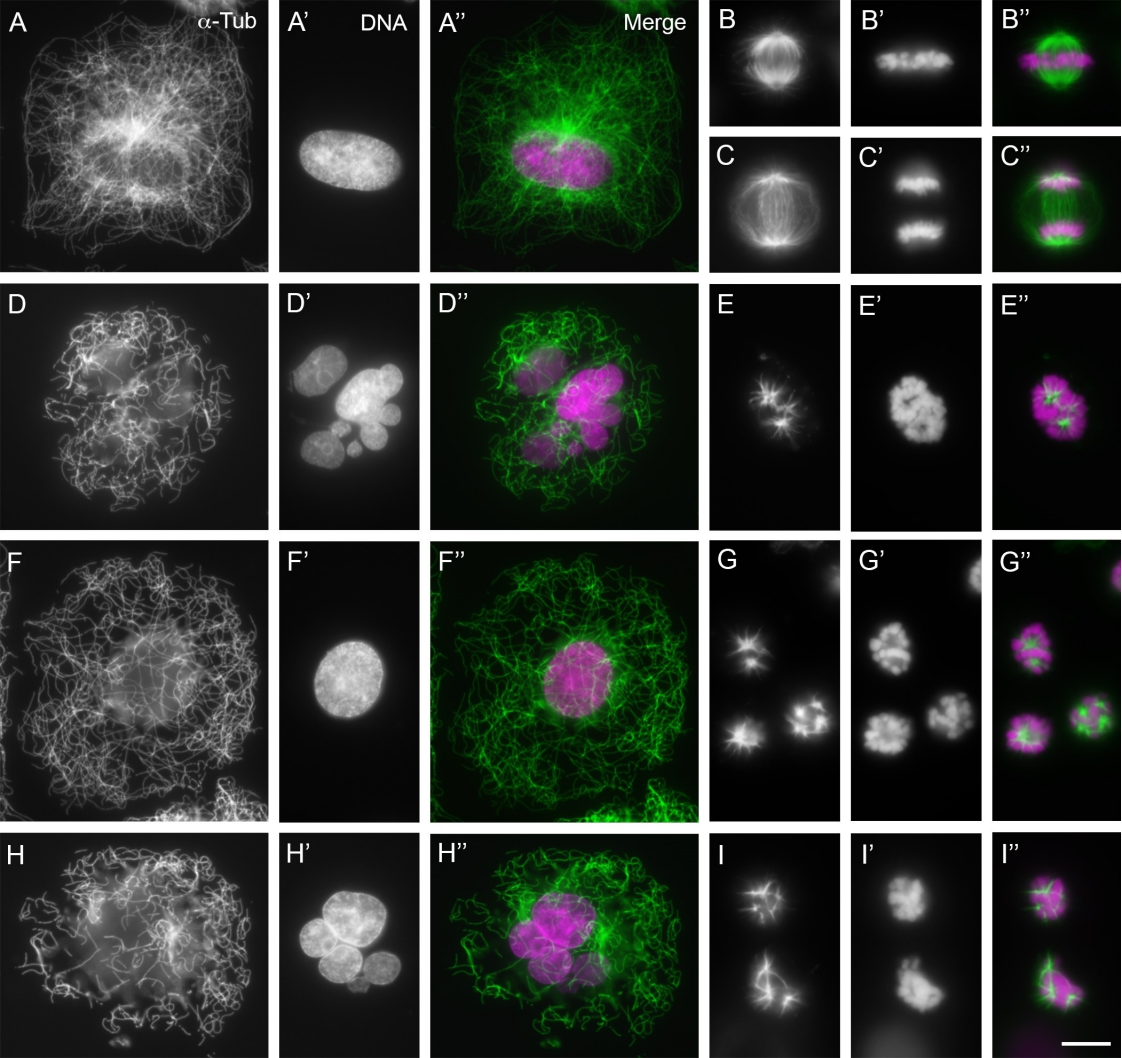
Effects of maytansinoids on cells in interphase and mitosis. The effect of these compounds on the microtubule network and mitotic spindle was characterized in A549 tumor cells by fluorescence microscopy. Cells were treated for 24 h with the different compounds analyzed: A)–C’’) control (DMSO 0.5 %), D)–E’’) **1 a** 5 nM, F)–G’’) **1 b** 100 nM, and H)–I’’) **4 a** 5 nM. Cells were immunostained for A)–I) α‐tubulin or A’)–I’) DNA, and A’’)–I’’) the images obtained were merged (tubulin in green and DNA in magenta). A)–A’’) Interphase cell treated with DMSO; notice the regular microtubule network evenly distributed in the cytoplasm. B)–B’’) Control metaphase cell with a normally distributed bipolar mitotic spindle in which all chromosomes are positioned in the metaphase plate. C)–C’’) Control late‐anaphase cell in which sister chromatids are observed segregating to the daughter poles through a bipolar anaphase spindle; note that no anaphases are later observed in treated cells. D)–D’’, F)–F’’, H)–H’’) Interphase cells displaying a range of less‐dense microtubule networks and reduction of the microtubule mass compared with A–A’’ (multinucleated heteroploid cells ‐D’, H’‐ or single‐nucleated cells ‐F’‐ may appear in any of the treatments with the three compounds). E)–E’’, G)–G’’, I)–I’’) Blocked mitotic cells with condensed chromosomes showing less microtubular mass (when compared with B–B’’) organized in star‐ or comet‐shaped pseudopoles (one cell shown in E–E’’, three in G–G’’ and two in I–I’’). All images are shown at the same scale; scale bar: 10 μm.

In order to finish the characterization of the synthesized maytansinoids, we further investigated the effect of the most potent compound **4 a** on cellular microtubules. To do so, we performed fluorescence microscopy using A549 cells. We incubated cells with increasing concentrations of the ligand for 48 h and compared the effects with those of the reference ligands maytansine, maytansinol and the vehicle (Figure 6).

In interphase the microtubule network covers the whole cytoplasm, while in dividing cells during metaphase there are regular bipolar spindles that allow the correct positioning and segregation of chromosomes in the subsequent division steps. Maytansine (5 nM) effects on interphase (upper panel) and mitotic cells (lower panel). In interphase we can observe a disorganized microtubule network with incipient signs of depolymerization, in an irregular bi‐nucleated cell. Moreover, there are multipolar anomalous spindles in mitosis with DNA starting to condense. Maytansinol require 100 nM concentrations in interphasic cells and 50 nM in mitotic cells to observe the same destabilizing effect seen with maytansine. With **4 a** 10 nM concentrations in interphasic cells and 5 nM in mitotic cells are enough to observe similar effects to those noticed with Maytansine in interphasic cells.

### Determination of T_2_R‐TTL‐maytansinoid structures

In order to validate the above computational analysis and to complement the biological assays, we sought to determine the crystal structures of the respective tubulin–maytansinoid complexes. To this end, we tested the compounds **3**, **4 a**–**c**. Moreover, to evaluate the impact of the C8=C9 double‐bond on the binding pose, we also tested the analogues **5 a**–**c**. Crystals of the T_2_R‐TTL protein complex, containing two ɑ,β‐tubulin dimers, the stathmin‐protein RB3 and the tubulin tyrosine ligase TTL were grown as described by Prota et al.[^31^, ^32^] The above listed compounds were soaked into the crystals over 6 h, which allowed us to solve the T_2_R‐TTL‐maytansinoid structures at high resolution, ranging between 2.25 and 2.7 Å (Table 6). Coordinates and structure factors for all tubulin‐maytansinoid complexes were deposited at the Protein Data Bank (www.rcsb.org), the respective accession numbers are indicated in Table 6. All of the tested ligands were bound to the maytansine site of the β‐tubulin chain in the T_2_R‐TTL complex.

**Table 6:** Overview of resolution of the maytansinoid‐T_2_R‐TTL complex structures and rmsd values in superposition with apo‐T_2_R‐TTL (overall and chain D).

Compound	PDB ID	Resolution [Å]	RMSD overall/chain D (over No. of C_α_ atoms)
**3**	5SB8	2.30	0.25 (1974)/0.163 (380)
**4 a**	5SB9	2.50	0.259 (1958)/0.189 (384)
**4 b**	5SBA	2.25	0.216 (1937)/0.163 (375)
**4 c**	5SBB	2.25	0.232 (1962)/0.153 (369)
**5 a**	5SBC	2.32	0.258 (1946)/0.173 (369)
**5 b**	5SBD	2.25	0.274 (2004)/0.154 (362)
**5 c**	5SBE	2.75	0.266 (1966)/0.197 (394)

The overall T_2_R‐TTL‐maytansinoid structures superimposed very well with the protein structure obtained in the absence of a ligand (PDB ID 4I55; Table 6), thus suggesting that the binding of the maytansinoids has no effect on the overall conformation of tubulin.

**Figure 7:**
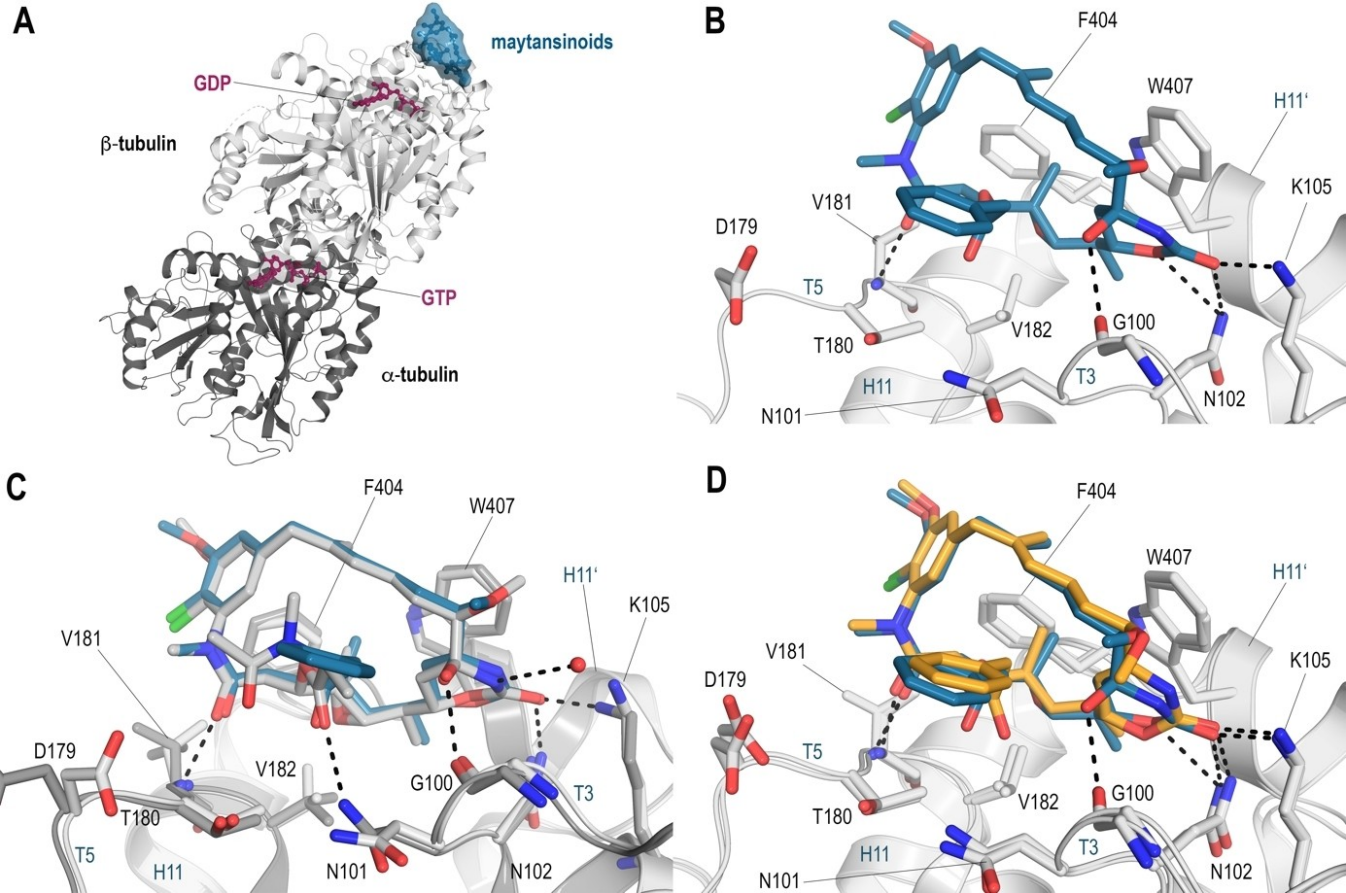
X‐ray analysis of the T_2_R‐TTL‐maytansinoid complexes. A) Overall view of the interaction between the maytansinoids (blue) and tubulin (gray) in relation to the bound nucleotides (purple). The maytansine binding site is located on the β‐tubulin surface in close proximity to the bound GDP molecule. The tubulin molecule is in ribbon representation (gray), and the interacting maytansinoid and nucleotides are in surface and stick representations, respectively. B) Close‐up of the interaction between the maytansinoid **4 a** (PDB ID: 5SB9, blue) and tubulin (gray). The interacting residues and ligand are represented as sticks. Oxygen atoms are in red, nitrogens are blue, and the chlorine atom is in green. Hydrogen bonds are displayed as black dashed lines. C) Superposition of the T_2_R‐TTL‐maytansine structure (PDB ID: 4TV8, maytansine in gray) and the **4 a**‐T_2_R‐TTL structure (**4 a** in blue). The dashed lines indicate the hydrogen‐bond interactions established by the maytansine molecule. **4 a** adopts the same binding pose as its parent compound, except for the interaction of the acyl group with Asn101, which is less pronounced and thus not displayed in the **4 a** structure, all interactions are conserved. D) The superposition of the T_2_R‐TTL‐**4 a** (**4 a** in blue) and the T_2_R‐TTL‐**5 a** (**5 a** in orange) structures shows that the elimination of the C8‐OH group in **5 a** has no major effect on the coordination of the ligand. Although the heterocycle moiety is flattened by the double bond in **5 a**, the position of the ring is anchored at its position by coordination to Asn102 and Lys105.

The binding pose of all the maytansinoids within the maytansine site closely resembles the one described for the parent compound,^[25]^ and all the main interactions are conserved. Briefly, all compounds form hydrogen‐bonds between the C1‐O and the main chain nitrogen atom of Val181 and between the C24‐O and the side chains of Lys105 and Asn102. Additionally, **4 a**–**c** establish a hydrogen bond to the main chain carbonyl group of Gly100 via their C9‐OH group. In Figure 7, we show the binding pose of the best resolved maytansinoid **4 a** and compare it to **5 a**, as well as the maytansine orientation. The detailed binding poses of the other maytansinoids are provided in Figures S23 and S24.

In the studied maytansinoids, all the modifications introduced at the C3 position point towards the solvent and do not perturb the close environment of the maytansine site. For the larger C3 substituents, such as the phenyl ring, we observed a slight reorientation of the C3 carbonyl group, which increases the distance to the Asn101 carbonyl group, thereby weakening this interaction. However, our biological assays showed that this minor change in coordination of the ligand does not have an impact on the efficacy and the binding constants. For the three C3‐modified maytansinoids **4 a**–**c** the determined values are close to the ones of maytansine (Tables 4 and 5). Thus, we can conclude that even attachment of larger groups at this position has no apparent impact on the binding pose, affinity or efficacy.

The structural analysis of the maytansinoids **3**, **5 a**–**c** further reveals that the elimination of the C9‐hydroxy group does not affect the binding mode of the ligands. As shown in Figure 7D, the heterocycle is anchored by two hydrogen bonds established between the C24‐O and the side‐chains of Asn102 and Lys105, which highlights that the interaction is not affected by the introduction of the double bond.

However, the loss of one hydrogen bond between the C9‐OH group and the main chain carbonyl of Gly100 cannot fully account for the observed lower affinities and efficacies of these maytansinoids compared to their hydroxylated analogues, suggesting a contribution by other factors such as compound solubility or decreased stability (Figure 7B).

In order to validate the docking results, we superimposed the crystal structures obtained for each maytansinoid and the best corresponding conformer resulting from the docking studies.

**Figure 8:**
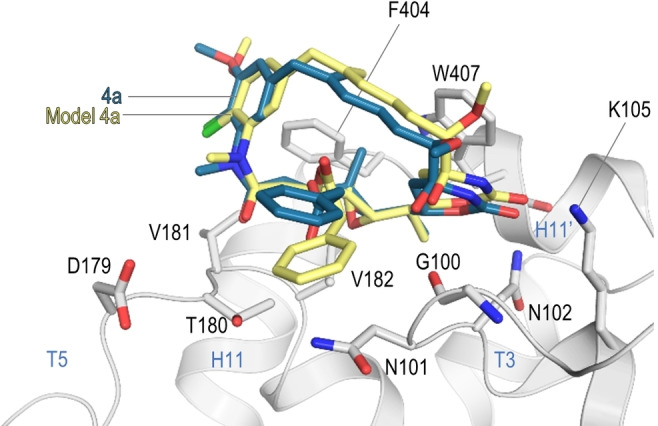
Superimposition of the crystallographic binding mode of compound **4 a** (blue) and the best conformer predicted by AutoDock Vina (light yellow) when bound to β‐tubulin (gray).

In Figure 8 we show the superposition for the **4 a** models; the results for the other maytansinoids can be found in the Supporting Information. For all molecules the docking results agreed well with the models determined by X‐ray crystallography.

These results confirm the reliability of the performed computational docking studies and provides confidence for further usage of the described protocol to investigate novel maytansine‐site binders.

## Conclusion

In summary, we have synthesized a series of maytansinoids containing novel scaffold modifications through extensive investigation of the maytansinol acylation reaction. We were able to shift the reaction towards the specific product formation by screening different reaction conditions. The subtle changes between these structures were carefully analyzed and fully characterized by using NMR spectroscopy. Encouraged by the positive results of our docking studies, we submitted a selection of the obtained compounds for biological evaluation. All of the tested compounds showed distinct effects on tubulin assembly in vitro. Furthermore, the binding affinity of the molecules to tubulin dimers was assessed by the displacement of fluorescent maytansine, with some molecules displaying affinities comparable to maytansine or even higher (**4 a**–**c**). These results correlated well with their potent cytotoxic properties, which were observed in small cell lung carcinoma cells lines (A549, A2780 and A2780AD). We noted that compounds **5 a**–**c**, missing the C9‐OH group, have a significantly lower impact on cells and tubulin in all the performed assays. For C3 esterification, our data prove that acylation (**4 a**–**c**, **5 a**–**c**) is able to reinforce the full biological activity of maytansinoids. In order to elucidate these results further, we determined the structures of tubulin‐compound complexes by X‐ray crystallography. This allowed us to assess the common binding mode of the selected maytansinoids and to validate our docking results, thereby proving that the introduced modifications at positions C9 and C3 do not interfere with binding to the tubulin dimer. However, a structural explanation for the observed differences in biological activity remained elusive. According to the proposed mechanism of action,^[25]^ maytansine poisons the growing ends of MTs by occupying a pocket on β‐tubulin that is essential for the accommodation of helix H8 of the longitudinally aligned α‐tubulin molecules during MT growth. In structural terms, this mechanism should be independent of the modifications at both C9 and C3; this suggests that the differences are likely caused by other factors, such as chemical/metabolic stability, entropy or solubility of the individual compounds. Based on the good agreement of computational and experimental data, we are confident we can proceed with the used computational approaches. This provides us with a solid foundation for future design, synthesis and analysis of next‐generation maytansinoids.

## Conflict of interest

The authors declare no conflict of interest.

## Supporting information

As a service to our authors and readers, this journal provides supporting information supplied by the authors. Such materials are peer reviewed and may be re‐organized for online delivery, but are not copy‐edited or typeset. Technical support issues arising from supporting information (other than missing files) should be addressed to the authors.

Supporting Information
