# Supplementary material for: Maytansinol Derivatives: Side Reactions as a Chance for New Tubulin Binders
Source: Chemistry. 2021 Nov 29;28(2):e202103520. doi: 10.1002/chem.202103520 (PMC9299702; doi:10.1002/chem.202103520)
Supplement: Supplementary file 1 — Supporting Information [file CHEM-28-0-s001.pdf]

Chemistry–A European Journal

Supporting Information

Maytansinol Derivatives: Side Reactions as a Chance for New Tubulin Binders

Paola Marzullo, Zlata Boiarska, Helena Pérez-Peña, Anne-Catherine Abel, Beatriz Álvarez-Bernad, Daniel Lucena-Agell, Francesca Vasile, Maurizio Sironi, Karl-Heinz Altmann, Andrea E. Prota, J. Fernando Díaz, Stefano Pieraccini, and Daniele Passarella*

SUMMARY

Experimental Section S2
General Procedure........ S3
Synthesis of **2** S4
Synthesis of **3** S5
Synthesis of **4a**-**7a** S6
Synthesis of **4b**, **5b** S9
Synthesis of **4c**, **5c** S10
NMR Table Assessment of **1b**, **3**, **4a**-**7a** S12
NMR Spectra S16
Molecular modeling by Docking Analysis S37
Protein and chemicals for biochemistry........ S39
Biochemistry S39
Cell biology S39
Crystallization, Data collection and Structure Determination S40
Supplemental References........ S45

EXPERIMENTAL SECTION

General Experimental Procedures

Unless otherwise stated, reagents were purchased from general suppliers (Sigma Aldrich and Fluorochem) and used without further purification. All solvents were of reagent grade or HPLC grade. All reactions were carried out in oven-dried glassware and dry solvents, under nitrogen atmosphere and were monitored by glasses or aluminium TLC on silica gel (Merck precoated 60F254 plates), with detection by UV light (254 nm) or by TLC stains as permanganate.

Analytical HPLC was performed on Agilent 1100 Series System RP column ZORBAX SB-C8 (3.5μm x 4.6 x 150 mm). The pressure was about 85 bar, with a constant flow rate of 1 mL/min. UV spectra were recorded at 254 nm and 210 nm with DAD detection. The mobile phase consisted of a mixture of H_2O/ACN and the gradient was programmed using the following method: isocratic for 1 min at 50% ACN, then gradient for 10 min to 90% ACN. The system was washed keeping this condition for another 1 min and then bringing back to the beginning condition.

Products were purified by flash column chromatography, using silica gel Merk 60 (230-400 mesh) as stationary phase or by flash purification using Biotage Isolera™ One System and Biotage® Sfär C18 6 g D Duo 30 μm as cartridges (BIOTAGE). The products were eluted from the column with a mixture of H_2O/ACN, running with a gradient from 10% ACN to 95% ACN in 25CV (unless otherwise specified).

^{1}H NMR and ^{13}C-NMR spectra were recorded on a Bruker Avance Spectrometer 400 MHz using commercially available deuterated solvents (chloroform-d, methanol-d4, acetone-d6) at room temperature. Chemical shifts (δ) are reported in parts per million (ppm) and are reported relative to TMS, used as an internal standard. Data for ^{1}H NMR are reported as follows: chemical shift (δ/ppm), multiplicity, coupling constants (Hz). Multiplicities are reported as follows: s = singlet, d = doublet, t = triplet, m = multiplet, br s= broad singlet. Data for 13C NMR are reported in terms of chemical shift (δ/ppm).

High resolution mass spectra (HR-MS) were recorded on a Water QToF Premier high resolution UPLC ES MS/MS.

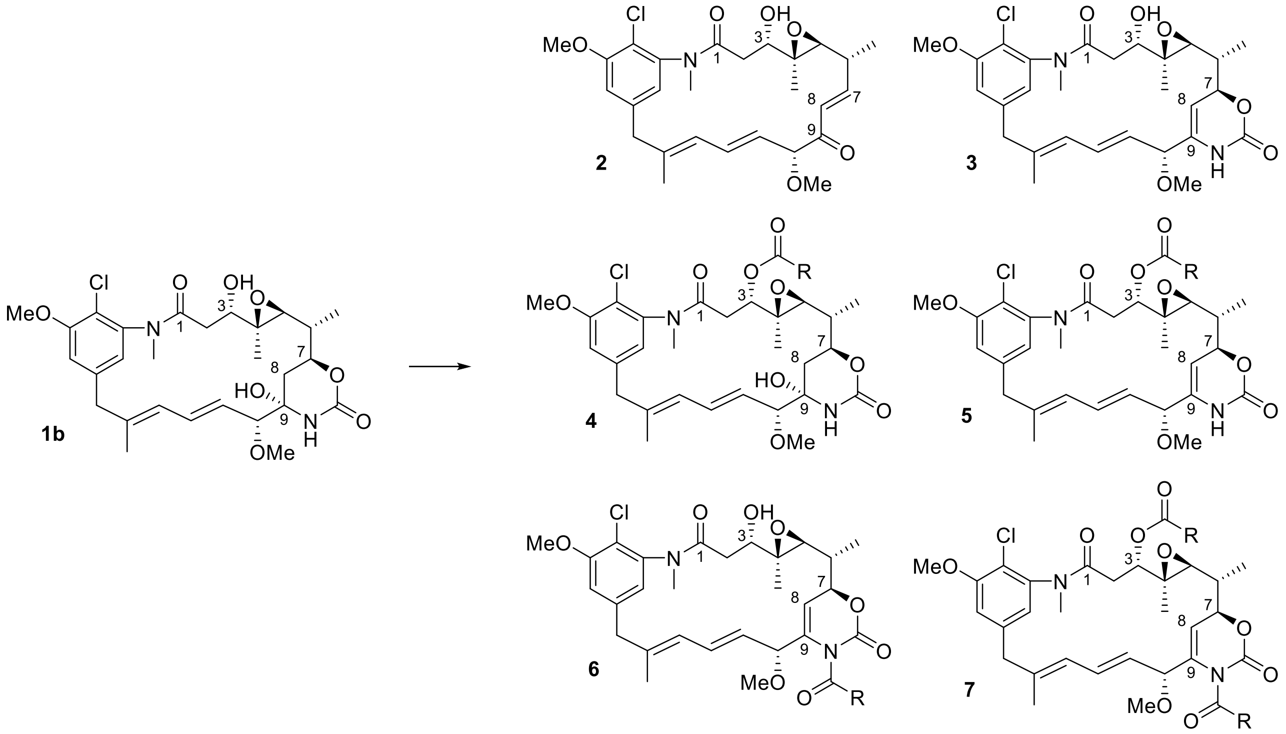

General procedures with acyl chloride agents

To a solution of maytansinol (50 mg, 0.088 mmol) in CH_2Cl_2 (880 µL) were added trimethylamine freshly distilled and 4-(1-pyrrolidinyl)pyridine (6.6 mg, 0.044 mmol) under an atmosphere of nitrogen. The reaction was cooled at 0°C and the acyl chloride was dropwise. The mixture was warmed at room temperature and stirred for a specific time (more details follow below) before quenching with diethylamine. $NaHCO_3$ (1 mL) was added and stirred for 5 min. The reaction was extracted with CH_2Cl_2 (3 × 2 mL) and the combined organic layers were dried over Na_2SO_4 and concentrated under reduced pressure. Column chromatography of the residue on silica gel (elution: CH_2Cl_2/MeOH 96:4) provided the products.

General procedures with coupling agents

To a solution of maytansinol (50 mg, 0.088 mmol) in dry CH_2Cl_2 (440 µL) were added DMAP and the carboxylic acid at room temperature under a nitrogen atmosphere. Then, a solution 1.3 M of DCC in dry CH_2Cl_2 was slowly added (or were added EDC-HCl followed by dropwise trimethylamine freshly distilled). The mixture was stirred at room temperature for a specific time (more details follow below) before filtering off the DCU using cold CH_2Cl_2 (or quenching with an aqueous saturated solution of NH_4Cl, in the case of EDC). The organic phase was washed with H_2O (4 × 1 mL), with brine (1 × 1 mL), then dried over Na_2SO_4, and concentrated under reduced pressure. Column chromatography of the residue on silica gel (elution: CH_2Cl_2/MeOH 96:4), or purification with Biotage Isolera™ One System, provided the products as white powders.

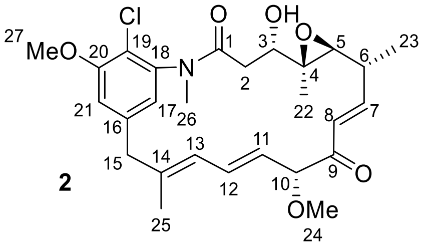

2

To a solution of maytansinol (25 mg, 0.044 mmol) in MeCN/DMF 10:1 (290 µL) was added Cs_2CO_3 (36 mg, 0.111 mmol), TEBA (0.2 mg, 0.001 mmol), KI (14.7 mg, 0.088 mmol), and then was dropped propargyl bromide (80% in Tol, 8 µL, 0.088 mmol). The reaction was stirred for 2.5 h before to quench with a saturated aqueous solution of NH_4Cl and to extract with EtOAc (× 3). The combined organic layers were washed with a saturated aqueous solution of NaCl, dried over Na_2SO_4, and concentrated under reduced pressure. Column chromatography of the residue on silica gel (elution: CH_2Cl_2/MeOH 97:3) provided 8.5 mg (35%) of the product.

2 - ^{1}H NMR (400 MHz, methanol-*d*4) δ 7.12 (d, *J* = 1.9 Hz, 1H, 17), 7.09 (d, *J* = 2.0 Hz, 1H, 21), 6.87 – 6.68 (m, 2H, 7, 12), 6.42 (d, *J* = 15.6 Hz, 1H, 8), 6.12 (dd, *J* = 13.3, 5.5 Hz, 1H, 13), 5.31 (dd, *J* = 15.2, 8.7 Hz, 1H, 11), 4.60 (d, *J* = 8.8 Hz, 1H, 10), 3.99 (s, 3H, 27), 3.50 (d, *J* = 12.8 Hz, 1H, 15'), 3.38 (t, *J* = 2.7 Hz, 1H, 3), 3.35 (s, 3H, 24), 3.27 (d, *J* = 12.8 Hz, 1H, 15''), 3.18 (s,34H, 26), 2.67 (d, *J* = 9.1 Hz, 1H, 5), 2.34 – 2.24 (m, 1H, 2'), 2.24 – 2.17 (m, 1H, 6), 2.03 (dd, *J* = 13.0, 2.9 Hz, 1H, 2''), 1.79 (s, 3H, 25), 1.22 (d, *J* = 6.6 Hz, 3H, 23), 0.80 (s, 3H, 22).

2 - ^{13}C NMR (101 MHz, methanol-*d*4) δ 196.0 (9), 172.2 (1), 155.8 (20), 146.3 (7), 142.0 (18), 140.6 (16), 139.6 (14), 133.3 (12), 127.2 (8), 126.0 (11), 125.1 (13), 123.3 (17), 118.0 (19), 113.0 (21), 87.5 (10), 73.5 (3), 66.2 (5), 62.4 (4), 55.7 (27), 55.4 (24), 45.9 (15), 37.3 (6), 35.0 (2), 34.8 (26), 16.1 (25), 14.8 (23), 10.0 (22).

2 - HRMS (ESI) *m/z* $[M+Na]^+$ 526.1974 (calcd for $C_{21}H_{34}ClNO_2Na$, 526.1972)

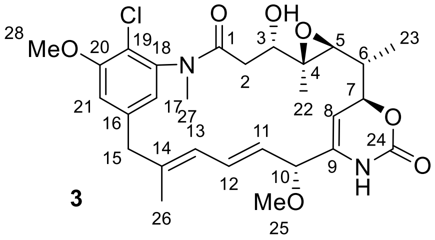

3

Reaction was performed as described in **Method with acyl chloride agent**: This reaction was completely performed at -20°C; maytansinol (100 mg, 0.177 mmol); trimethylamine (49 µL, 0.354 mmol); 4-(1-pyrrolidinyl)pyridine (26.2 mg, 0.177 mmol); benzoylchloride (10 µL, 0.088 mmol); reaction time 30 min; diethylamine (27 µL, 0.265 mmol); **3** (5.4 mg, 5%); recovered starting material (82.3 mg).

Reaction was performed as described in **Method with acyl chloride agent**: This reaction was completely performed at -20°C; maytansinol (50 mg, 0.088 mmol); trimethylamine (25 µL, 0.177 mmol); 4-(1-pyrrolidinyl)pyridine (6.6 mg, 0.044 mmol); benzoylchloride (10 µL, 0.088 mmol); reaction time 2 h; diethylamine (27 µL, 0.265 mmol); **3** (7.6 mg, 15%); recovered starting material (82.3 mg).

3 - ^{1}H NMR (400 MHz, chloroform-*d*) δ 7.08 (s, 1H, NH), 6.97 (d, *J* = 1.9 Hz, 1H, 17), 6.83 (d, *J* = 1.9 Hz, 1H, 21), 6.46 (dd, *J* = 15.2, 10.8 Hz, 1H, 12), 6.16 (d, *J* = 11.1 Hz, 1H, 13), 5.54 (dd, *J* = 15.2, 8.5 Hz, 1H, 11), 5.02 – 4.96 (m, 1H, 8), 4.78 (dd, *J* = 10.5, 2.3 Hz, 1H, 7), 4.20 – 4.12 (m, 1H, 10), 4.00 (s, 3H, 28), 3.59 (dd, *J* = 10.9, 2.4 Hz, 1H, 3), 3.49 (d, *J* = 12.8 Hz, 1H, 15'), 3.35 (s, 3H, 25), 3.23 (s, 3H, 27), 3.15 (d, *J* = 12.9 Hz, 1H, 15''), 2.55 (d, *J* = 9.7 Hz, 1H, 5), 2.32 (dd, *J* = 13.6, 10.8 Hz, 1H, 2''), 2.14 (dd, *J* = 13.7, 2.3 Hz, 1H, 2'), 1.75 (s, 3H, 26), 1.66 (td, *J* = 10.1, 6.4 Hz, 1H, 6), 1.27 (d, *J* = 2.3 Hz, 3H, 23), 0.84 (s, 3H, 22).

3 - ^{13}C NMR (101 MHz, chloroform-*d*) δ 171.6 (1), 156.1 (20), 151.5 (24), 143.0 (18), 140.4 (16), 140.3 (14), 136.1 (9), 133.5 (12), 126.9 (11), 124.7 (13), 123.3 (17), 119.0 (19), 112.5 (21), 99.1 (8), 80.1 (7), 80.0 (10), 75.9 (3), 66.2 (5), 62.4 (4), 56.7 (28), 56.6 (25), 46.8 (15), 39.0 (6), 36.0 (27), 35.7 (2), 16.4 (26), 14.6 (23), 11.2 (22).

3 - HRMS (ESI) *m/z* $[M+Na]^+$ 569.2035 (calcd for $C_{28}H_{35}ClN_2O_7Na$, 569.2030)

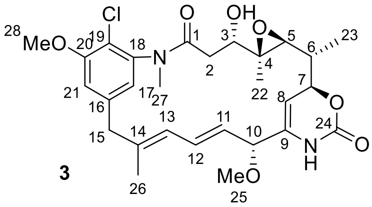

3

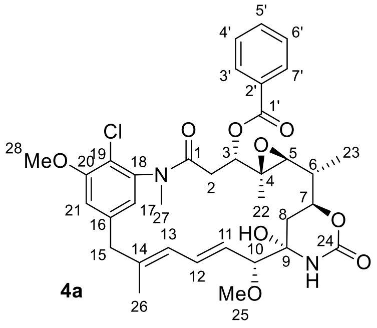

4a

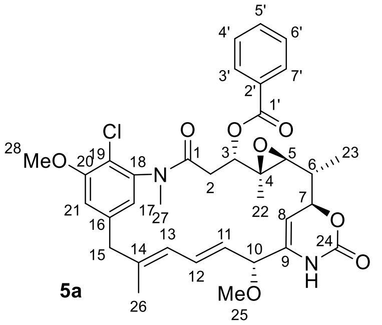

5a

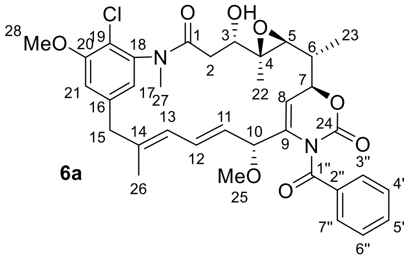

6a

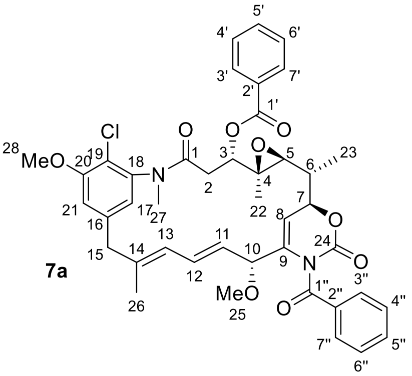

7a

Reaction was performed as described in **Method with acyl chloride agent**: trimethylamine (25 µL, 0.177 mmol); benzoylchloride (10 µL, 0.088 mmol); reaction time 6 h; diethylamine (10 µL, 0.097 mmol); column chromatography on silica gel (elution gradient: Hex/EtOAc 1:1 → EtOAc 1 → EtOAc/MeOH 95:5); **3a+4a** 1:1 (12 mg, 22%).

To a solution of maytansinol (38 mg, 0.067 mmol) in pyridine (670 µL) at 0°C was added dropwise benzoylchloride (47 µL, 0.402 mmol) under an atmosphere of nitrogen. The mixture was warmed at room temperature and stirred for 5 h. Water (1 mL) was added and the aqueous phase was extracted with EtOAc (3 × 2 mL). The combined organic layers were washed with NaCl (1 mL), dried over Na_2SO_4 and concentrated under reduced pressure. The crude was dissolved in heptane and concentrated several times to co-evaporate the excess of pyridine. Column chromatography of the residue on silica gel (elution gradient: CH_2Cl_2 1 → CH_2Cl_2/MeOH 96:4) provided 11 mg (25%) of **4a**.

Reaction was performed as described in **Method with acyl chloride agent**: trimethylamine (123 µL, 0.885 mmol); benzoylchloride (41 µL, 0.354 mmol); reaction time 4 h; diethylamine (40 µL, 0.387 mmol); column chromatography on silica gel (elution gradient: Hex/EtOAc 1:1 → EtOAc 1 → EtOAc/MeOH 95:5); **6a** (19 mg, 33%); **7a** (45 mg, 67%).

Reaction was performed as described in **Method with coupling agent**: DMAP (32 mg, 0.26 mmol); benzoic acid (32 mg, 0.26 mmol); DCC (60 mg, 0.29 mmol); reaction time 8 h; **3** (2.3 mg, 5%); **4a** (20.4 mg, 35%); **5a** (14.7 mg, 26%); **7a** (12.6 mg, 18%).

Reaction was performed as described in **Method with coupling agent**: DMAP (65 mg, 0.53 mmol); benzoic acid (65 mg, 0.53 mmol); DCC (120 mg, 0.58 mmol); reaction time 48 h; **5a** (8.6 mg, 15%); **7a** (19.1 mg, 31%).

Reaction was performed as described in **Method with coupling agent**: maytansinol (20 mg, 0.035 mmol); DMAP (13 mg, 0.11 mmol); benzoic acid (13 mg, 0.11 mmol); EDC-HCl (22 mg, 0.12 mmol); TEA (17 µl, 0.13 mmol); reaction time 48 h; **4a** (3.5 mg, 15%); **5a** (10.7 mg, 47%); **7a** (5.5 mg, 20%).

Reaction was performed as described in **Method with coupling agent**: maytansinol (20 mg, 0.035 mmol); DMAP (13 mg, 0.11 mmol); benzoic acid (13 mg, 0.11 mmol); EDC-HCl (22 mg, 0.12 mmol); TEA (17 µl, 0.13 mmol); reaction time 24 h; **3** (1.5 mg, 8%); **4a** (9.1 mg, 39%); **5a** (2.7 mg, 12%); **7a** (1.8 mg, 7%).

To a solution of DMAP (60 mg, 0.5 mmol) in THF (250 µL) was added a solution of p-TSA (95 mg, 0.5 mmol) in THF (250 µL). The reaction was stirred for 40 minutes at rt before to isolate the preciptate by filtration over a Buchner. The dried product **DPTS** was used in the next step without further purification. To a solution of maytansinol (50 mg, 0.088 mmol) in dry CH_2Cl_2 (440 µL) was added benzoic acid (11 mg, 0.088 mmol) and **DPTS** (13 mg, 0.044 mmol) under an atmosphere of nitrogen. Then, a solution 0.5 M of DCC in dry CH_2Cl_2 (24 mg, 0.115 mmol) was added dropwise. The mixture was stirred at room temperature for 5 d. Afterwards, the solution was filtered, dried over Na_2SO_4, concentrated under reduced pressure, and analyzed by HPLC. Maytansinol (3.10 min, Area 55%); **3** (4.08 min, Area 4%); **4a** (5.20 min, Area 18%); **5a** (6.90 min, Area 14%); **6a** (7.20 min, Area 5%); **7a** (9.40 min, Area 4%).

To a solution of maytansinol (50 mg, 0.088 mmol) in dry CH_2Cl_2 (440 µL) was added two drops of DMF dry, DMAP (33 mg, 0.26 mmol), benzoic acid (33 mg, 0.26 mmol), and $ZnCl_2$ (36 mg, 0.27 mmol) under an atmosphere of nitrogen. Then, a solution 1.3 M of DCC in dry CH_2Cl_2 (60 mg, 0.29 mmol) was added dropwise. To promote $ZnCl_2$ dissolution, a minimal quantity of DMF was added. The mixture was stirred at room temperature for 48 h. Afterwards, the solution was filtered, dried over Na_2SO_4, concentrated under reduced pressure, and analyzed by HPLC. Maytansinol (3.10 min, Area 29%); **4a** (5.20 min, Area 62%); **5a** (6.90 min, Area 4%); **6a** (7.20 min, Area 4%).

4a - ^{1}H NMR (400 MHz, acetone-*d*$_6$) δ 8.21 – 8.08 (m, 2H, 3', 7'), 7.77 – 7.59 (m, 3H, 4', 5', 6'), 7.27 (d, *J* = 1.9 Hz, 1H, 21), 7.19 (d, *J* = 1.8 Hz, 1H, 17), 6.61 (dd, *J* = 15.5, 11.1 Hz, 1H, 12), 6.33 (s, 1H, NH), 6.04 (d, *J* = 11.0 Hz, 1H, 13), 5.04 – 4.85 (m, 2H, 3, 11), 4.47 (s, 1H, OH), 4.31 – 4.14 (m, 1H, 7), 4.04 (s, 3H, 28), 3.59 (d, *J* = 12.6 Hz, 1H, 15a), 3.50 (d, *J* = 9.1 Hz, 1H, 10), 3.36 (d, *J* = 12.7 Hz, 1H, 15b), 3.24 (s, 3H, 25), 3.13 (s, 3H, 27), 3.05 (d, *J* = 9.5 Hz, 1H, 5), 2.88 – 2.79 (m, 1H, 2a), 2.26 (dd, *J* = 14.4, 3.1 Hz, 1H, 2b), 1.77 (s, 3H, 26), 1.59 (dt, *J* = 13.6, 1.9 Hz, 1H, 8b), 1.54 – 1.39 (m, 2H, 6, 8a), 1.24 (d, *J* = 6.4 Hz, 3H, 23), 0.97 (s, 3H, 22).

4a - ^{13}C NMR (101 MHz, acetone-*d*$_6$) δ 168.0 (1), 165.9 (1'), 156.2 (20), 151.1 (24), 142.4 (18), 141.3 (16), 139.6 (14), 133.1 (5'), 132.4 (12), 130.5 (2'), 129.9 (3', 7'), 128.6 (11), 128.2 (4', 6'), 124.7 (13), 122.1 (17), 118.7 (19), 113.5 (21), 88.9 (10), 80.5 (9), 77.5 (3), 73.8 (7), 66.4 (5), 60.5 (4), 56.2 (28), 55.8 (25), 46.3 (15), 38.7 (6), 36.2 (8), 34.6 (27), 32.7 (2), 14.9 (26), 14.0 (23), 12.1 (22).

4a - HRMS (ESI) *m/z* $[M+Na]^+$ 691.2396 (calcd for $C_{35}H_{41}ClN_2O_9Na$, 691.2398)

5a - ^{1}H NMR (400 MHz, acetone-*d*$_6$) δ 8.19 (dd, *J* = 8.2, 1.6 Hz, 2H, 3', 7'), 8.03 (s, 1H, NH), 7.86 – 7.69 (m, 3H, 4', 5', 6'), 7.28 (d, *J* = 1.8 Hz, 1H, 21), 7.17 (d, *J* = 1.8 Hz, 1H, 17), 6.54 (dd, *J* = 15.3, 10.9 Hz, 1H, 12), 6.13 (d, *J* = 11.0 Hz, 1H, 13), 5.02 (dd, *J* = 11.9, 3.0 Hz, 1H, 3), 4.88 (dd, *J* = 15.3, 8.4 Hz, 1H, 11), 4.56 (dd, *J* = 10.8, 3.4 Hz, 1H, 7), 4.43 (dt, *J* =

3.2, 1.5 Hz, 1H, 8), 4.15 (d, *J* = 8.5 Hz, 1H, 10), 4.04 (s, 3H, 28), 3.66 (d, *J* = 12.7 Hz, 1H, 15a), 3.41 (d, *J* = 12.6 Hz, 1H, 15b), 3.15 (s, 3H, 25), 3.14 (s, 3H, 27), 3.03 (d, *J* = 9.6 Hz, 1H, 5), 2.91 (dd, *J* = 14.6, 12.0 Hz, 1H, 2b), 2.22 (dd, *J* = 14.6, 3.0 Hz, 1H, 2a), 1.79 (s, 3H, 26), 1.65 (ddd, *J* = 10.6, 9.7, 6.5 Hz, 1H, 6), 1.19 (d, *J* = 6.5 Hz, 3H, 23), 0.90 (s, 3H, 22).

5a - ^{13}C NMR (101 MHz, acetone-d_6) ^{13}C NMR (101 MHz,) δ 168.0 (1), 165.7 (1'), 156.2 (20), 149.9 (24), 142.3 (18), 141.2 (16), 140.7 (14), 137.0 (9), 133.7 (5'), 133.0 (12), 130.2 (2'), 129.5 (3', 7'), 128.9 (4', 6'), 126.7 (11), 124.0 (13), 122.2 (17), 118.7 (19), 113.4 (21), 98.0 (8), 79.5 (10), 78.2 (7), 77.9 (3), 67.0 (5), 60.2 (4), 56.2 (28), 55.4 (25), 46.2 (15), 39.5 (6), 34.7 (27), 32.2 (2), 15.1 (26), 13.5 (23), 11.7 (22).

5a - HRMS (ESI) *m/z* $[M+Na]^+$ 673.2295 (calcd for $C_{35}H_{39}ClN_2O_8Na$, 673.2293)

6a - ^{1}H NMR (400 MHz, chloroform-*d*) δ 7.83 (d, *J* = 7.6 Hz, 2H, 3'', 7''), 7.54 (t, *J* = 7.2 Hz, 1H, 5''), 7.46 (t, *J* = 7.5 Hz, 2H, 4'', 6''), 7.01 (d, *J* = 1.8 Hz, 1H, 17), 6.82 (d, *J* = 1.8 Hz, 1H, 21), 6.48 (dd, *J* = 15.2, 10.8 Hz, 1H, 12), 6.15 (d, *J* = 10.8 Hz, 1H, 13), 5.54 (dd, *J* = 15.2, 8.4 Hz, 1H, 11), 5.43 – 5.33 (m, 1H, 8), 4.86 (d, *J* = 10.8 Hz, 1H, 7), 4.42 (d, *J* = 8.4 Hz, 1H, 10), 3.99 (s, 3H, 28), 3.61 (d, *J* = 9.7 Hz, 1H, 3), 3.56 – 3.42 (m, 1H, 15a), 3.23 (s, 3H, 27), 3.12 (d, *J* = 13.0 Hz, 1H, 15b), 2.82 (s, 3H, 25), 2.59 (d, *J* = 9.6 Hz, 1H, 5), 2.37 (dd, *J* = 13.7, 10.9 Hz, 1H, 2a), 2.12 (d, *J* = 13.6 Hz, 1H, 2b), 1.76 (s, 3H, 26), 1.68 (q, *J* = 4.9, 4.5 Hz, 1H, 6), 1.32 – 1.20 (m, 3H, 23), 0.85 (s, 3H, 22).

6a - ^{13}C NMR (101 MHz, $CDCl_3$) δ 172.2 (1), 170.2 (1''), 156.5 (20), 149.7 (24), 143.0 (18), 141.0 (16), 140.7 (14), 137.7 (9), 134.8 (2''), 134.4 (12), 132.7 (5''), 129.3 (4'', 6''), 128.1 (3'', 7''), 127.1 (11), 125.4 (13), 123.9 (17), 119.4 (19), 112.9 (21), 103.1 (8), 80.4 (7), 78.5 (10), 76.5 (3), 67.1 (5), 63.6 (4), 57.2 (28), 56.2 (25), 47.3 (15), 40.6 (6), 36.6 (27), 36.4 (2), 16.9 (26), 14.9 (23), 11.6 (22).

6a - HRMS (ESI) *m/z* $[M+Na]^+$ 673.2296 (calcd for $C_{35}H_{39}ClN_2O_8Na$, 673.2293)

7a - ^{1}H NMR (400 MHz, acetone-d_6) δ 8.22 (d, *J* = 7.4 Hz, 2H, 3', 7'), 7.89 (d, *J* = 7.7 Hz, 2H, 3'', 7''), 7.78 (q, *J* = 7.4, 6.4 Hz, 3H, 4', 5', 6'), 7.65 (q, *J* = 5.1 Hz, 1H, 5''), 7.53 (dd, *J* = 7.9, 4.8 Hz, 2H, 4'', 6''), 7.30 (s, 1H, 21), 7.19 (s, 1H, 17), 6.64 (dd, *J* = 15.3, 11.1 Hz, 1H, 12), 6.12 (d, *J* = 11.0 Hz, 1H, 13), 5.04 (d, *J* = 11.6 Hz, 1H, 3), 4.92 – 4.76 (m, 3H, 7, 8, 11), 4.35 (d, *J* = 8.2 Hz, 1H, 10), 4.05 (s, 3H, 28), 3.64 (d, *J* = 12.5 Hz, 1H, 15a), 3.41 (d, *J* = 12.6 Hz, 1H, 15b), 3.17 (s, 3H, 27), 3.12 (d, *J* = 9.2 Hz, 1H, 5), 2.99 – 2.87 (m, 1H, 2a), 2.68 (s, 3H, 25), 2.27 (d, *J* = 14.2 Hz, 1H, 2b), 1.83 (s, 3H, 26), 1.75 (dq, *J* = 8.3, 5.0, 4.5 Hz, 1H, 6), 1.24 (d, *J* = 6.4 Hz, 3H, 23), 0.97 (s, 3H, 22).

7a - ^{13}C NMR (101 MHz, acetone-d_6) δ 168.0 (1), 166.8 (1''), 165.8 (1'), 156.3 (20), 148.3 (24), 142.3 (18), 141.6 (14), 141.1 (16), 137.3 (9), 134.6 (2''), 134.5 (12), 133.8 (5'), 133.3 (5''), 130.2 (2'), 129.7 (3', 7'), 129.5 (3'', 7''), 129.0 (4', 6'), 128.4 (4'', 6''), 125.8 (11), 123.8 (13), 122.2 (17), 118.7 (19), 113.4 (21), 102.1 (8), 78.4 (7), 78.2 (3), 77.6 (10), 66.9 (5), 60.2 (4), 56.2 (28), 54.6 (25), 46.2 (15), 40.4 (6), 34.7 (27), 32.3 (2), 15.2 (26), 13.5 (23), 11.6 (22).

7a - HRMS (ESI) *m/z* $[M+Na]^+$ 777.2559 (calcd for $C_{42}H_{43}ClN_2O_9Na$, 777.2555)

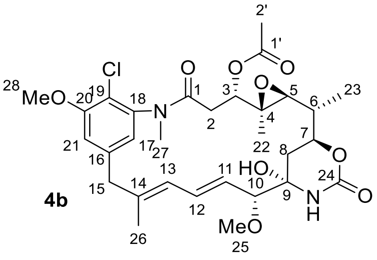

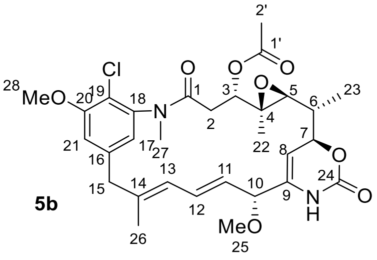

Reaction was performed as described in **Method with acyl chloride agent**: trimethylamine (31 µL, 0.221 mmol); acetylchloride (13 µL, 0.177 mmol); reaction time 6 h; diethylamine (20 µL, 0.193 mmol); column chromatography on silica gel (elution: CH_2Cl_2/MeOH 96:4); **4b** (25 mg, 46%).

Reaction was performed as described in **Method with coupling agent**: DMAP (32 mg, 0.26 mmol,); acetic acid (15 µl, 0.260 mmol); DCC (60 mg, 0.29 mmol); reaction time 4 h; **4b** (22.2 mg, 42%), **5b** (22.4 mg, 43%).

4b - ^{1}H NMR (400 MHz, acetone-*d6*) δ 7.25 (d, *J* = 1.9 Hz, 1H, 21), 6.97 (d, *J* = 1.8 Hz, 1H, 17), 6.75 (dd, *J* = 15.5, 11.1 Hz, 1H, 12), 6.42 (s, 1H, NH), 6.38 (d, *J* = 11.1 Hz, 1H, 13), 5.65 (dd, *J* = 15.4, 9.0 Hz, 1H, 11), 4.99 (s, 1H, OH), 4.83 (dd, *J* = 11.9, 2.7 Hz, 1H, 3), 4.20 (ddd, *J* = 12.5, 10.5, 2.3 Hz, 1H, 7), 4.02 (s, 3H, 28), 3.72 – 3.60 (m, 2H, 10, 15a), 3.37 (d, *J* = 2.3 Hz, 4H, 15b, 25), 3.12 (s, 3H, 27), 2.75 (d, *J* = 9.8 Hz, 1H, 5), 2.60 (dd, *J* = 13.8, 11.9 Hz, 1H, 2a), 2.22 (s, 3H, 2'), 2.07 – 1.98 (m, 1H, 2b), 1.77 (s, 3H, 26), 1.72 – 1.62 (m, 1H, 8a), 1.62 – 1.43 (m, 2H, 6, 8b), 1.21 (d, *J* = 6.3 Hz, 3H, 23), 0.95 (s, 3H, 22).

4b - ^{13}C NMR (101 MHz, acetone-*d6*) δ 169.7 (1'), 168.8 (1), 156.6 (20), 151.7 (24), 143.0 (18), 141.8 (16), 140.2 (14), 133.0 (12), 129.5 (11), 125.6 (13), 123.1 (17), 119.2 (19), 114.3 (21), 89.1 (10), 81.5 (9), 77.4 (3), 74.5 (7), 66.8 (5), 61.2 (4), 56.7 (28), 56.5 (25), 46.9 (15), 38.7 (6), 36.7 (8), 35.3 (27), 33.2 (2), 20.9 (2'), 15.5 (26), 14.5, 12.1 (22).

4b - HRMS (ESI) *m/z* $[M+Na]^+$ 629.2244 (calcd for $C_{30}H_{39}ClN_2O_9Na$, 629.2242)

5b - ^{1}H NMR (400 MHz, acetone-*d6*) δ 8.09 (s, 1H, NH), 7.24 (d, *J* = 1.8 Hz, 1H, 21), 7.02 (d, *J* = 1.7 Hz, 1H, 17), 6.67 (dd, *J* = 15.2, 11.2 Hz, 1H, 12), 6.36 (d, *J* = 11.1 Hz, 1H, 13), 5.75 – 5.54 (m, 1H, 11), 4.69 (dd, *J* = 11.8, 2.7 Hz, 1H, 3), 4.64 – 4.49 (m, 2H, 7, 8), 4.29 (d, *J* = 8.3 Hz, 1H, 10), 4.02 (s, 3H, 28), 3.64 (d, *J* = 12.7 Hz, 1H, 15a), 3.38 (d, *J* = 12.7 Hz, 1H, 15b), 3.31 (s, 3H, 25), 3.15 (s, 3H, 27), 2.81 (d, *J* = 9.7 Hz, 1H, 5), 2.65 (dd, *J* = 13.9, 11.8 Hz, 1H, 2a), 2.31 (s, 3H, 2'), 2.07 (m, 1H, 2b), 1.80 (s, 3H, 26), 1.69 – 1.53 (m, 2H, 6), 1.17 (d, *J* = 6.5 Hz, 3H, 23), 0.86 (s, 3H, 22).

5b - ^{13}C NMR (101 MHz, acetone-*d6*) ^{13}C NMR (101 MHz, Acetone) δ 169.3 (1'), 168.3 (1), 155.9 (20), 142.3 (18), 140.9 (16), 140.6 (14), 137.3 (24), 133.1 (12), 127.0 (11), 124.1 (13), 122.7 (17), 118.5 (19), 113.4 (21), 97.7 (8), 79.5 (10), 78.5 (7), 77.7 (3), 75.1, 67.1 (5), 60.2 (4), 56.1 (28), 56.1, 55.8 (25), 46.1 (15), 39.4 (6), 34.8 (27), 32.4 (2), 20.5 (2'), 15.1 (26), 13.5 (23), 11.0 (22). Signal of 9 is covered

5b - HRMS (ESI) *m/z* $[M+Na]^+$ 611.2141 (calcd for $C_{30}H_{37}ClN_2O_8Na$, 611.2136)

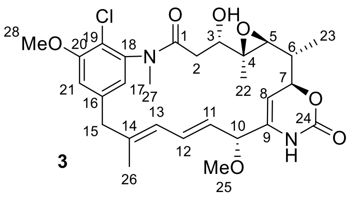

3

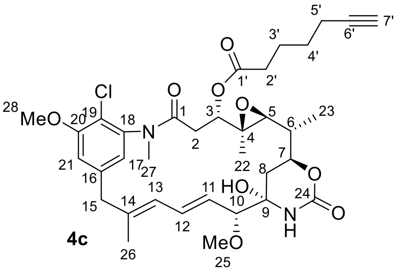

4c

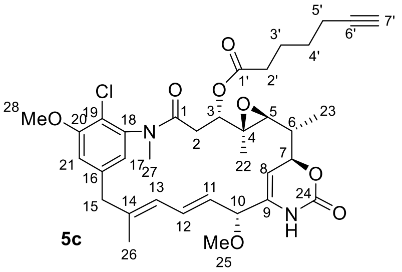

5c

4c prepared in collaboration with Altmann's group at ETH Zurich using the same procedure described in the article.[1]

Maytansinol (33 mg, 0.058 mmol) was lyophilized from 1,4-dioxane overnight and then dissolved in CH_2Cl_2 (580 µL). To the solution was added triethylamine (81 µL, 0.58 mmol) followed by 4-(1-pyrrolidinyl)pyridine (4.3 mg, 0.029 mmol) under argon atmosphere. Then, 6-heptynoyl chloride (35 µl, 0.23 mmol) (prepared by heating 6-heptynoic acid with a 7-fold excess of oxalyl chloride in dry CH_2Cl_2 to 70°C for 1.5 h) was added and the reaction was stirred at rt for 5 d. Additional triethylamine (81 µL, 0.55 mmol), 4-(1-pyrrolidinyl)pyridine (4.3 mg, 0.029 mmol) and 6-heptynoyl chloride (35 µl, 0.23 mmol) were supplied. The stirring was continued for another two days. The mixture was evaporated and the crude was purified by preparative RP-HPLC, which was carried out on a Gilson system using a Waters Symmetry column (5 µM, 19x100 mm) with acetonitrile (ACN) and water as eluent solvents: wavelength detection 254 nm; scanning 200-600 nm; flow rate 25.0 mL/min; isocratic at 40% ACN for 1 min, then to 70% ACN in 14 min, then the system was washed at 95% ACN for 6 min to restore then the beginning conditions. **4c** was isolated in 6.1 mg (16%) as white solid, and **5c** was isolated in 1.3 mg (7%).

The reaction was monitored by HPLC and LC-MS to confirm the formation of the desired product. Analytical RP-HPLC was performed on a Hitachi EliteChrom system equipped with a diode array detector, using a Waters Symmetry C18 column (3.5 µM, 4.6x100 mm), and acetonitrile (ACN) and water as eluent solvents: wavelength detection 254 nm; flow rate 1 mL/min; isocratic at 30% ACN for 1 min, then to 70% ACN in 10 min, then the system was washed at 95% ACN for 6 min to restore then the beginning conditions; maytansinol (4.36 min), **4c** (7.67 min).

Reaction was performed as described in **Method with coupling agent**: DMAP (32 mg, 0.26 mmol); 6-heptinoic acid (33 µl, 0.26 mmol); DCC (60 mg, 0.29 mmol) in CH_2Cl_2 (220 µl); reaction time 3 h; **3** (4.5 mg, 9%), **4c** (22 mg, 37%), **5c** (17.5 mg, 30%).

Reaction was performed as described in **Method with coupling agent**: DMAP (32 mg, 0.26 mmol); 6-heptynoic acid (32, 0.26 mmol); EDC-HCl (56 mg, 0.29 mmol); trimethylamine (40 µl, 0.29 mmol); reaction time 18 h; **4c** (18.4 mg, 36%), **5c** (7.4 mg, 15%).

4c - ^{1}H NMR (400 MHz, chloroform-*d*) δ 6.83 (d, *J* = 2.0 Hz, 1H, 21), 6.79 (d, *J* = 1.8 Hz, 1H, 17), 6.44 (dd, *J* = 15.5, 11.0 Hz, 1H, 12), 6.31 (s, 1H, NH), 6.16 (d, *J* = 10.9 Hz, 1H, 13), 5.49 (dd, *J* = 15.5, 8.9 Hz, 1H, 11), 4.89 (dd, *J* = 11.9, 3.0 Hz, 1H, 3), 4.25 (ddd, *J* = 12.4, 10.6, 2.0 Hz, 1H, 7), 3.99 (s, 3H, 28), 3.56 – 3.46 (m, 2H, 10, 15a), 3.36 (s, 3H, 25), 3.21 (d, *J* = 13.0 Hz, 1H, 15b), 3.17 (s, 3H, 27), 2.89 (d, *J* = 9.7 Hz, 1H, 5), 2.57 – 2.44 (m, 2H, 2'a, 2b), 2.44 – 2.31 (m, 1H, 2'b), 2.26 (td, *J* = 6.9, 2.7 Hz, 2H, 5'), 2.23 – 2.15 (m, 1H, 2a), 1.95 (t, *J* = 2.6 Hz, 1H, 7'), 1.81 (p, *J* = 7.6 Hz, 2H, 3'), 1.68 (s, 3H, 26), 1.66 – 1.54 (m, 3H, 4', 8b), 1.54 – 1.42 (m, 1H, 6), 1.28 (d, *J* = 6.4 Hz, 3H, 23), 1.26 – 1.18 (m, 1H, 8a), 0.83 (s, 3H, 22).

4c - ^{13}C NMR (101 MHz, chloroform-*d*) δ 171.9 (1), 168.8 (1'), 156.2 (20), 152.4 (24), 142.7 (18), 140.2 (16), 140.1 (14), 132.5 (12), 128.2 (11), 124.6 (13), 122.3 (17), 119.6 (19), 113.1 (21), 88.3 (10), 84.2 (6'), 81.1 (9), 77.0 (3), 74.4 (7), 69.0 (7'), 66.5 (5), 60.4 (4), 56.9 (25), 56.7 (28), 47.3 (15), 38.6 (6), 35.9 (8), 35.7 (27), 33.7 (2'), 32.9 (2), 27.9 (4'), 23.9 (3'), 18.4 (5'), 15.9 (26), 14.6 (23), 12.2 (22).

4c - HRMS (ESI) *m/z* $[M+Na]^+$ 695.2710 (calcd for $C_{35}H_{45}ClN_2O_9Na$, 695.2711)

5c - ^{1}H NMR (400 MHz, acetone-d_6) δ 8.09 (s, 1H, NH), 7.24 (s, 1H, 21), 7.03 (s, 1H, 17), 6.67 (dd, *J* = 15.1, 11.1 Hz, 1H, 12), 6.37 (d, *J* = 11.0 Hz, 1H, 13), 5.63 (dd, *J* = 15.2, 8.2 Hz, 1H, 11), 4.72 (dd, *J* = 11.8, 2.2 Hz, 1H, 3), 4.65 – 4.49 (m, 2H, 7, 8), 4.29 (d, *J* = 8.3 Hz, 1H, 10), 4.02 (s, 3H, 28), 3.63 (d, *J* = 13.0 Hz, 1H, 15a), 3.38 (d, *J* = 12.8 Hz, 1H, 15b), 3.31 (s, 3H, 25), 3.15 (s, 3H, 27), 2.84 (d, *J* = 9.7 Hz, 1H, 5), 2.74 – 2.56 (m, 2H, 2'a, 2a), 2.39 – 2.23 (m, 4H, 2'b, 5', 7'), 2.11 – 1.99 (m, 1H, 2b), 1.81 (q, *J* = 9.5, 7.8 Hz, 5H, 3', 26), 1.75 – 1.59 (m, 2H, 4'), 1.61 – 1.52 (m, 1H, 6), 1.18 (d, *J* = 6.5 Hz, 3H, 23), 0.86 (s, 3H, 22).

5c - ^{13}C NMR (101 MHz, acetone-d_6) δ 172.7 (1'), 167.0 (1), 156.6 (20), 150.6 (24), 142.9 (18), 141.6 (16), 141.3 (14), 137.9 (9), 133.8 (12), 127.6 (11), 124.8 (13), 123.3 (17), 119.1 (19), 114.0 (21), 98.4 (8), 84.3 (6'), 80.2 (10), 79.1 (7), 78.3 (3), 70.2 (7'), 67.8 (5), 60.8 (4), 56.8 (28), 56.5 (25), 46.7 (15), 40.0 (6), 35.5 (27), 34.3 (2'), 33.0 (2), 28.4 (4'), 24.9 (3'), 18.4 (5'), 15.8 (26), 14.2 (23), 11.8 (22).

5c - HRMS (ESI) *m/z* $[M+Na]^+$ 677.2601 (calcd for $C_{35}H_{43}ClN_2O_8Na$, 677.2606)

Table S1. ^{1}H- and ^{13}C-NMR assignments for maytansinol **1b**.

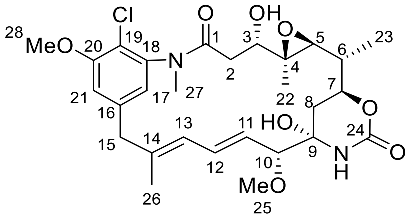

Maytansinol **1b**

Position	^{1}H-NMR[a,b] $CDCl_3$	^{1}H-NMR[a,b] acetone-d6	^{1}H-NMR[a,b] methanol-d4	^{13}C-NMR[a,c] methanol-d4
1				172.4
2	2.28 (dd, *J* = 13.3, 11.0 Hz, 1H)<br>2.12 (dd, *J* = 13.0, 1.8 Hz, 1H)	2.36 (dd, *J* = 13.0, 11.1 Hz, 1H)<br>2.13 (dt, *J* = 12.3, 1.3 Hz, 1H)	2.35 (dd, *J* = 12.9, 11.3 Hz, 1H)<br>1.95 (dd, *J* = 12.8, 2.3 Hz, 1H)	34.9[f]
3	3.54 – 3.44 (m, 3H)[d]	3.55 (dd, *J* = 5.0, 2.0 Hz, 1H)	3.41 – 3.38 (m, 4H)[e]	75.3
4				63.2
5	2.59 (d, *J* = 9.8 Hz, 1H)	2.60 (d, *J* = 9.8 Hz, 1H)	2.50 (d, *J* = 9.7 Hz, 1H)	66.9
6	1.62 – 1.48 (m, 1H)	1.56 – 1.43 (m, 1H)	1.67 – 1.52 (m, 1H)	37.6
7	4.37 (ddd, *J* = 12.3, 10.4, 1.8 Hz, 1H)	4.25 (ddd, *J* = 12.4, 10.3, 2.2 Hz, 1H)	4.30 (ddd, *J* = 12.4, 10.5, 1.9 Hz, 1H)	75.4
8	2.21 (dt, *J* = 13.9, 1.7 Hz, 1H)<br>1.28 – 1.23 (m, 1H)	1.91 (dd, *J* = 12.9, 2.0 Hz, 1H)<br>1.41 (dd, *J* = 13.9, 12.2 Hz, 1H)	2.07 (dd, *J* = 14.1, 2.0 Hz, 1H)<br>1.47 (dd, *J* = 14.1, 12.2 Hz, 1H)	34.9
9				80.8
10	3.54 – 3.44 (m, 3H) [d]	3.64 (d, *J* = 9.2 Hz, 1H)	3.65 – 3.58 (m, 1H)	88.2
11	5.53 (dd, *J* = 15.2, 9.2 Hz, 1H)	5.52 (dd, *J* = 15.3, 9.2 Hz, 1H)	5.58 – 5.46 (m, 1H)	126.9
12	6.44 (dd, *J* = 15.3, 10.9 Hz, 1H)	6.70 (dd, *J* = 15.3, 11.0 Hz, 1H)	6.66 – 6.53 (m, 1H)	133.5
13	6.15 (d, *J* = 10.9 Hz, 1H)	6.20 (d, *J* = 11.0 Hz, 1H)	6.18 (d, *J* = 10.9 Hz, 1H)	125.0
14				138.6
15	3.54 – 3.44 (m, 3H)[d]<br>3.12 (d, *J* = 12.7 Hz, 1H)	3.51 (d, *J* = 12.6 Hz, 1H)<br>3.28 (d, *J* = 12.6 Hz, 1H)	3.50 (d, *J* = 12.6 Hz, 1H)<br>3.24 (d, *J* = 12.6 Hz, 1H)	46.2
16				140.8
17	6.81 (d, *J* = 1.9 Hz, 1H)	7.15 (d, *J* = 1.9 Hz, 1H)	7.12 (d, *J* = 1.8 Hz, 1H)	123.5
18				141.8
19				117.9
20				155.6
21	7.04 (d, *J* = 1.8 Hz, 1H)	7.17 (d, *J* = 1.9 Hz, 1H)	7.08 (d, *J* = 1.9 Hz, 1H)	113.1
22	0.84 (s, 3H)	0.90 (s, 3H)	0.85 (s, 3H)	10.0
23	1.31 (d, *J* = 6.4 Hz, 3H)	1.21 (d, *J* = 6.4 Hz, 3H)	1.26 (s, 2H)	13.3
24				153.9
25	3.36 (s, 3H)	3.34 (s, 3H)	3.38 (m, 4H)[e]	55.4
26	1.69 (d, *J* = 1.3 Hz, 3H)	1.75 (s, 3H)	1.73 (d, *J* = 1.3 Hz, 3H)	14.4
27	3.21 (s, 3H)	3.14 (s, 3H)	3.19 (s, 3H)	34.9[f]
28	3.99 (s, 3H)	4.00 (s, 3H)	3.98 (s, 3H)	55.7
3-OH	-	4.50 (dd, *J* = 5.0, 1.1 Hz, 1H)	-	
9-OH	-	4.80 (d, *J* = 1.8 Hz, 1H)	-	
NH	6.35 (d, *J* = 1.4 Hz, 1H)	6.42 (s, 1H)	-	

[a] Chemical shifts (in ppm) were determined with reference to TMS; [b] Spectra determined at 400 MHz; [c] Spectra determined at 101 MHz; [d-f] Chemical shifts bearing the same symbol overlap.

Table S2. ^1H- and ^{13}C-NMR assignments for **3** in deuterated chloroform.

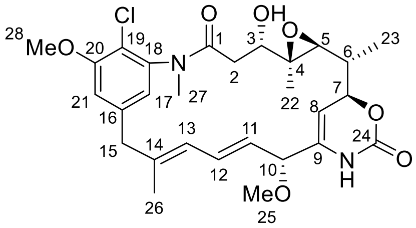

3

Position	^1H-NMR[a,b] $CDCl_3$	^{13}C-NMR[a,c]
1		171.6
2	2.32 (dd, *J* = 13.6, 10.8 Hz, 1H)<br>2.14 (dd, *J* = 13.7, 2.3 Hz, 1H)	35.7
3	3.59 (dd, *J* = 10.9, 2.4 Hz, 1H)	75.9
4		62.4
5	2.55 (d, *J* = 9.7 Hz, 1H)	66.2
6	1.66 (td, *J* = 10.1, 6.4 Hz, 1H)	39.0
7	4.78 (dd, *J* = 10.5, 2.3 Hz, 1H)	80.1
8	5.02 – 4.96 (m, 1H)	99.1
9		136.1
10	4.20 – 4.12 (m, 1H)	80.0
11	5.54 (dd, *J* = 15.2, 8.5 Hz, 1H)	126.9
12	6.46 (dd, *J* = 15.2, 10.8 Hz, 1H)	133.5
13	6.16 (d, *J* = 11.1 Hz, 1H)	124.7
14		140.3
15	3.49 (d, *J* = 12.8 Hz, 1H)<br>3.15 (d, *J* = 12.9 Hz, 1H)	46.8
16		140.4
17	6.97 (d, *J* = 1.9 Hz, 1H)	123.3
18		143.0
19		119.0
20		156.1
21	6.83 (d, *J* = 1.9 Hz, 1H)	112.5
22	0.84 (s, 3H)	11.2
23	1.27 (d, *J* = 2.3 Hz, 3H)	14.6
24		151.5
25	3.35 (s, 3H)	56.6
26	1.75 (s, 3H)	16.4
27	3.23 (s, 3H)	36.0
28	4.00 (s, 3H)	56.7
3-OH	-	
9-OH	-	
NH	7.08 (s, 1H)	

[a] Chemical shifts (in ppm) were determined with reference to TMS; [b] Spectra determined at 400 MHz; [c] Spectra determined at 101 MHz; [d-f] Chemical shifts bearing the same symbol overlap.

Table S3. **^{1}H**- and ^{13}C-NMR assignments for **4a** and **5a** in acetone-d6.

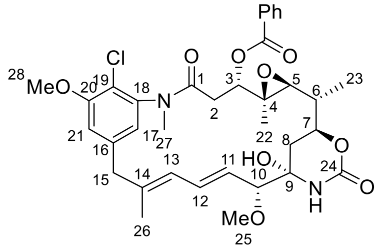

4a

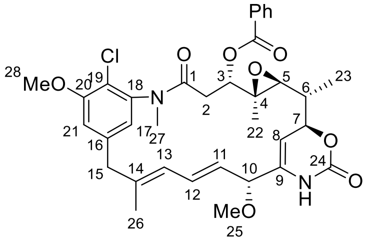

5a

Position	^{1}H-NMR[a,b] (4a)	^{13}C-NMR[a,c] (4a)	^{1}H-NMR[a,b] (5a)	^{13}C-NMR[a,c] (5a)
1		168.0		168.0
2	2.88 – 2.79 (m, 1H)<br>2.26 (dd, *J* = 14.4, 3.1 Hz, 1H)	32.7	2.91 (dd, *J* = 14.6, 12.0 Hz, 1H)<br>2.22 (dd, *J* = 14.6, 3.0 Hz, 1H)	32.2
3	5.04 – 4.85 (m, 2H)[d]	77.5	5.02 (dd, *J* = 11.9, 3.0 Hz, 1H)	77.9
4		60.5		60.2
5	3.05 (d, *J* = 9.5 Hz, 1H)	66.4	3.03 (d, *J* = 9.6 Hz, 1H)	67.0
6	1.54 – 1.39 (m, 2H, 6)[e]	38.7	1.65 (ddd, *J* = 10.6, 9.7, 6.5 Hz, 1H)	39.5
7	4.31 – 4.14 (m, 1H)	73.8	4.56 (dd, *J* = 10.8, 3.4 Hz, 1H)	78.2
8	1.59 (dt, *J* = 13.6, 1.9 Hz, 1H)<br>1.54 – 1.39 (m, 2H, 6)[e]	36.2	4.43 (dt, *J* = 3.2, 1.5 Hz, 1H)	98.0
9		80.5		137.0
10	3.50 (d, *J* = 9.1 Hz, 1H)	88.9	4.15 (d, *J* = 8.5 Hz, 1H)	79.5
11	5.04 – 4.85 (m, 2H)[d]	128.6	4.88 (dd, *J* = 15.3, 8.4 Hz, 1H)	126.7
12	6.61 (dd, *J* = 15.5, 11.1 Hz, 1H)	132.4	6.54 (dd, *J* = 15.3, 10.9 Hz, 1H)	133.0
13	6.04 (d, *J* = 11.0 Hz, 1H)	124.7	6.13 (d, *J* = 11.0 Hz, 1H)	124.1
14		139.6		140.7
15	3.59 (d, *J* = 12.6 Hz, 1H)<br>3.36 (d, *J* = 12.7 Hz, 1H)	46.3	3.66 (d, *J* = 12.7 Hz, 1H)<br>3.41 (d, *J* = 12.6 Hz, 1H)	46.2
16		141.3		141.2
17	7.19 (d, *J* = 1.8 Hz, 1H, 17)	122.1	7.17 (d, *J* = 1.8 Hz, 1H)	122.2
18		142.4		142.3
19		118.7		118.7
20		156.2		156.2
21	7.27 (d, *J* = 1.9 Hz, 1H)	113.5	7.28 (d, *J* = 1.8 Hz, 1H)	113.4
22	0.97 (s, 3H)	12.1	0.90 (s, 3H)	11.7
23	1.24 (d, *J* = 6.4 Hz, 3H)	14.0	1.19 (d, *J* = 6.5 Hz, 3H)	13.5
24		151.1		149.9
25	3.24 (s, 3H)	55.8	3.15 (s, 3H)	55.4
26	1.77 (s, 3H)	14.9	1.79 (s, 3H)	15.1
27	3.13 (s, 3H)	34.6	3.14 (s, 3H)	34.7
28	4.04 (s, 3H)	56.2	4.04 (s, 3H)	56.2
9-OH	4.47 (s, 1H)			
NH	6.33 (s, 1H)		8.03 (s, 1H)	

[a] Chemical shifts (in ppm) were determined with reference to TMS; [b] Spectra determined at 400 MHz; [c] Spectra determined at 101 MHz; [d-e] Chemical shifts bearing the same symbol overlap.

Table S4. ^{1}H- and ^{13}C-NMR assignments for **6a** in deuterated chloroform and **7a** in acetone-d6.

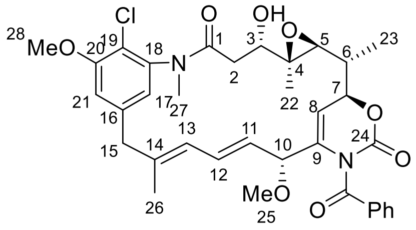

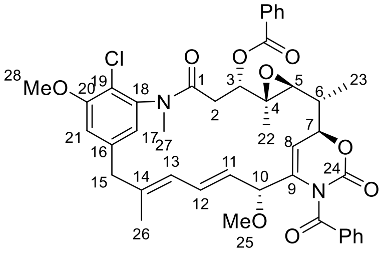

Position	**6a**		**7a**	
	^{1}H-NMR[a,b]	^{13}C-NMR[a,c]	^{1}H-NMR[a,b]	^{13}C-NMR[a,c]
1		172.2		168.0
2	2.37 (dd, *J* = 13.7, 10.9 Hz, 1H)<br>2.12 (d, *J* = 13.6 Hz, 1H)	36.4	2.99 – 2.87 (m, 1H)<br>2.27 (d, *J* = 14.2 Hz, 1H)	32.3
3	3.61 (d, *J* = 9.7 Hz, 1H)	76.5	5.04 (d, *J* = 11.6 Hz, 1H)	78.2
4		63.6		60.2
5	2.59 (d, *J* = 9.6 Hz, 1H)	67.1	3.12 (d, *J* = 9.2 Hz, 1H)	66.9
6	1.68 (q, *J* = 4.9, 4.5 Hz, 1H)	40.6	1.75 (dq, *J* = 8.3, 5.0, 4.5 Hz, 1H)	40.4
7	4.86 (d, *J* = 10.8 Hz, 1H)	80.4	4.92 – 4.76 (m, 3H)[d]	78.4
8	5.43 – 5.33 (m, 1H)	103.1	4.92 – 4.76 (m, 3H)[d]	102.1
9		137.6		137.3
10	4.42 (d, *J* = 8.4 Hz, 1H)	78.5	4.35 (d, *J* = 8.2 Hz, 1H)	77.6
11	5.54 (dd, *J* = 15.2, 8.4 Hz, 1H)	127.1	4.92 – 4.76 (m, 3H)[d]	125.8
12	6.48 (dd, *J* = 15.2, 10.8 Hz, 1H)	134.4	6.64 (dd, *J* = 15.3, 11.1 Hz, 1H)	134.5
13	6.15 (d, *J* = 10.8 Hz, 1H)	125.4	6.12 (d, *J* = 11.0 Hz, 1H)	123.8
14		140.7		141.6
15	3.56 – 3.42 (m, 1H)<br>3.12 (d, *J* = 13.0 Hz, 1H)	47.3	3.64 (d, *J* = 12.5 Hz, 1H)<br>3.41 (d, *J* = 12.6 Hz, 1H)	46.2
16		141.0		141.1
17	7.01 (d, *J* = 1.8 Hz, 1H)	123.8	7.19 (s, 1H)	122.2
18		143.0		142.3
19		119.4		118.7
20		156.5		156.3
21	6.82 (d, *J* = 1.8 Hz, 1H)	112.9	7.30 (s, 1H)	113.4
22	0.85 (s, 3H)	11.6	0.97 (s, 3H)	11.6
23	1.26 (m, 3H)	14.9	1.24 (d, *J* = 6.4 Hz, 3H)	13.5
24		149.7		148.3
25	2.82 (s, 3H)	56.2	2.68 (s, 3H)	54.6
26	1.76 (s, 3H)	16.9	1.83 (s, 3H)	15.2
27	3.23 (s, 3H)	36.6	3.17 (s, 3H)	34.7
28	3.99 (s, 3H)	57.2	4.05 (s, 3H)	56.2
3-OH	-			

[a] Chemical shifts (in ppm) were determined with reference to TMS; [b] Spectra determined at 400 MHz; [c] Spectra determined at 101 MHz; [d] Chemical shifts bearing the same symbol overlap.

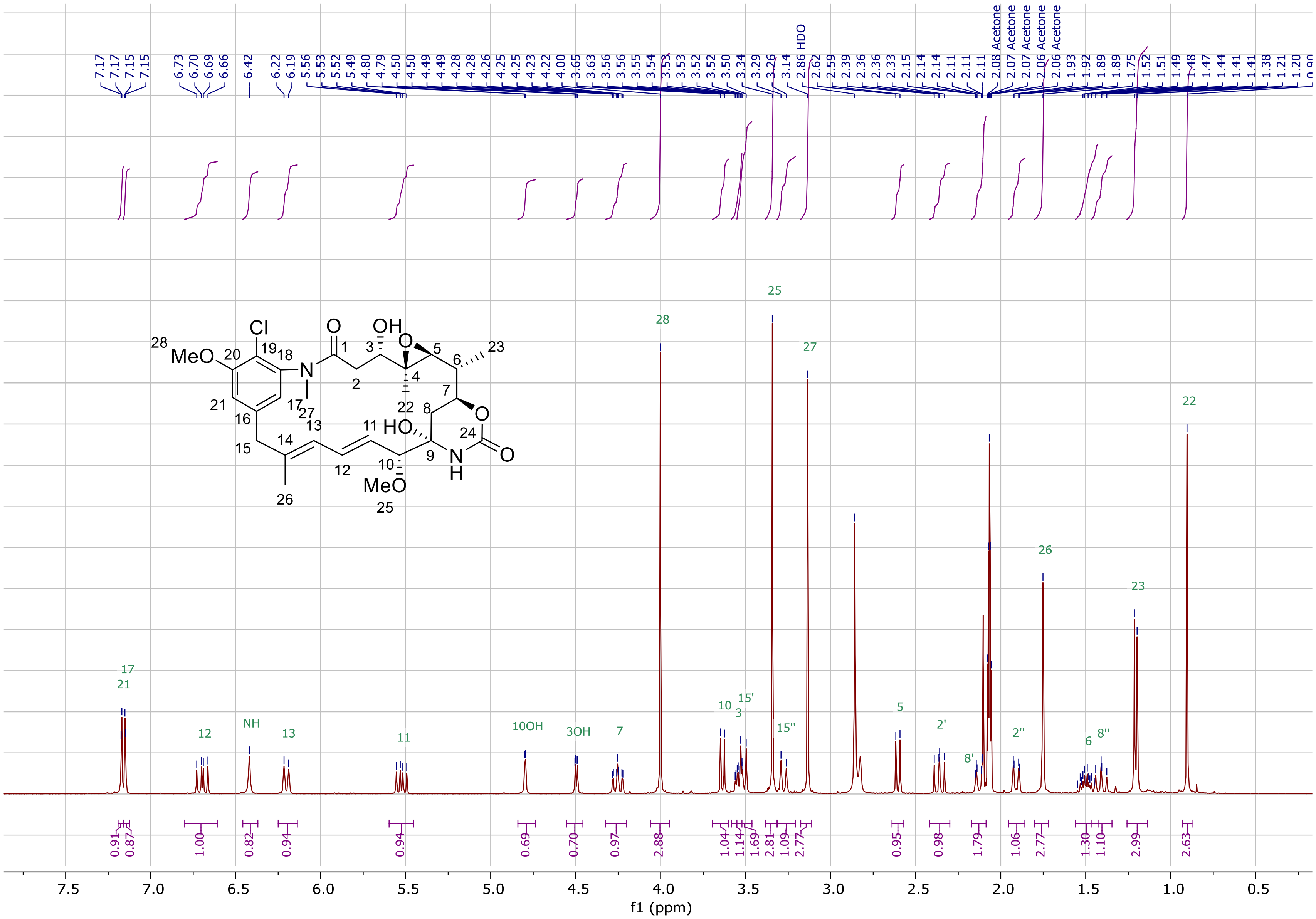

Figure S1. ^{1}H NMR spectrum (400 MHz, acetone-d6) of maytansinol **1b**

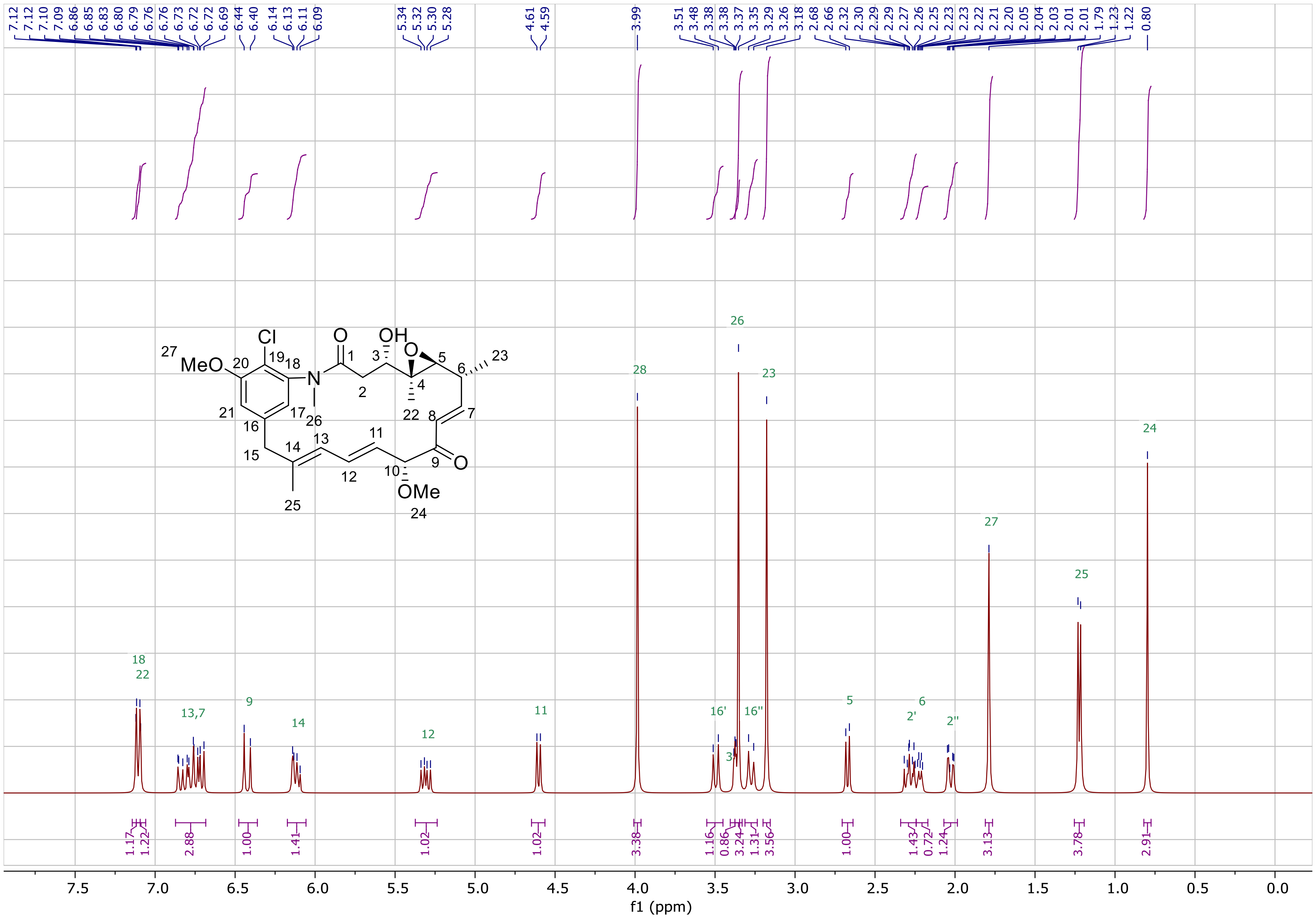

Figure S2. ^{1}H NMR spectrum (400 MHz, methanol-d4) of **2**

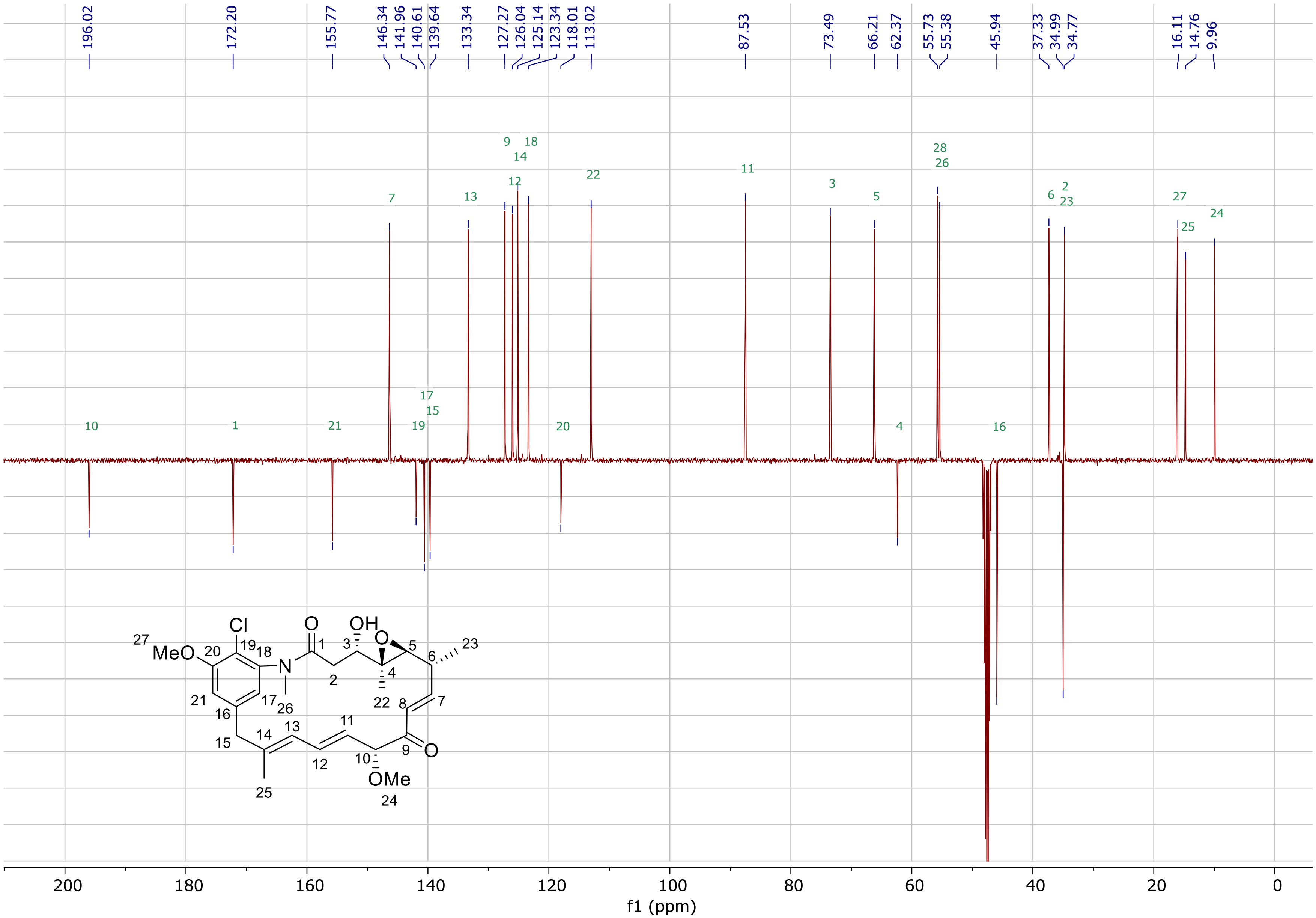

Figure S3. ^{13}C NMR APT spectrum (101 MHz, methanol-d4) of **2**

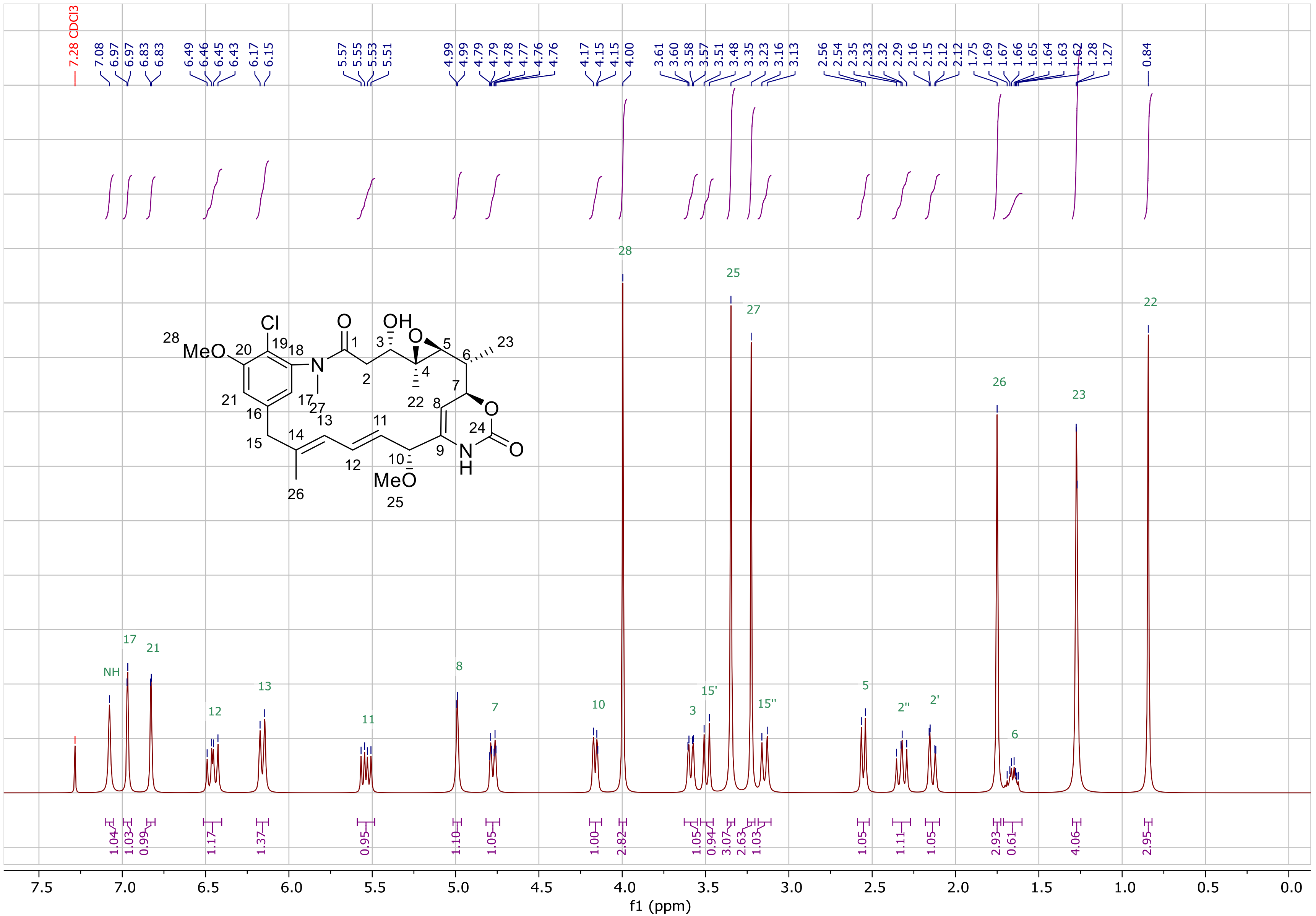

Figure S4. ^{1}H NMR spectrum (400 MHz, $CDCl_3$) of **3**

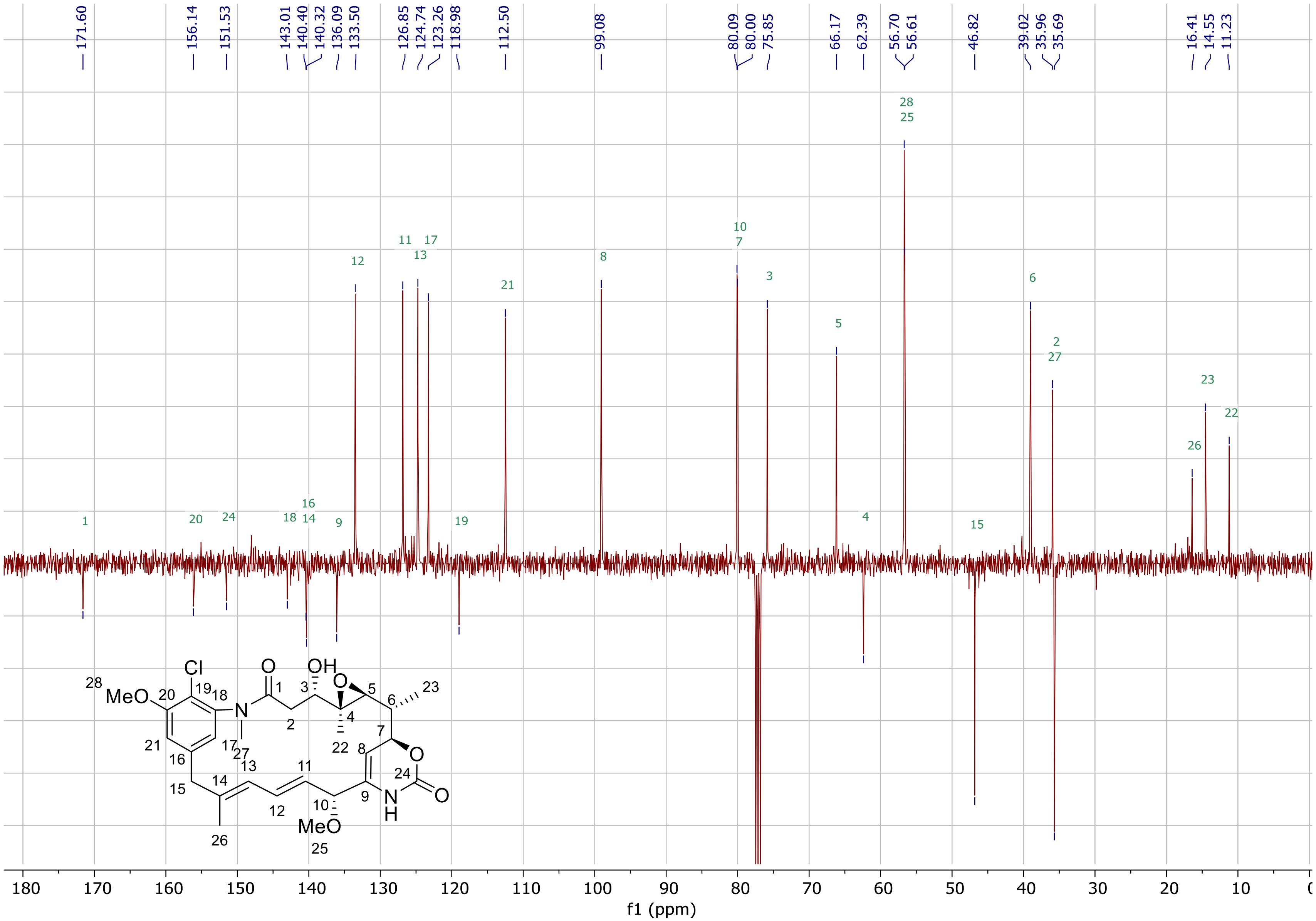

Figure S5. ^{13}C NMR APT spectrum (101 MHz, CDCl$_3$) of **3**

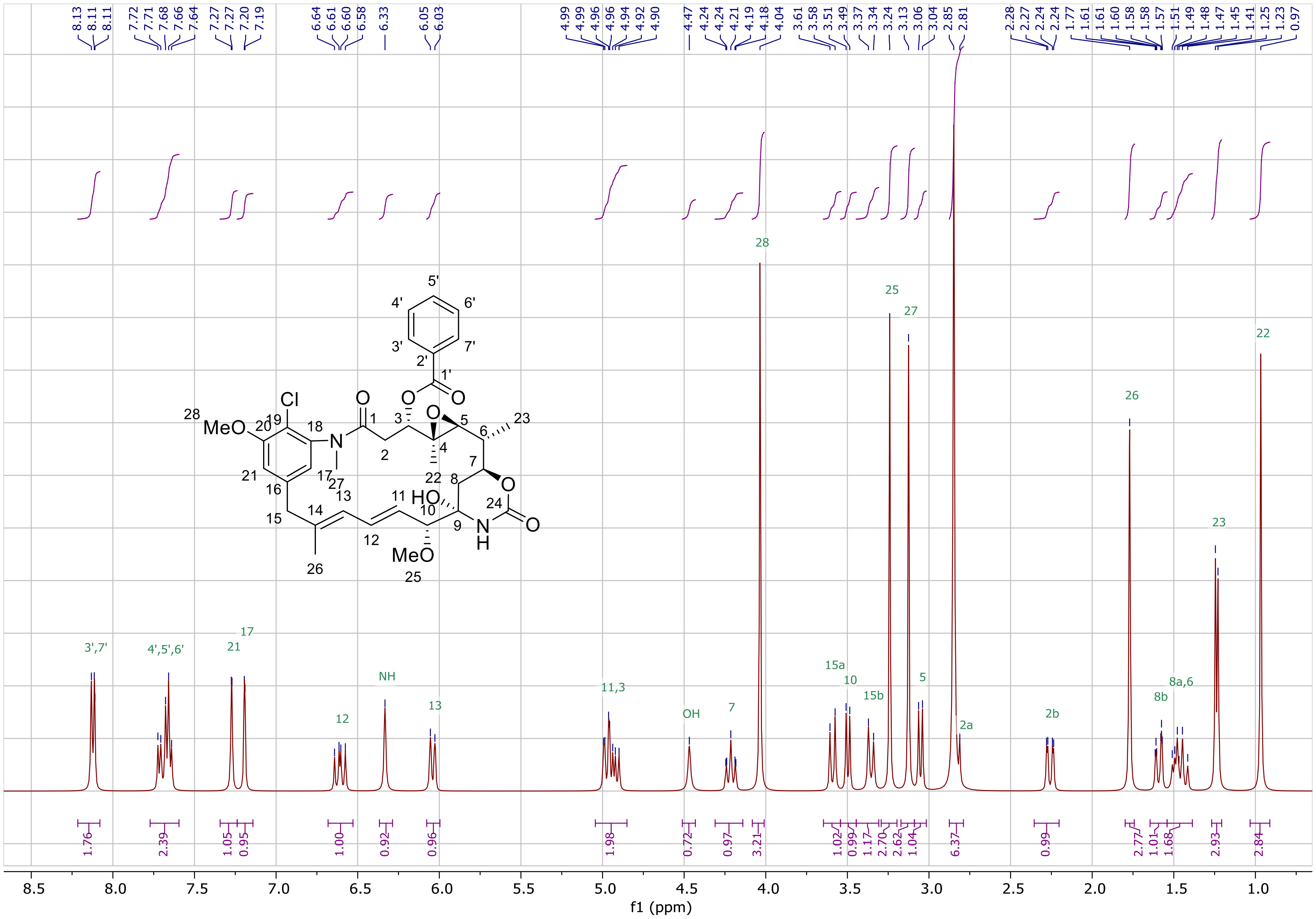

Figure S6. ^{1}H NMR spectrum (400 MHz, acetone-d6) of **4a**

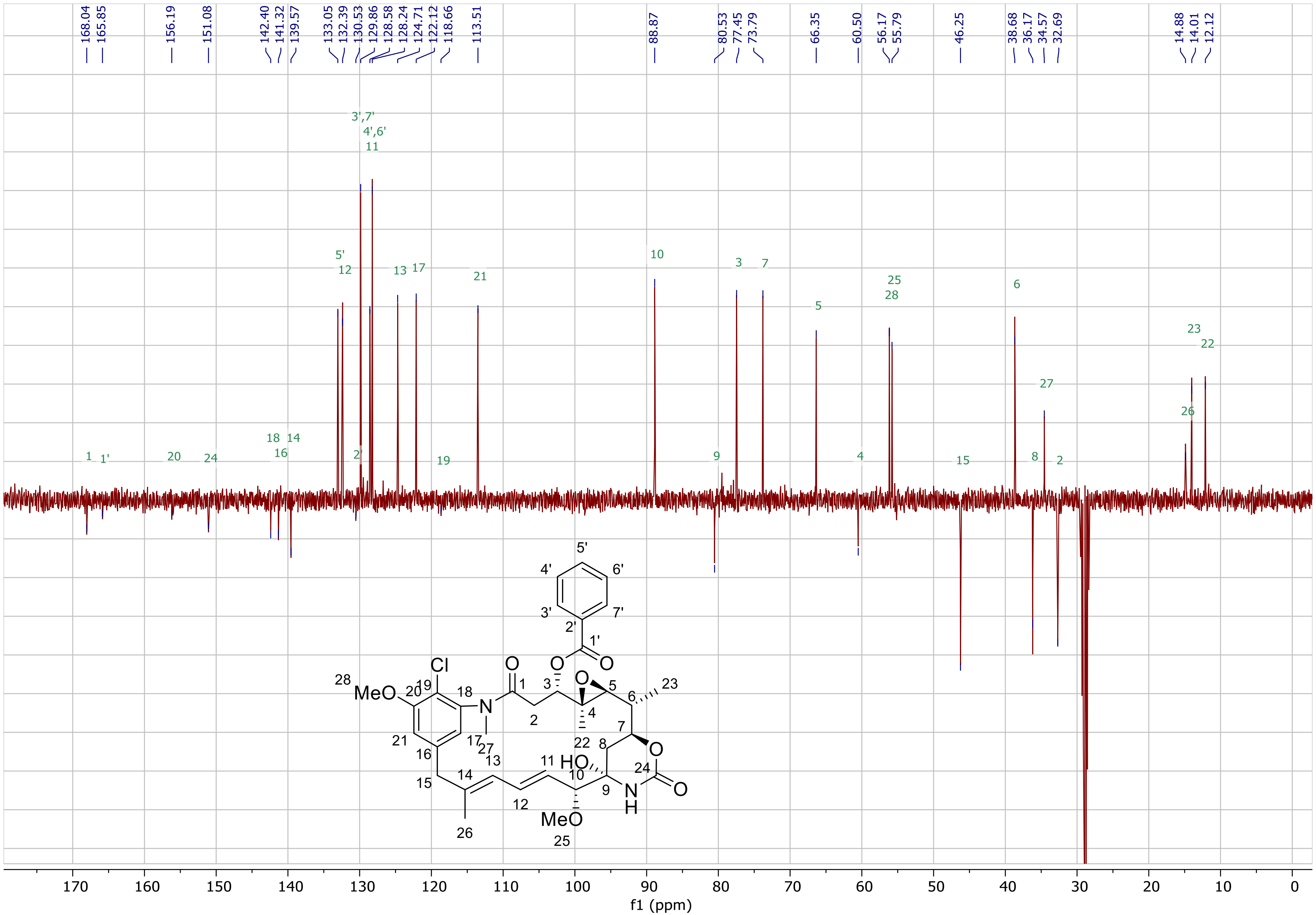

Figure S7. ^{13}C NMR APT spectrum (101 MHz, acetone-d6) of **4a**

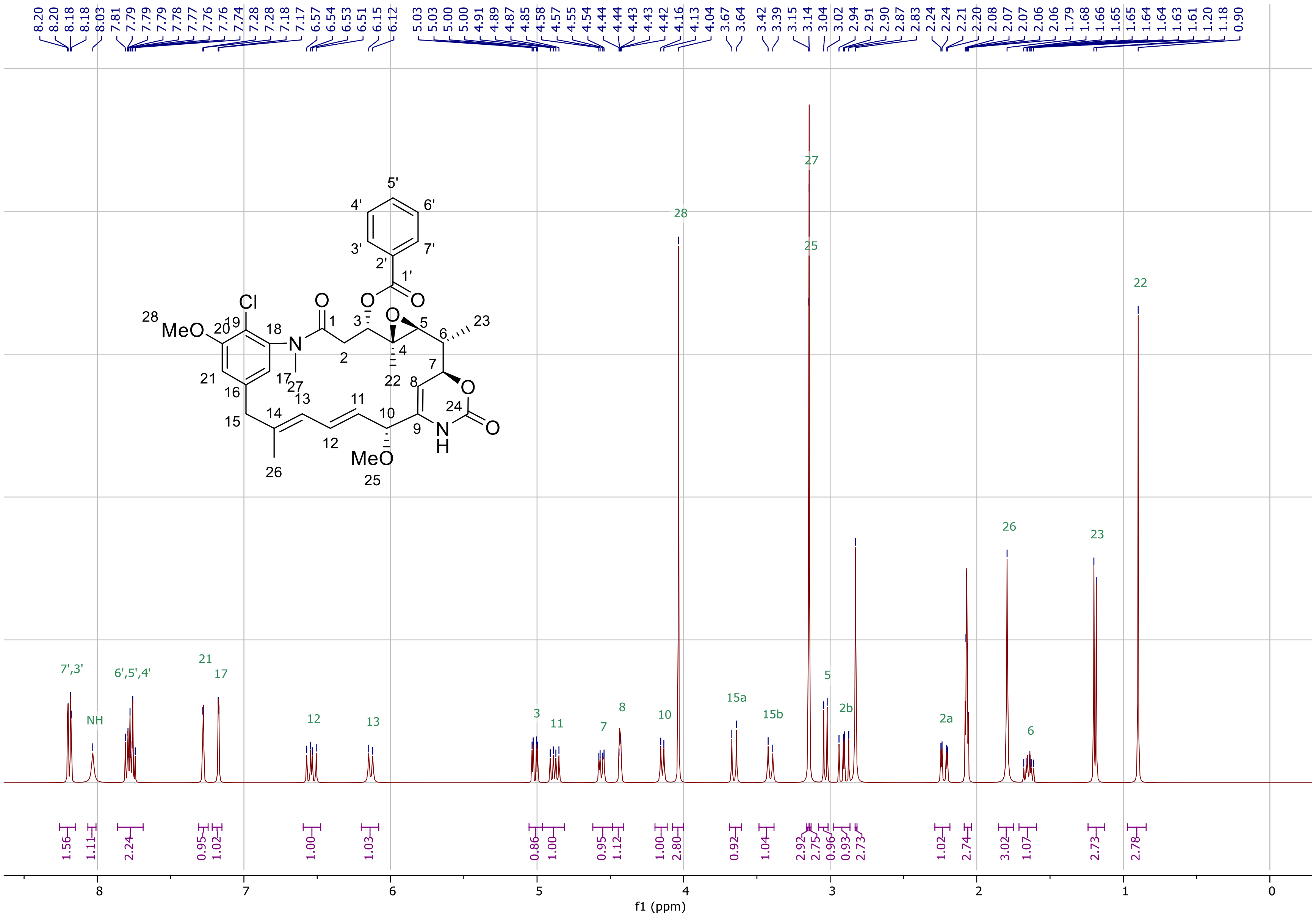

Figure S8. ^{1}H NMR spectrum (400 MHz, acetone-d6) of **5a**

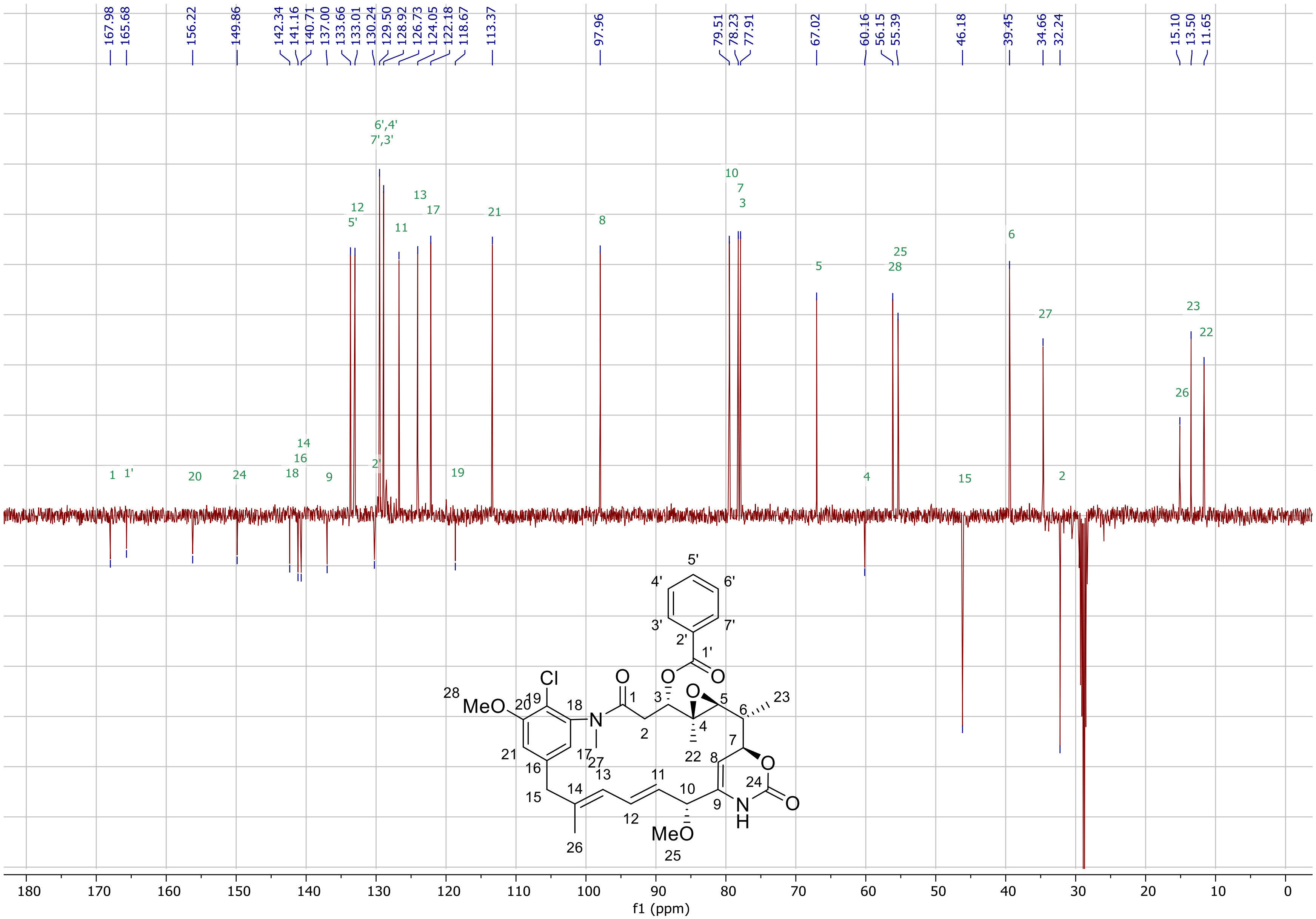

Figure S9. ^{13}C NMR APT spectrum (101 MHz, acetone-d6) of **5a**

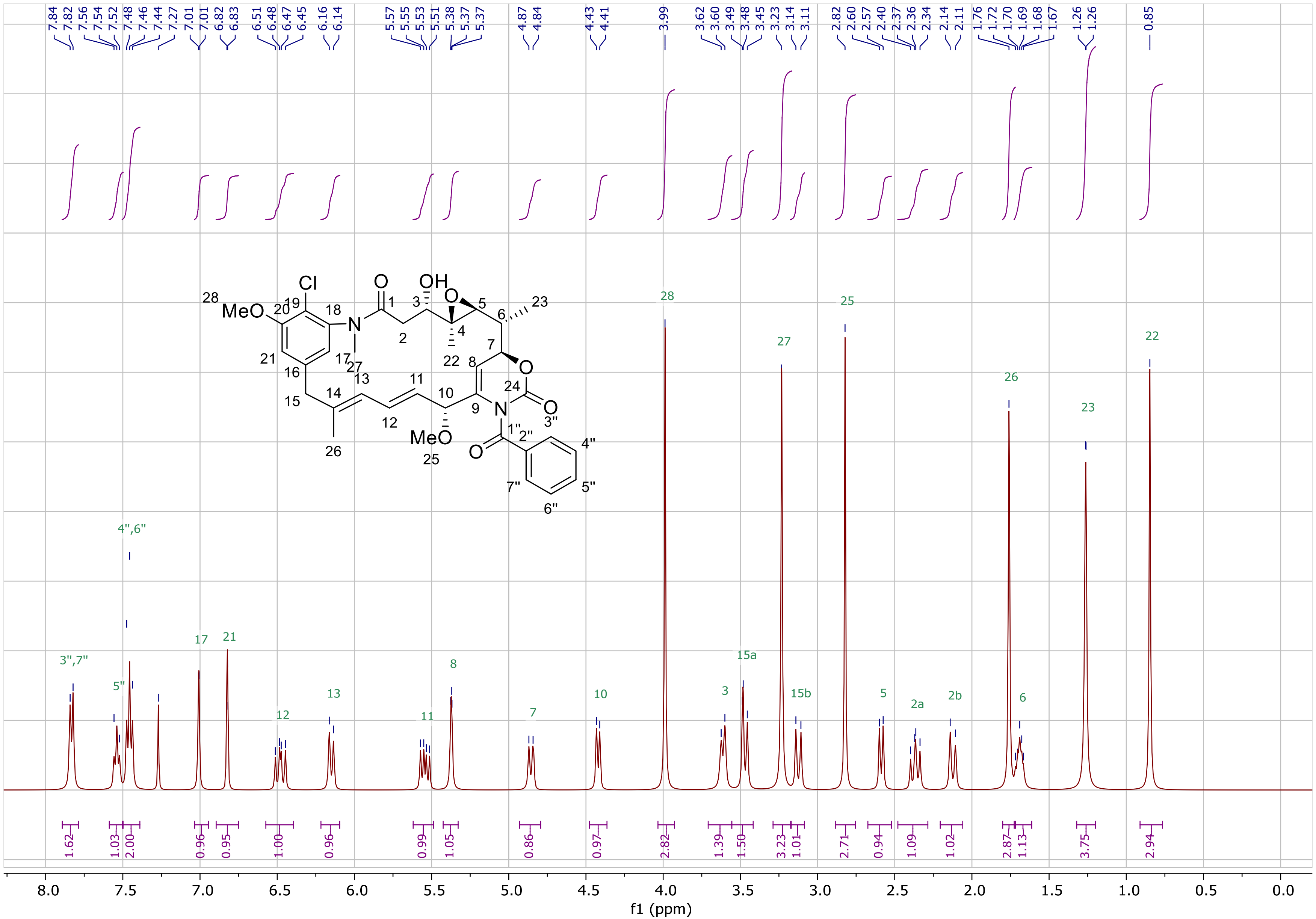

Figure S10. ^{1}H NMR spectrum (400 MHz, CDCl3) of **6a**

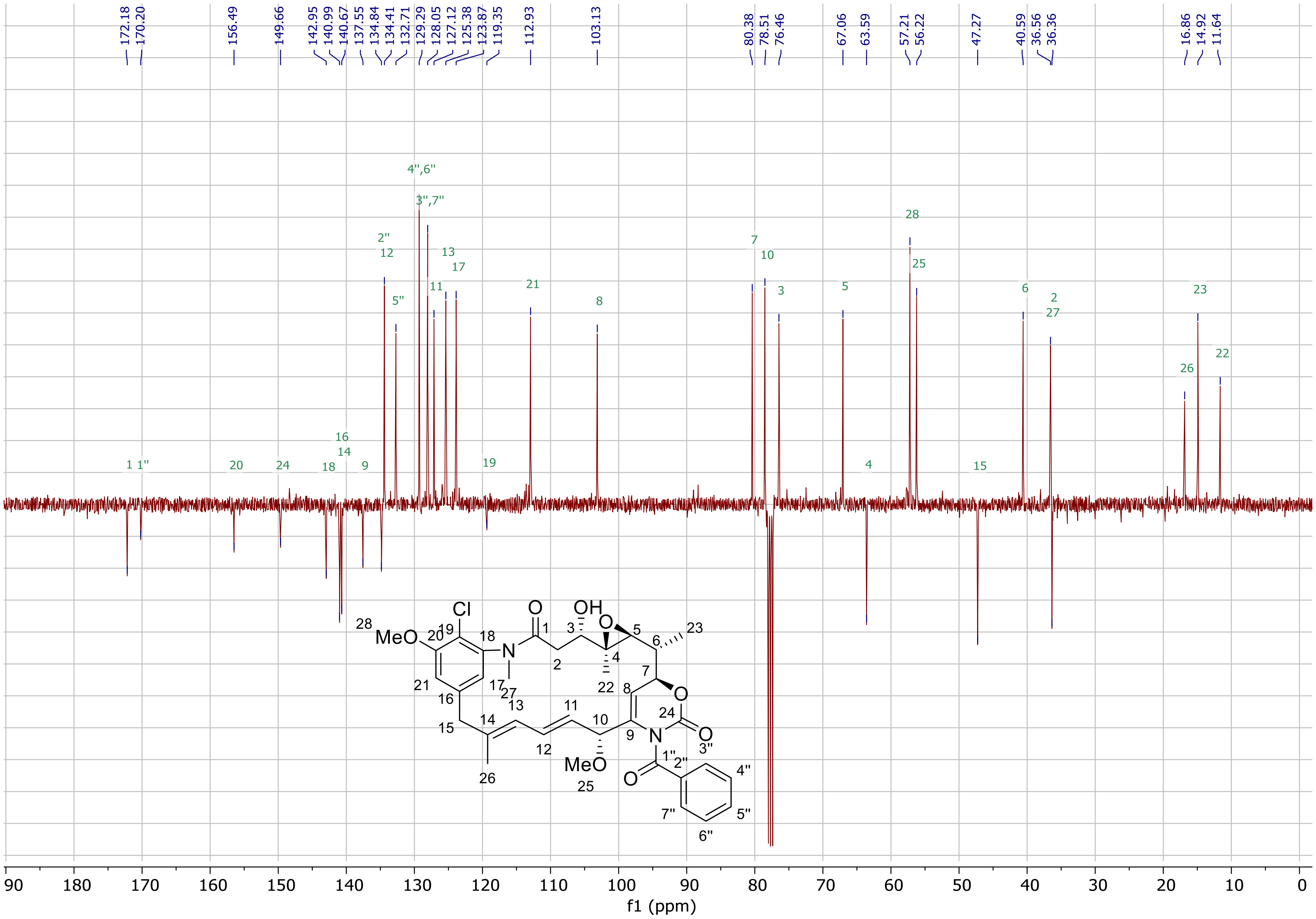

Figure S11. ^{13}C NMR APT spectrum (101 MHz, $CDCl_3$) of **6a**

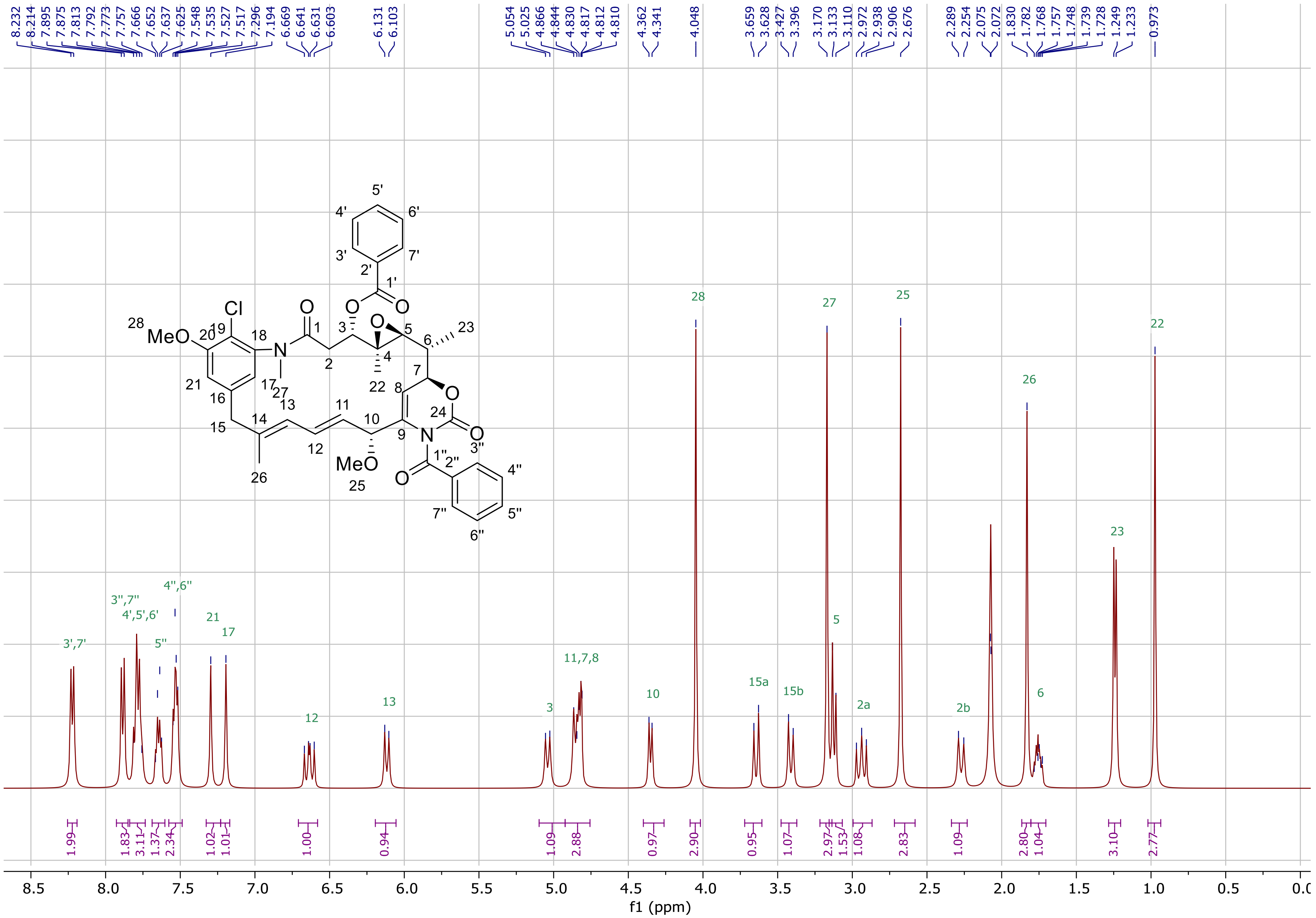

Figure S12. ^{1}H NMR spectrum (400 MHz, acetone-d6) of **7a**

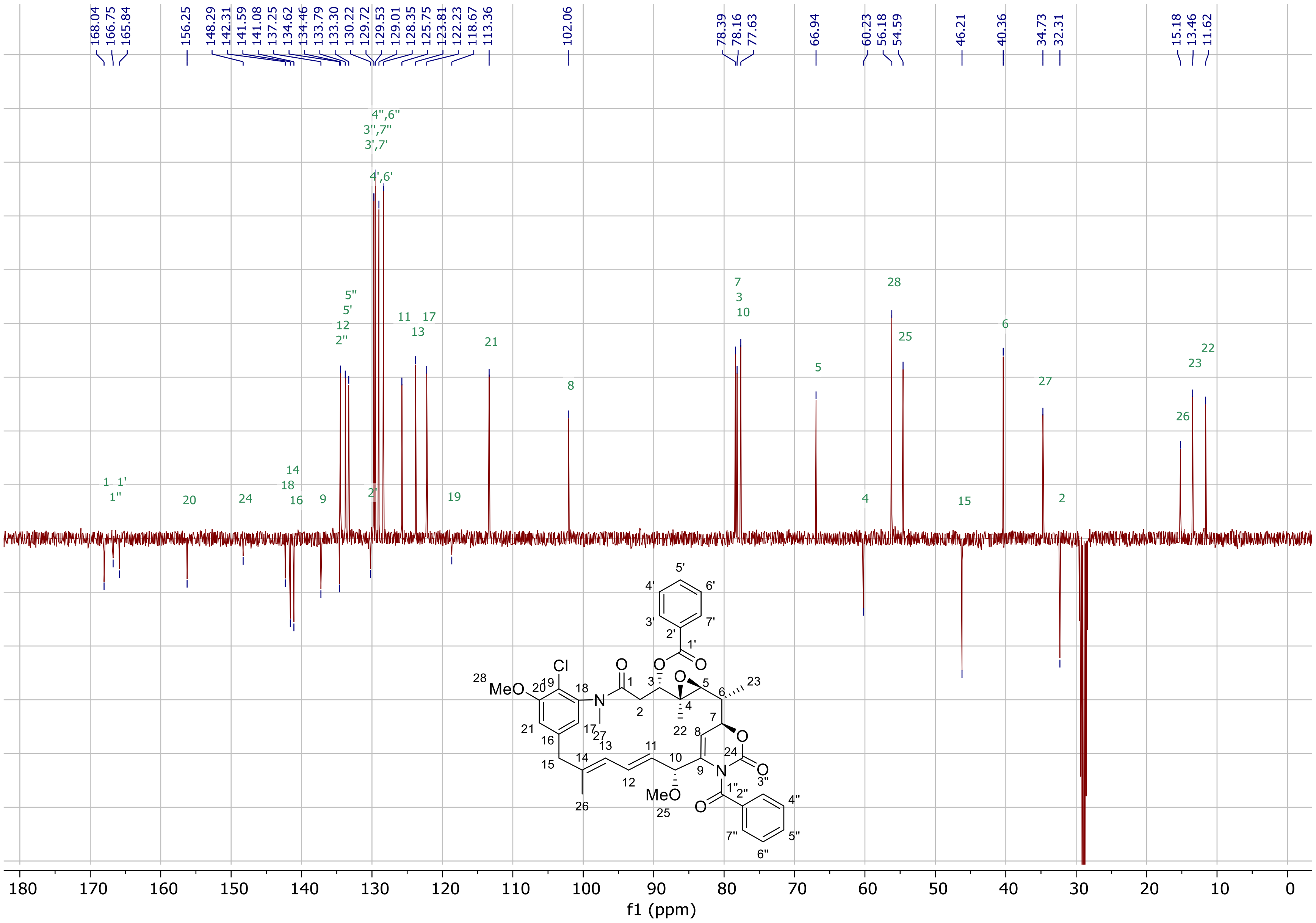

Figure S13. ^{13}C NMR APT spectrum (101 MHz, acetone-d6) of **7a**

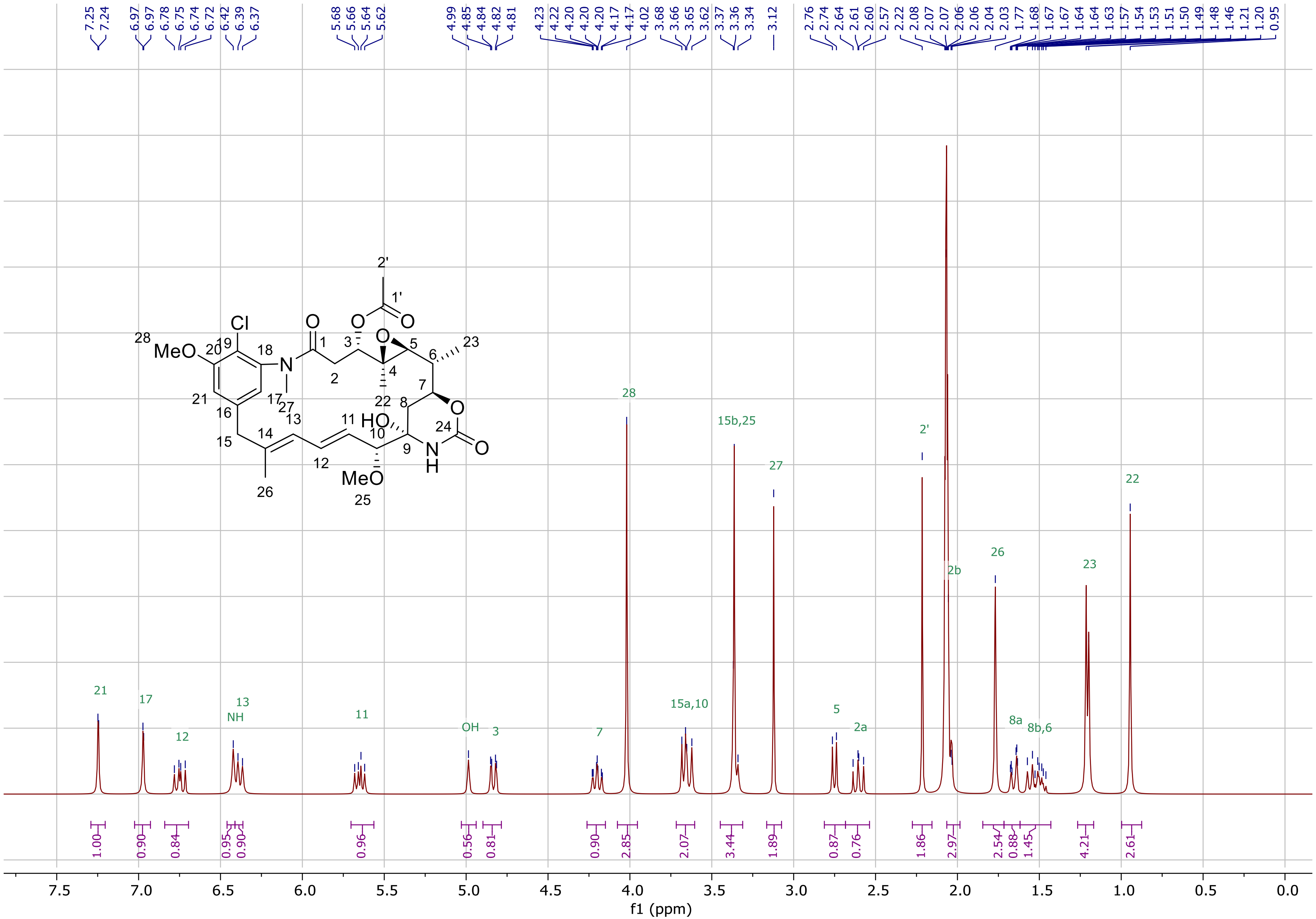

Figure S14. ^{1}H NMR spectrum (400 MHz, acetone-d6) of **4b**

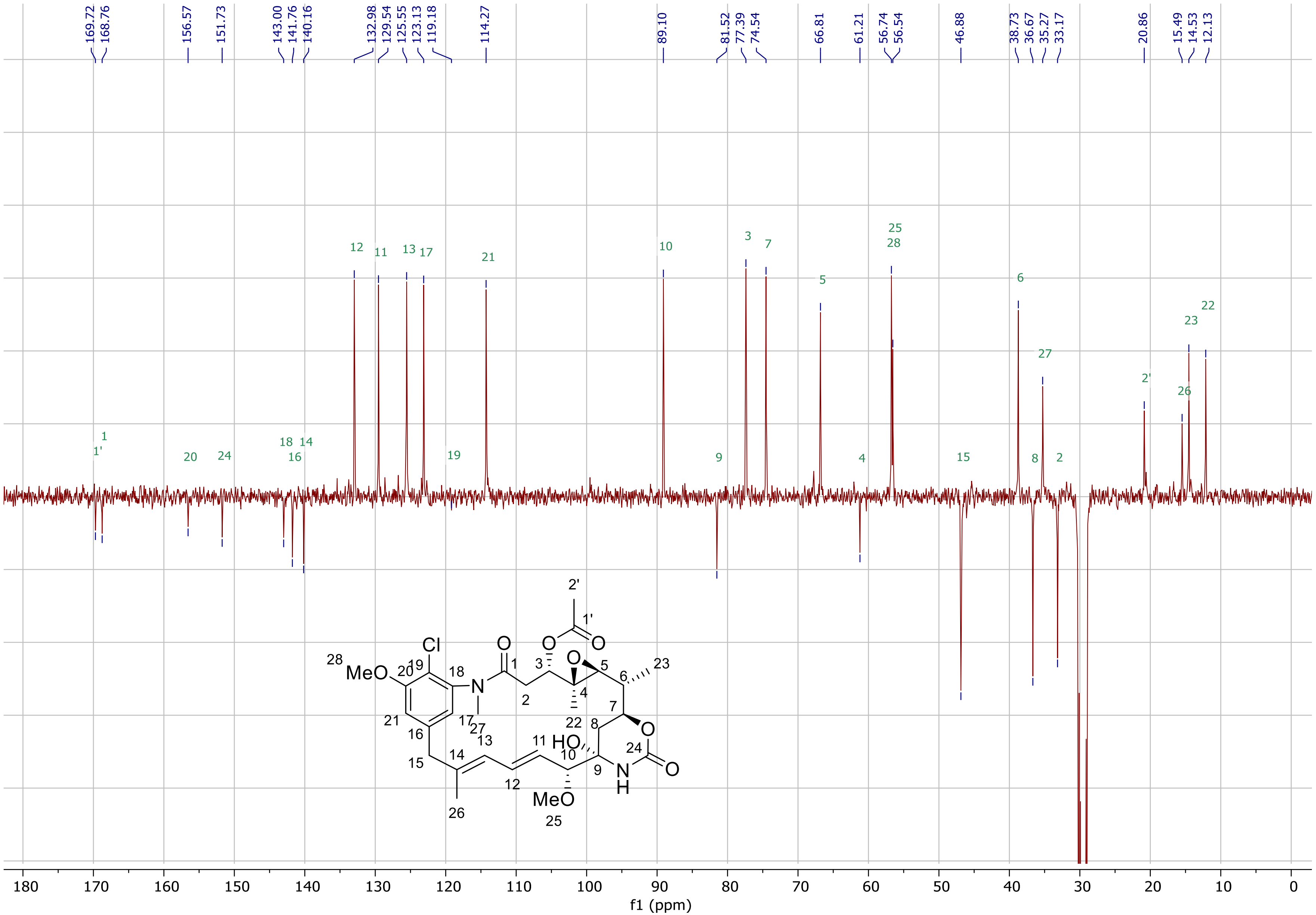

Figure S15. ^{13}C NMR APT spectrum (101 MHz, acetone-d6) of **4b**

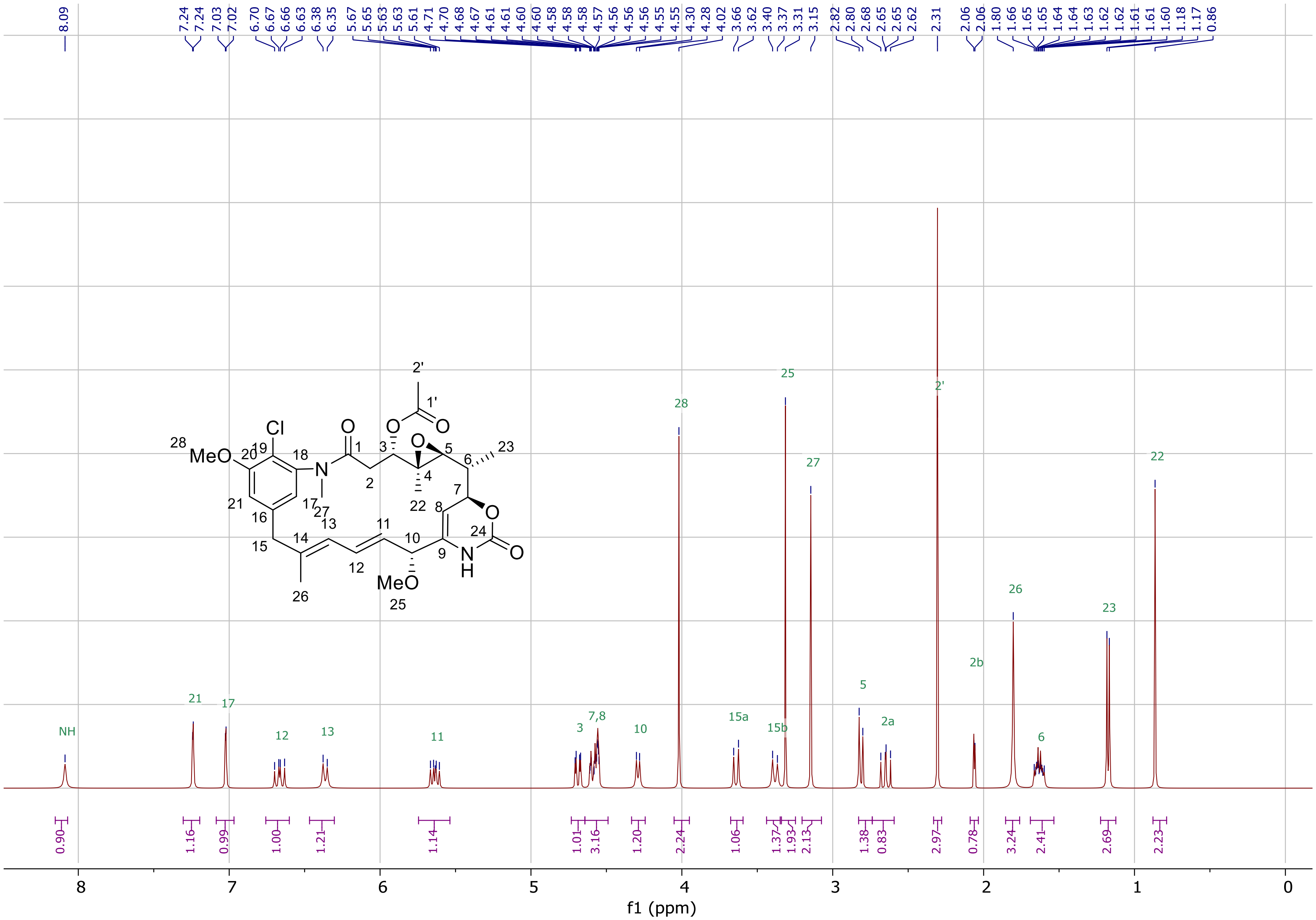

Figure S16. ^{1}H NMR spectrum (400 MHz, acetone-d6) of **5b**

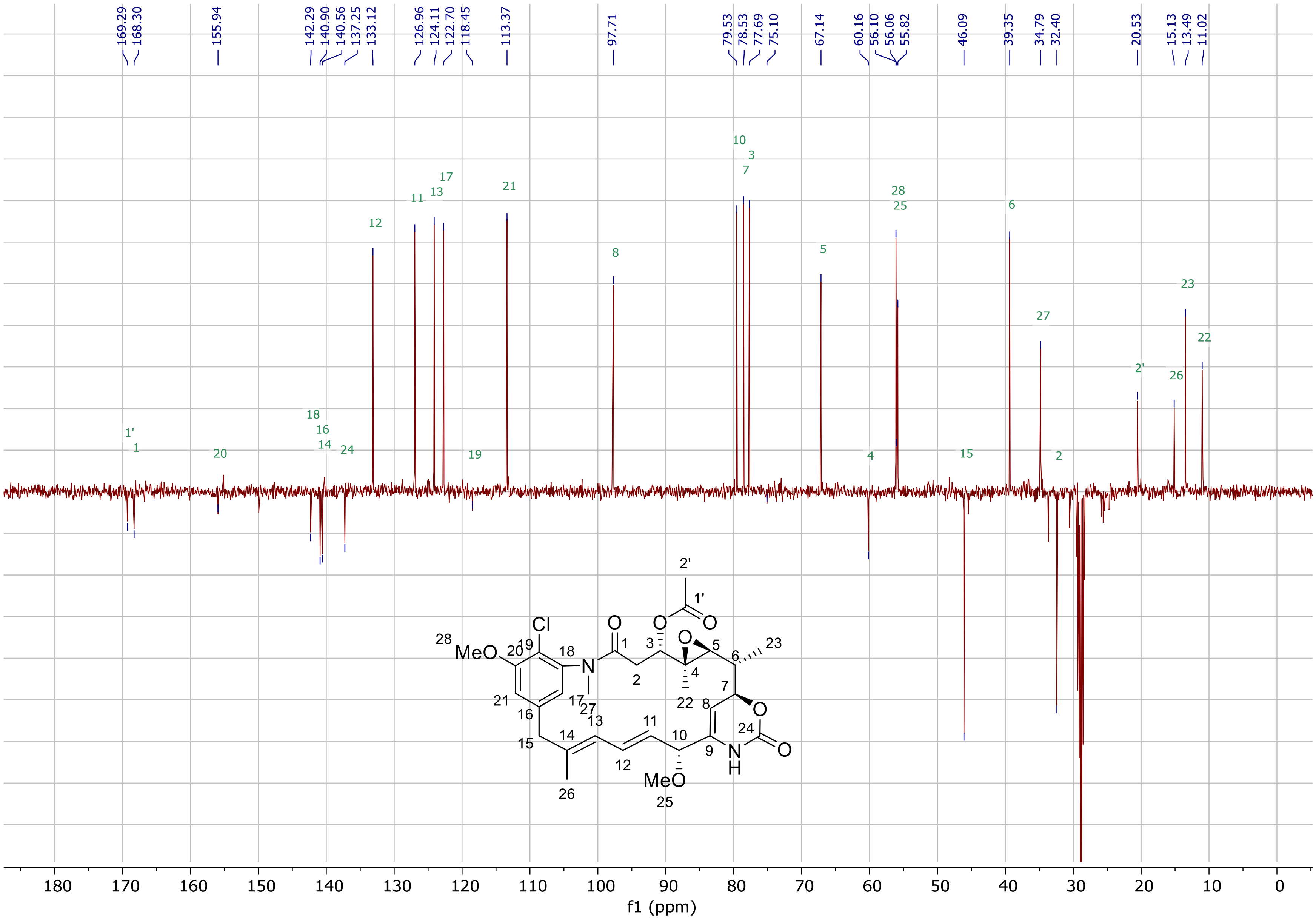

Figure S17. ^{13}C NMR APT spectrum (101 MHz, acetone-d6) of **5b**

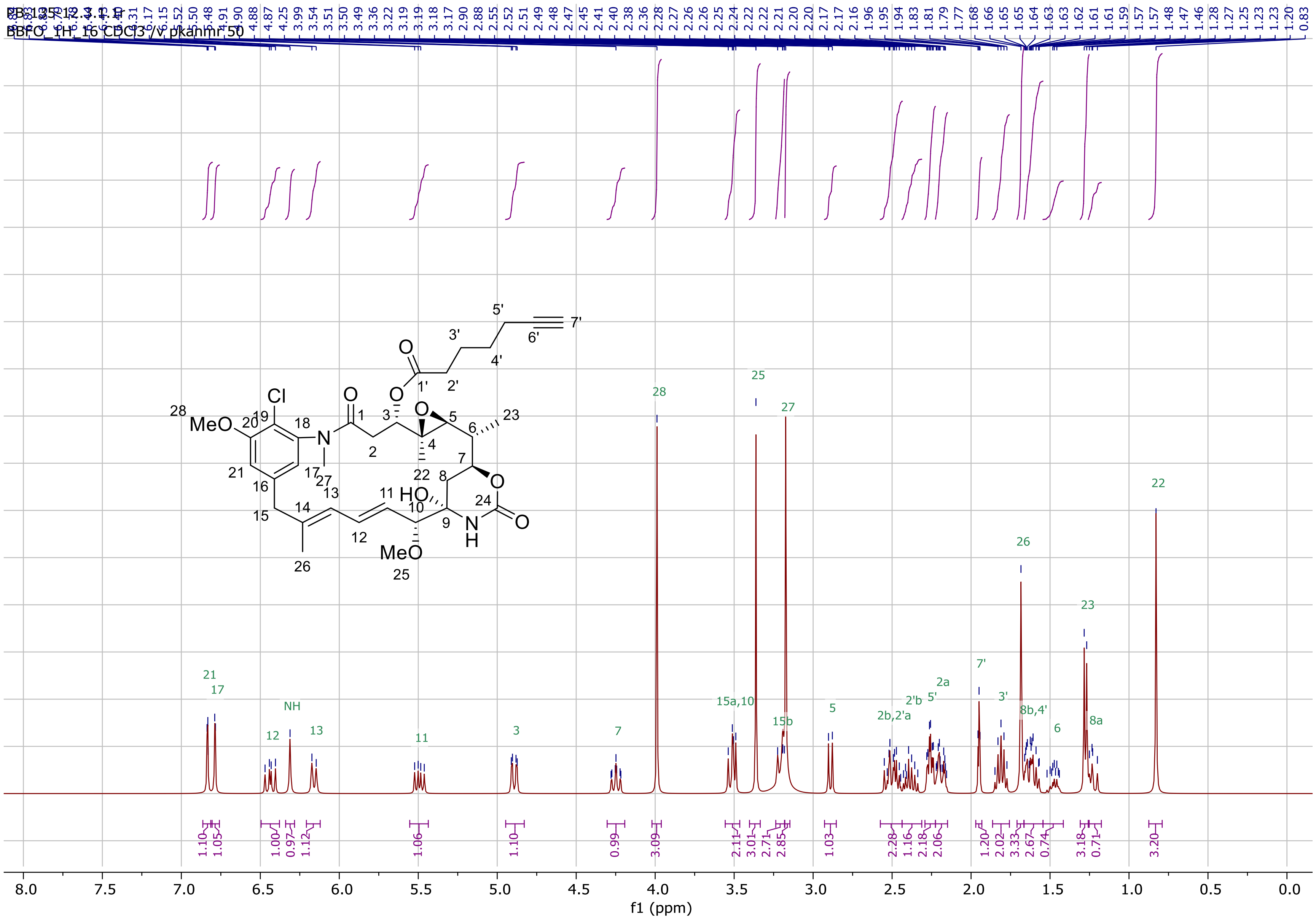

Figure S18. ^{1}H NMR spectrum (400 MHz, CDCl3) of **4c**

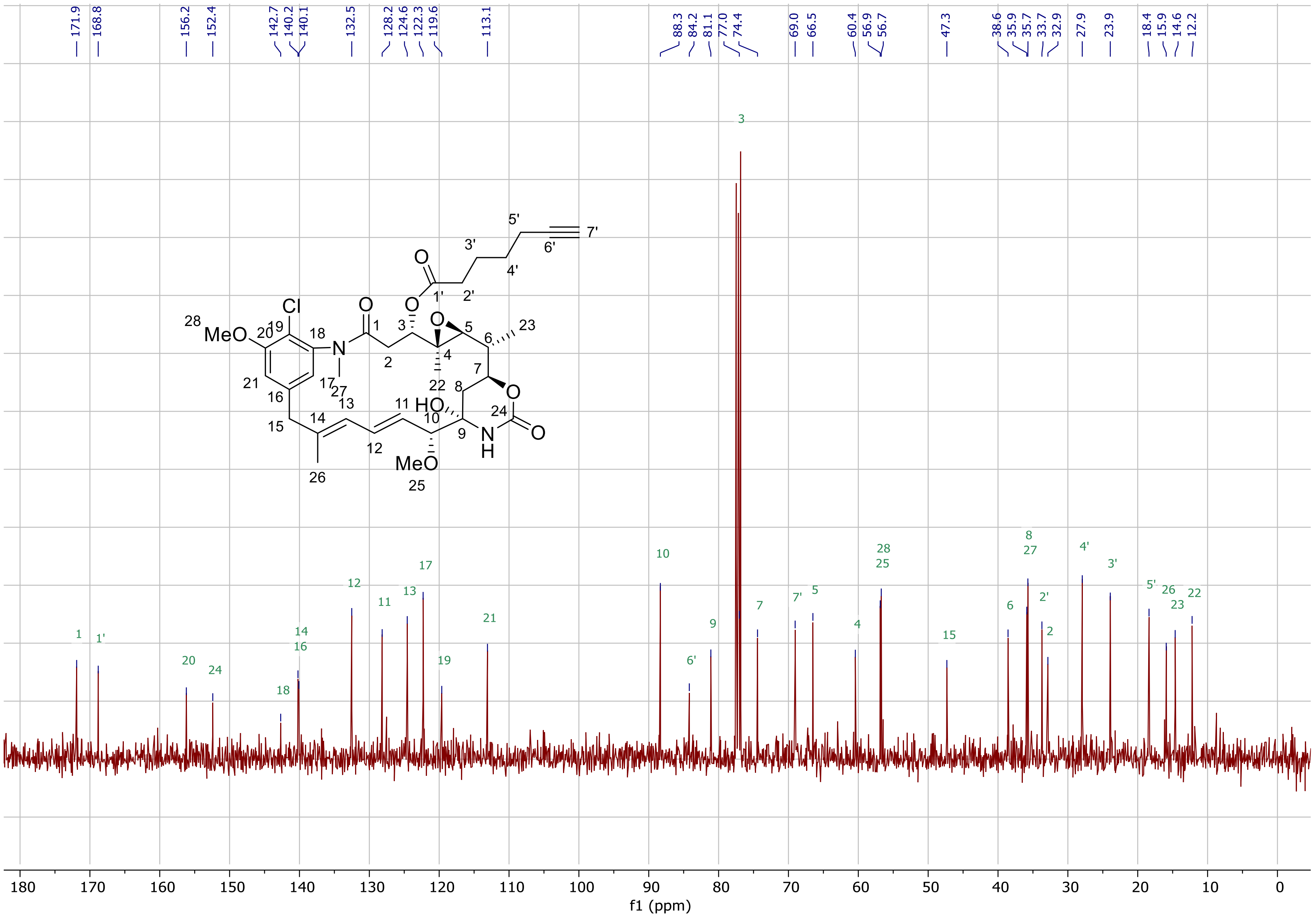

Figure S19. ^{13}C NMR spectrum (101 MHz, $CDCl_3$) of **4c**

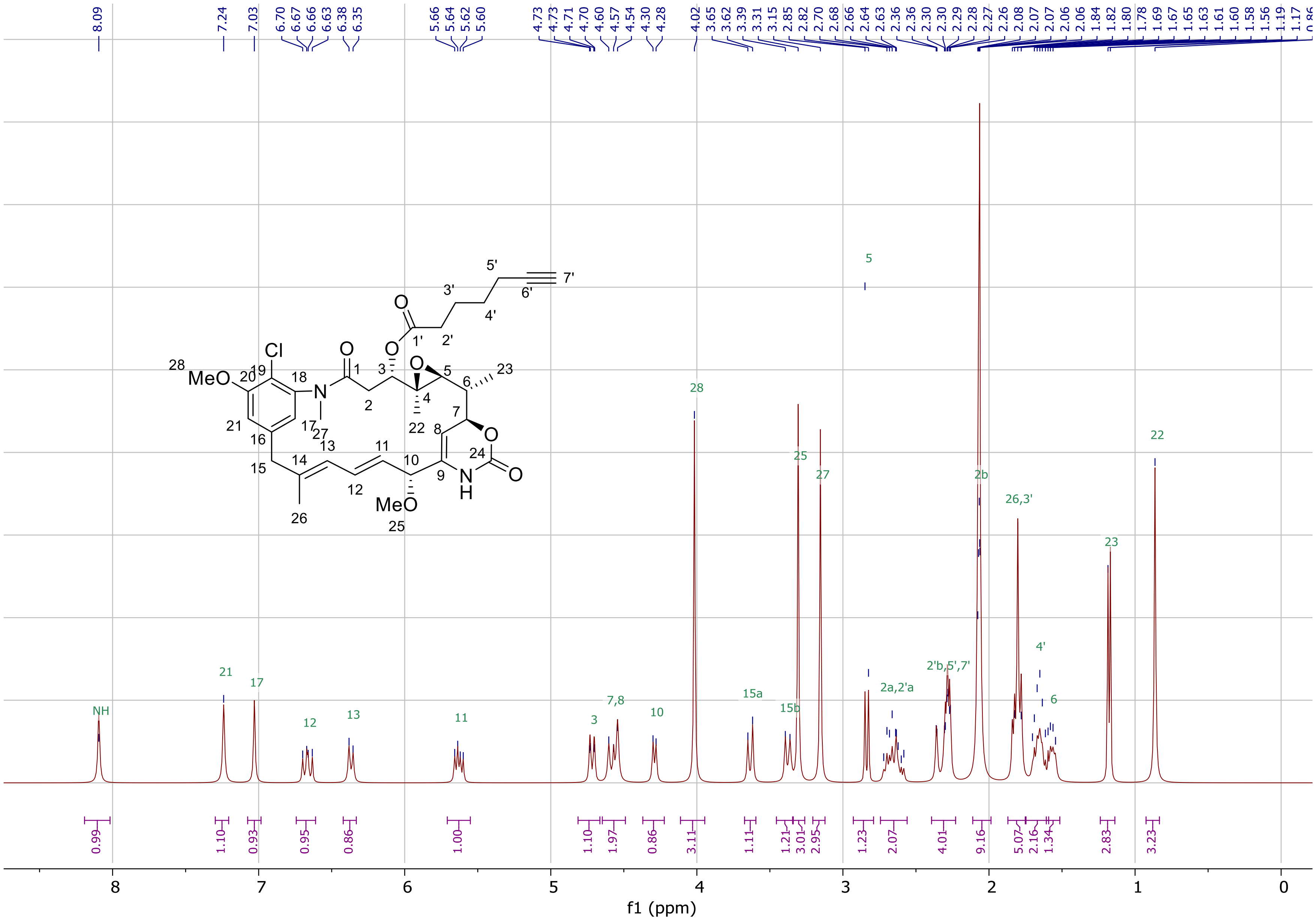

Figure S20. ^{1}H NMR spectrum (400 MHz, acetone-d6) of **5c**

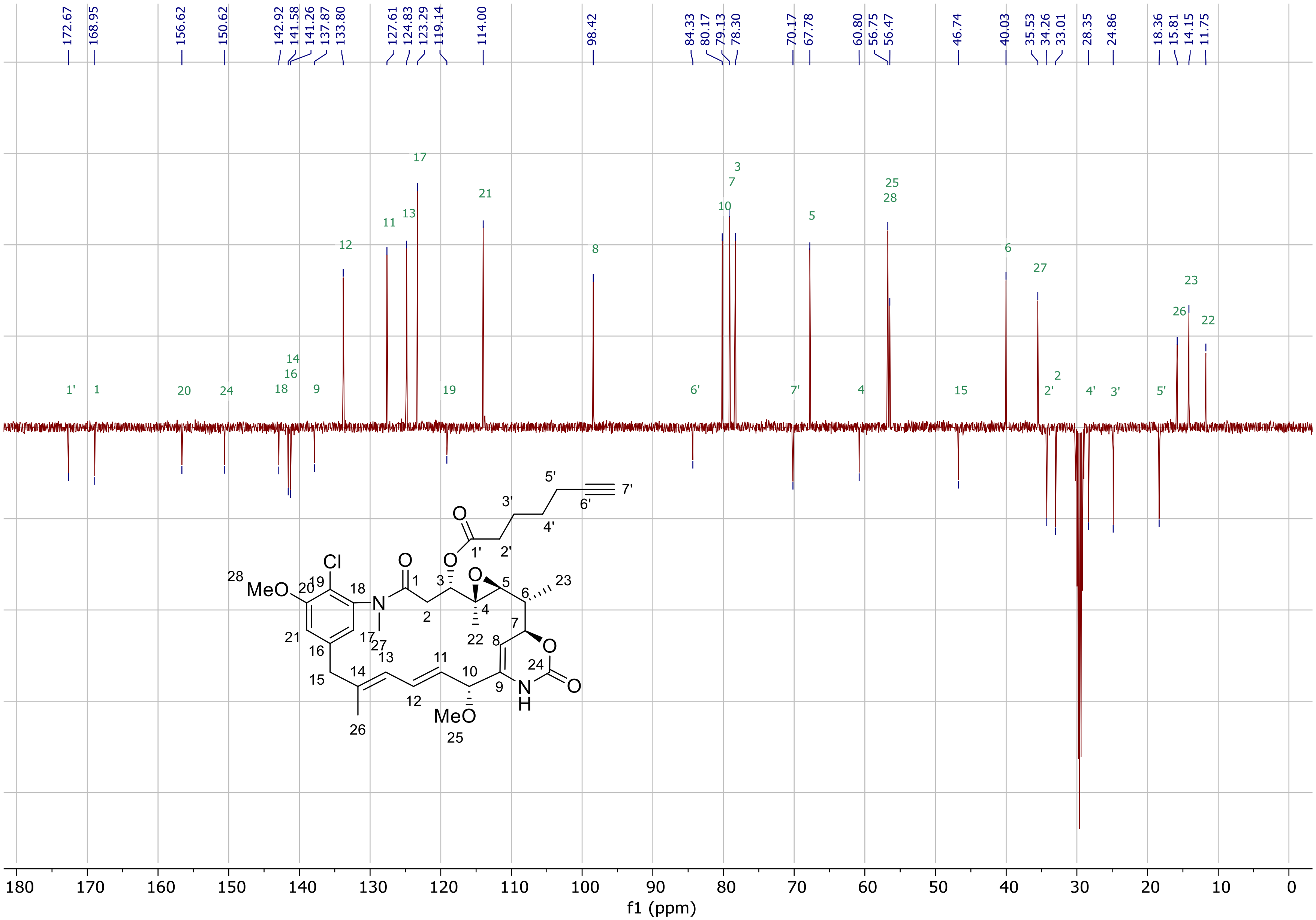

Figure S21. ^{13}C NMR APT spectrum (101 MHz, acetone-d6) of **5c**

Molecular modelling via Docking Analysis

The high-resolution crystallographic structure used for the docking studies of the synthesized maytansinoids was obtained from the Protein Data Bank (PDB) entry with ID 4TV8.[2] The molecular structure of each derivative was designed using the Build structure tool implemented in UCSF Chimera 1.14[3] by modifying the beforementioned crystal structure of the maytansine molecule. Docking input files for the target protein β-tubulin and the ligand structures were prepared with UCSF Chimera 1.14. The initial conformation of the macrocycle was derived from the maytansine crystal structure.

The calculations were performed using AutoDock Vina[4] which was run in UCSF Chimera 1.14 for the analysis and visualization of the results. AutoDock Vina considered all the atoms of the target site included in a cubic grid box with a grid spacing of 0.375 Å and a grid size of 22 Å for the geometry search within the docking process. The origin of the grid was positioned at the center of the maytansine coordinates in 4TV8. Finally, the resulting docking models were classified by the value of binding-free energy ∆G0 (kcal/mol) and the best solution with the lowest energy was selected for each derivative.

To validate the outcomes of the computational modelling experiments, the best conformer of each maytansinoid was superimposed to its corresponding crystal structure, which we reported within this manuscript (Figure S22). The structure comparison tool Match Maker in the software UCSF Chimera 1.14 was used for generating the superpositions.

The structural renderings were obtained using PyMol 2.3.4.[5]

We performed a quantitative analysis to study the similarity of the binding mode acquired by our models of molecules **3**, **4a-c**, **5a-c**, with respect to that observed in the crystal structures by calculating their root-meansquare deviation (RMSD) values (Table S5).

Table S5. RMSD values obtained when comparing the binding modes of the predicted models of each small molecule (**3**, **4a-c**, **5a-c**) to its corresponding solved crystal structure when bound to the maytansine-site of β-tubulin.

Maytansinoid	Maytansine	3	4a	4b	4c	5a	5b	5c
$RMSD$ (Å)	1.107	0.258	1.260	0.656	1.068	1.009	0.605	0.827

The results confirm the validity of the docking procedure and resulting modelled conformers.

Method

The best resulting docking conformers and the corresponding crystal structures of molecules **3**, **4a-c**, **5a-c**, were superimposed using the structure comparison tool Match Maker in the software UCSF Chimera 1.14

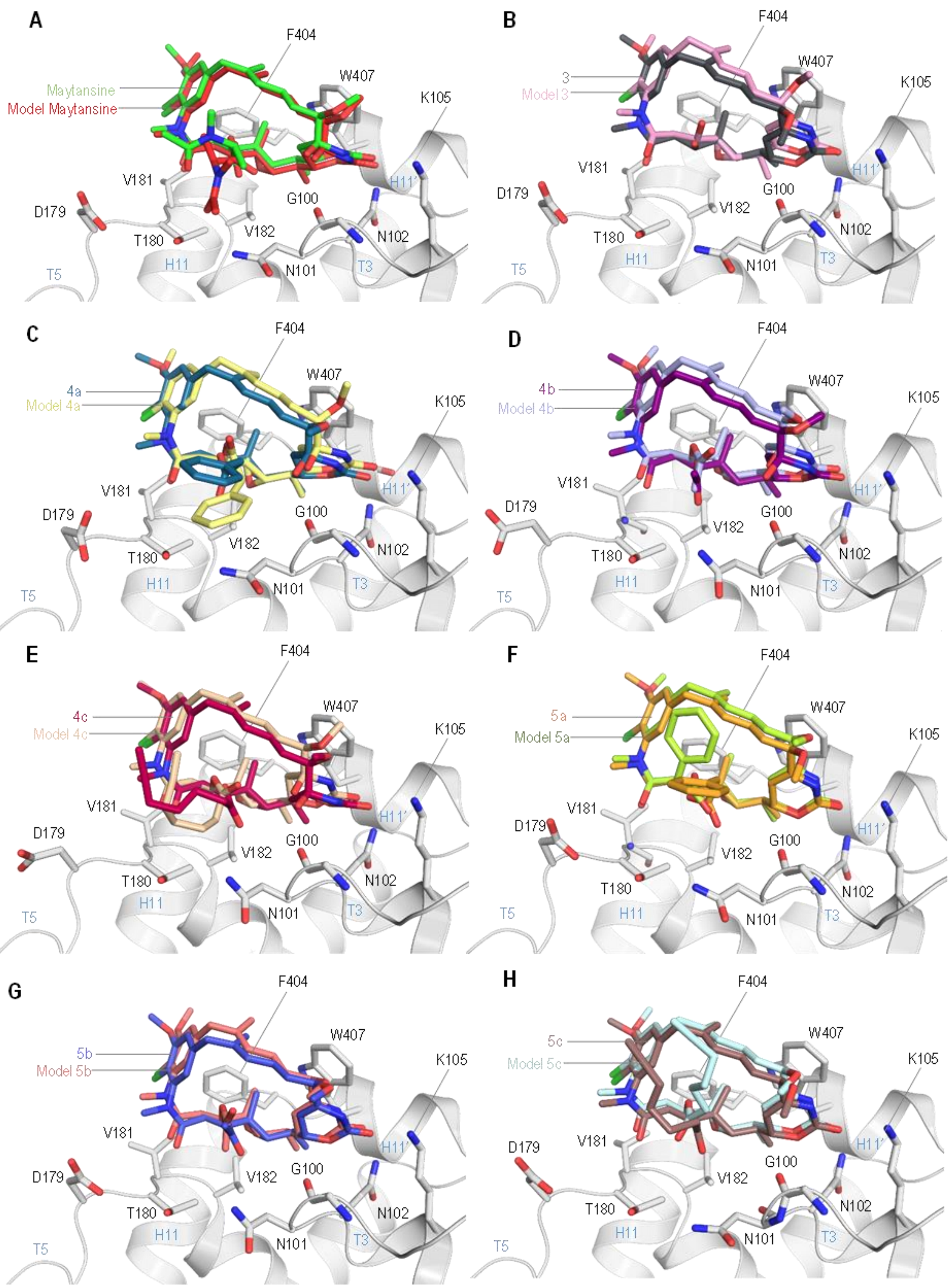

Figure S22. Superimposition of the crystallographic binding mode of compounds maytansine (A), 3 (B), 4a (C), 4b (D), 4c (E), 5a (F), 5b (G) and, 5c (H) and the best conformer predicted by Autodock Vina of each of these compounds when bound to the maytansine binding site of β-tubulin (grey).

Protein and chemicals for biochemistry

Purified calf brain tubulin and chemicals were obtained as previously described (Díaz, J. F., Menéndez, M. & Andreu, J. M. Thermodynamics of ligand-induced assembly of tubulin.[6,7]

Biochemistry

Polymerization of 25 μM tubulin in GAB buffer (3.4 M glycerol, 10 mM sodium phosphate (NaPi), 1 mM EGTA, 1 mM GTP, 6 mM $MgCl_2$, 1 mM GTP, pH 6.7) was monitored in the presence of the desired concentration of the ligand and was measured by turbidity at 350 nm employing a Multiskan plate reader (Thermo Fisher, Waltham, MA, USA).

Binding constants of the compounds to the maytansine site of tubulin were measured by competition with Fc maytansine as described.[1]

Cell Biology

Human A549 non-small lung carcinoma cells, human ovarian carcinomas A2780 and A2780AD (MDR overexpressing P-glycoprotein) were cultured at 37ºC in RPM-1640 supplemented with 10% fetal calf serum, 2mM L-glutamine, 1 mM sodium pyruvate, 40μg/ml gentamycin, 100 IU/ml penicillin and 100 μg/ml streptomycin in a 5% CO_2 air atmosphere and 37ºC. Antiproliferation assays were performed as described.[8] The statistical significance of differences in IC50 values were evaluated using the t-test option implemented in the Sigma Plot 13 software package (version 14.5, Systat Software, Inc., San Jose, CA, USA).

Indirect immunofluorescence images were obtained using A549 cells plated at a density of 130,000 cells/ml onto 12mm round coverslips, cultured overnight and treated with increasing amounts of the ligands or drug vehicle (DMSO) for 24h. DMSO was always less than 0.5%. Cells were permeabilized using Triton X-100 and fixed with 3.7 formaldehyde as previously described.[9] Cells were incubated with a DM1A mouse monoclonal antibody reacting against α-tubulin. After that, samples were washed, incubated with FITC goat anti-mouse antibody and 1mg/ml Hoechst 33342 was added to stain DNA. The slides were examined and photographed in a Zeiss Axioplan epifluorescence microscope and images were recorded using an ORCA-FLASH 4.2 cooled CCD camera.

Crystallization, data collection and structure determination

Crystals of the T_2R-TTL complex were prepared as previously described by Prota et al.[10] using the vapor diffusion method. Crystals were grown over two days at room temperature in buffer containing PEG 4K (3%), glycerol (4%), $MgCl_2$ (30 mM), $CaCl_2$ (30 mM), tyrosine (5 mM) and MES/imidazole pH 6.5 (100 mM). The maytansinoid compounds **3**, **4a-c, 5a-c** were soaked into the crystals over 6 hours, at 2.5 mM final concentration. Before flash-cooling in liquid nitrogen, crystals were successively transferred into cryo-protectant solutions containing the crystallization buffer with increased concentrations of PEG 4K (10%) and glycerol (16% and 22%). X-Ray diffraction data were collected at beamline X06DA of the Swiss Light Source (Paul Scherrer Institute, 5232 Villigen PSI, Switzerland) at 100 K and 1.0 Å wavelength. The obtained datasets were processed using XDS[11] and the structures were determined by the difference Fourier method in PHENIX[12] using the phases of a T_2R-TTL model in the absence of solvent molecules (modified from PDB ID: 5LXT). After one cycle of rigid-body refinement with grouped atomic displacement parameters (ADP), followed by several cycles of restrained individual coordinate and isotropic ADP refinement, ligand-shaped difference density was observed within the maytansine sites of all crystal structures. Waters were added using PHENIX[12] and geometry, Ramachandran and rotamer outliers were corrected in Coot.[13] The geometries of the compounds were energy-minimized in Moloc[14] and restraints for refinement were generated using the eLBOW tool in PHENIX[12]. The ligands were positioned within the difference density and refined in several cycles of restrained refinement. MolProbity[15] was used for model validation. In the final models only small root-mean-square deviations (rmsd) from the ideal bond lengths and angles were accepted. The molecular graphics were generated in PyMOL (the PyMOL Molecular Graphics System Version 2.3.2, Schrödinger, LLC).

Table S6 Data collection and refinement statistics for the T_2R-TTL-maytansinoid structures

	T_2R-TTL-**3**	T_2R-TTL-**4a**	T_2R-TTL-**4b**	T_2R-TTL-**4c**
Data collection				
Wavelength	1.00003	1.00000	1.00003	1.00003
Resolution range	47.98-2.3 (2.382- 2.3)	47.92 - 2.5 (2.589 - 2.5)	47.75 - 2.25 (2.33 - 2.25)	49.54 - 2.25 (2.33 - 2.25)
Space group	P 21 21 21	P 21 21 21	P 21 21 21	P 21 21 21
Unit cell	104.66 157.49 181.51	104.54 156.72 181.70	104.17 156.45 180.83	104.62 157.48 180.63
Total reflections	1830590 (186077)	1410952 (144378)	1915969 (182378)	1935205 (184195)
Unique reflections	133463 (13248)	103686 (10246)	140327 (13887)	140674 (13802)
Multiplicity	13.7 (14.0)	13.6 (14.1)	13.7 (13.1)	13.8 (13.3)
Completeness (%)	99.97 (100.00)	99.97 (100.00)	99.97 (100.00)	99.26 (98.48)
Mean I/sigma(I)	11.32 (0.94)	11.88 (1.18)	12.26 (0.90)	12.17 (1.04)
Wilson B-factor	50.05	51.58	46.68	44.37
R_{merge}	0.1903 (2.766)	0.2132 (2.267)	0.1936 (2.764)	0.1897 (2.34)
R_{meas}	0.1977 (2.87)	0.2216 (2.352)	0.2011 (2.876)	0.197 (2.434)
R_{pim}	0.05318 (0.7633)	0.05977 (0.6249)	0.05411 (0.7906)	0.05262 (0.661)
$CC_{1/2}$	0.998 (0.409)	0.997 (0.493)	0.998 (0.375)	0.998 (0.432)
CC*	1 (0.762)	0.999 (0.812)	1 (0.738)	0.999 (0.777)
Refinement				
Reflections used in refinement	133461 (13249)	103685 (10246)	140322 (13887)	140670 (13802)
Reflections used for R_{free}	6673 (663)	5184 (513)	7017 (695)	7032 (690)
R_{work}	0.1932 (0.3056)	0.1942 (0.3210)	0.1839 (0.3105)	0.1833 (0.2980)
R_{free}	0.2299 (0.3268)	0.2282 (0.3633)	0.2269 (0.3421)	0.2275 (0.3239)
CC_{work}	0.959 (0.674)	0.960 (0.716)	0.963 (0.659)	0.962 (0.704)
CC_{free}	0.950 (0.638)	0.945 (0.658)	0.946 (0.601)	0.942 (0.682)
Number of non-hydrogen atoms	18053	17725	18426	18500
macromolecules	17383	17221	17420	17403
ligands	214	224	224	230
solvent	456	280	782	867
Protein residues	2189	2153	2186	2187
RMS(bonds)	0.002	0.002	0.002	0.002
RMS(angles)	0.46	0.43	0.46	0.45
Ramachandran statistics				
favoured (%)	97.73	97.08	98.06	97.64
allowed (%)	2.27	2.87	1.94	2.36
outliers (%)	0.00	0.05	0.00	0.00
Rotamer outliers (%)	0.16	0.32	0.26	0.11
Clashscore	8.69	12.39	10.54	11.12
Average B-factor	65.67	64.23	61.51	58.57
macromolecules	65.95	64.35	61.76	58.82
ligands	64.57	67.38	59.49	57.06
solvent	55.75	54.38	56.37	53.77
Number of TLS groups	30	24	26	20

Statistics for the highest-resolution shell are shown in parentheses.

	T_2R-TTL-**5a**	T_2R-TTL-**5b**	T_2R-TTL-**5c**
Data collection			
Wavelength	1.00003	1.00003	0.99999
Resolution range	48.02 -2.32 (2.403-2.32)	47.92 - 2.25 (2.33 - 2.25)	48.06 - 2.75 (2.848 - 2.75)
Space group	P 21 21 21	P 21 21 21	P 21 21 21
Unit cell	104.91 157.91 181.46	105.08 158.60 180.44	105.12 158.40 181.38
Total reflections	1792987 (184037)	1956075 (186564)	1067807 (104119)
Unique reflections	130678 (12911)	142590 (14076)	79318 (7828)
Multiplicity	13.7 (14.3)	13.7 (13.3)	13.5 (13.3)
Completeness (%)	99.97 (99.99)	99.59 (99.13)	99.95 (100.00)
Mean I/sigma(I)	13.19 (0.87)	15.39 (1.42)	9.90 (0.93)
Wilson B-factor	51.83	42.59	62.95
R_{merge}	0.1847 (3.11)	0.1484 (1.728)	0.3426 (2.942)
R_{meas}	0.1919 (3.225)	0.1541 (1.797)	0.356 (3.06)
R_{pim}	0.05158 (0.8522)	0.0412 (0.4893)	0.09584 (0.8332)
$CC_{1/2}$	0.997 (0.359)	0.999 (0.589)	0.992 (0.369)
CC*	0.999 (0.727)	1 (0.861)	0.998 (0.734)
Refinement			
Reflections used in refinement	130673 (12911)	142590 (14076)	79316 (7828)
Reflections used for R_{free}	6533 (645)	7129 (704)	3966 (391)
R_{work}	0.1857 (0.3237)	0.1790 (0.2686)	0.1915 (0.3105)
R_{free}	0.2279 (0.3673)	0.2140 (0.2982)	0.2297 (0.3398)
$CC_{(work)}$	0.961 (0.639)	0.959 (0.789)	0.937 (0.613)
$CC_{(free)}$	0.958 (0.557)	0.944 (0.743)	0.913 (0.613)
Number of non-hydrogen atoms	18388	18528	17938
macromolecules	17406	17412	17397
ligands	230	224	217
solvent	752	892	324
Protein residues	2189	2188	2193
RMS(bonds)	0.002	0.002	0.002
RMS(angles)	0.46	0.47	0.47
Ramachandran statistics			
favored (%)	97.87	98.29	97.37
allowed (%)	2.08	1.71	2.49
outliers (%)	0.05	0.00	0.14
Rotamer outliers (%)	0.26	0.16	0.26
Clashscore	10.60	9.13	3.00
Average B-factor	67.50	56.58	73.03
macromolecules	67.89	56.79	73.44
ligands	66.75	54.36	69.97
solvent	58.70	53.04	53.23
Number of TLS groups	25	21	34

Statistics for the highest-resolution shell are shown in parentheses.

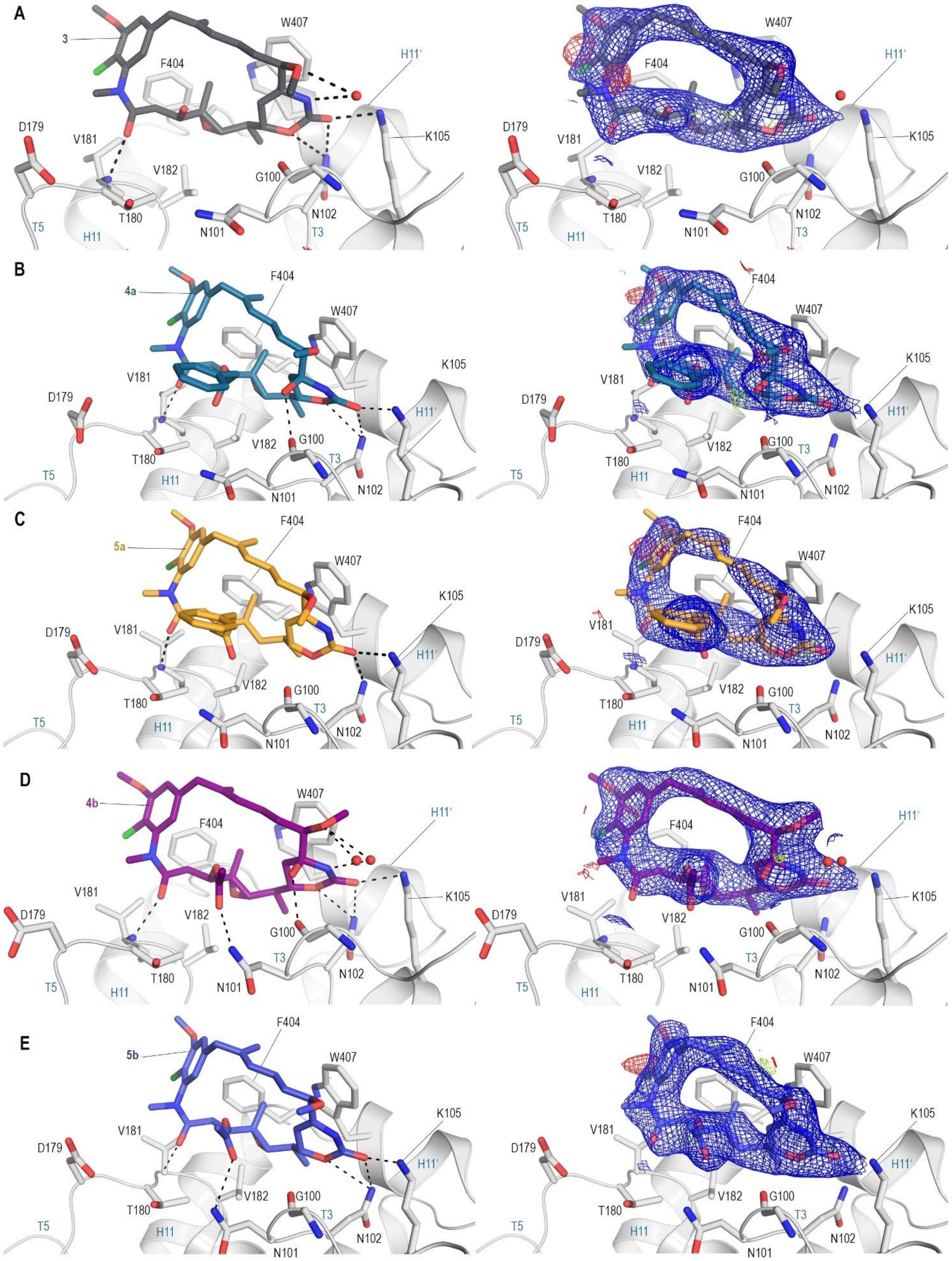
A
3
W407
F404
H11'
K105
D179
V181
V182
G100
T180
N102
N101
T5
H11
T3
B
4a
F404
W407
K105
V181
D179
H11'
V182
G100
T3
T180
T5
H11
N101
N102
C
5a
F404
W407
K105
D179
V181
H11'
V182
G100
T3
T180
T5
H11
N101
N102
D
4b
W407
F404
H11'
V181
K105
D179
V182
G100
T3
T180
N102
T5
H11
N101
E
5b
F404
W407
K105
V181
D179
H11'
V182
G100
T3
T180
N102
T5
H11
N101

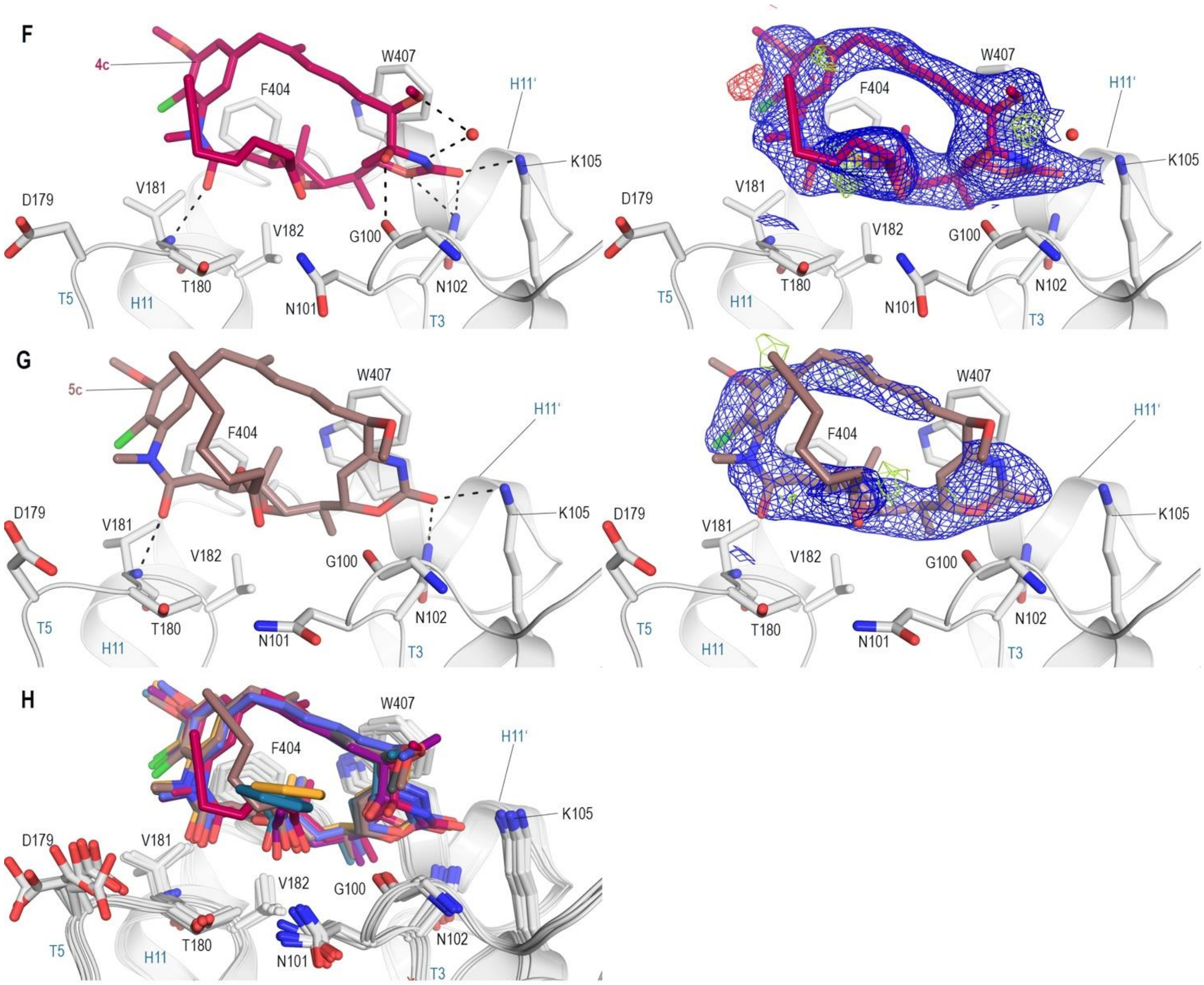

Figure S23 Analysis of the maytansinoid binding modes and their fit into the electron density

On the left side of each panel **A - G** the binding mode of the respective maytansinoid is shown and on the right the fit of the ligands into the electron density map. The tubulin molecule is in ribbon representation (grey) and the interacting residues and ligands are represented as sticks. Oxygen atoms are color coded in red, nitrogens are blue and the chlorine atom is in bright green. Hydrogen bonds are displayed as black dashed lines. The direct electron density (blue) is contoured at 1.0 σ and the mFo-Fc map (green/red) is contoured at ± 3.0 σ. Electron density maps are shown only around the maytansinoids to highlight their fit. In panel **H** the superposition of all investigated maytansinoids is displayed to show their common binding mode.

Supplemental References:

[1] G. Menchon, A. E. Prota, D. Lucena-Agell, P. Bucher, R. Jansen, H. Irschik, R. Müller, I. Paterson, J. F. Díaz, K. H. Altmann, M. O. Steinmetz, *Nat. Commun.* **2018**, *9*, 2106.

[2] A. E. Prota, K. Bargsten, J. F. Diaz, M. Marsh, C. Cuevas, M. Liniger, C. Neuhaus, J. M. Andreu, K. H. Altmann, M. O. Steinmetz, *Proc. Natl. Acad. Sci. U. S. A.* **2014**, *111*, 13817–21.

[3] E. F. Pettersen, T. D. Goddard, C. C. Huang, G. S. Couch, D. M. Greenblatt, E. C. Meng, T. E. Ferrin, *J. Comput. Chem.* **2004**, *25*, 1605–1612.

[4] O. Trott, A. J. Olson, *J. Comput. Chem.* **2009**, *31*, 455–461.

[5] W. L. T. P. M. G. S. DeLano, *DeLano Sci. LLC. Palo Alto, CA, USA,* **2002**, Available online: http://pymol.org (accessed on 1st Apr 2021).

[6] J. F. Díaz, J. M. Andreu, M. Menéndez, *Biochemistry* **1993**, *32*, 10067–10077.

[7] J. M. Andreu, in *Methods Mol. Biol. Vol. 137 (Ed J. Zhou) Ch. Microtubule Protoc.*, Humana Press Inc., **2007**, pp. 17–28.

[8] R. M. Buey, I. Barasoain, E. Jackson, A. Meyer, P. Giannakakou, I. Paterson, S. Mooberry, J. M. Andreu, J. F. Díaz, *Chem. Biol.* **2005**, *12*, 1269–1279.

[9] C. D. I. Biologicas, C. Superior, D. I. Cientificas, O. Chemistry, **1994**, 75–84.

[10] A. E. Prota, K. Bargsten, D. Zurwerra, J. J. Field, J. F. Díaz, K. H. Altmann, M. O. Steinmetz, *Science (80-.).* **2013**, *339*, 587–590.

[11] W. Kabsch, *Acta Crystallogr. Sect. D Biol. Crystallogr.* **2010**, *66*, 125–132.

[12] P. D. Adams, P. V. Afonine, G. Bunkóczi, V. B. Chen, I. W. Davis, N. Echols, J. J. Headd, L. W. Hung, G. J. Kapral, R. W. Grosse-Kunstleve, A. J. McCoy, N. W. Moriarty, R. Oeffner, R. J. Read, D. C. Richardson, J. S. Richardson, T. C. Terwilliger, P. H. Zwart, *Acta Crystallogr. Sect. D Biol. Crystallogr.* **2010**, *66*, 213–221.

[13] P. Emsley, B. Lohkamp, W. G. Scott, K. Cowtan, *Acta Crystallogr. Sect. D Biol. Crystallogr.* **2010**, *66*, 486–501.

[14] P. R. Gerber, K. Müller, *J. Comput. Aided. Mol. Des.* **1995**, *9*, 251–268.

[15] I. W. Davis, L. W. Murray, J. S. Richardson, D. C. Richardson, *Nucleic Acids Res.* **2004**, *32*, W615-619.
